# The Silexan in the Treatment Of Posttraumatic stress disorder (STOP) trial: protocol for a 12-week randomised controlled trial of adjunctive Silexan for PTSD

**DOI:** 10.1186/s12906-026-05312-7

**Published:** 2026-02-24

**Authors:** Greg Roebuck, Georgia M. Parkin,, Meaghan O’Donnell, Rahul Khanna, David Forbes, Winnie Lau, Shae Serpell, Haniya Al-Azzawi, Erin Santamaria, Mohammadreza Mohebbi, Ravi Iyer, Olivia Dean, Richard A. Kanaan, Malcolm Hopwood, Jessica E. Green, Michael Berk

**Affiliations:** 1 The Institute for Mental and Physical Health and Clinical Translation (IMPACT), School of Medicine, Deakin University and Barwon Health, PO Box 281, VIC Geelong, 3220 Australia; 2 Department of Psychiatry, Phoenix Australia – Centre for Posttraumatic Mental Health, University of Melbourne, Parkville, VIC Australia; 3 Biostatistics Unit, Deakin University, Geelong, VIC Australia; 4 Centre for Mental Health and Brain Sciences, Swinburne University of Technology, Hawthorn, VIC Australia; 5 Florey Institute for Neuroscience and Mental Health, University of Melbourne, Kenneth Myer Building, Parkville, Australia; 6 Austin Health, Heidelberg, VIC Australia; 7 SANE Australia, Melbourne, VIC Australia; 8 Department of Psychiatry, University of Melbourne, Parkville, VIC Australia; 9 Professorial Psychiatry Unit, Ramsay Clinic Albert Road, Melbourne, VIC Australia; 10 Mental Health Drugs and Alcohol Service, Barwon Health, Geelong, VIC Australia; 11 Orygen, the National Centre of Excellence in Youth Health, Parkville, VIC Australia

**Keywords:** Posttraumatic stress disorder, PTSD, Anxiety, Depression, Mental health, Silexan, Lavender oil

## Abstract

**Background:**

Posttraumatic stress disorder (PTSD) is a common and potentially debilitating psychiatric disorder. Current and emerging evidence-based treatments for PTSD have significant limitations. Silexan is an orally administered lavender oil preparation whose main constituents are the monoterpenoids linalool and linalyl acetate. It has a novel pharmacodynamic profile that includes inhibition of voltage-gated calcium channels and promotion of neuroplasticity. Silexan is effective in the treatment of Generalized Anxiety Disorder, subthreshold anxiety disorders and mild-to-moderate Major Depressive Disorder. It has an excellent safety and tolerability profile. Promising pilot data suggest that Silexan may be effective for PTSD. The Silexan in the Treatment Of Posttraumatic stress disorder (STOP) trial aims to investigate the effectiveness of adjunctive Silexan in PTSD.

**Methods:**

The STOP trial is a 12-week, parallel-arm, randomised, placebo-controlled, double-blind trial. Adults living in Australia who meet diagnostic criteria for PTSD according to the Mini-International Neuropsychiatric Interview-7 and have a score of ≥ 33 on the PTSD Checklist for DSM-5 will be eligible to participate. Participants will have the option of taking part in the trial remotely via videoconferencing software. They will receive either Silexan 160 mg or an inactive placebo daily for 12 weeks in addition to their usual prescribed medications. The primary outcome measure will be the change in total symptom severity score on the Clinician-Administered PTSD Scale for DSM-5 from baseline to week 12. Secondary outcome measures will include self-report measures of anxiety symptoms, depressive symptoms, somatic symptoms, sleep quality, subjective wellbeing, quality-of-life and problematic alcohol use. Additional secondary outcome measures will include objective measures of sleep, physical activity and physiology derived from data collected by actigraphy watches. The target sample size will be 278 participants.

**Discussion:**

If Silexan is found to be effective for PTSD, it is likely to be an attractive treatment option for many patients given its favourable safety and tolerability profile. Silexan is already licensed for use in 14 countries, enabling a rapid translation into clinical care.

**Trial registration:**

The STOP trial is registered on the National Institutes of Health clinicaltrials.gov website (ID: NCT06412757, URL: https://clinicaltrials.gov/study/NCT06412757, date of registration: 9 May 2024).

**Supplementary Information:**

The online version contains supplementary material available at 10.1186/s12906-026-05312-7.

## Background

Posttraumatic stress disorder (PTSD) is a psychiatric disorder characterised by re-experiencing symptoms, avoidance of trauma-related stimuli, negative changes in mood and cognitions and hyperarousal symptoms. It has an estimated global prevalence of 3.9% [1]. It usually follows a chronic course, with a median time to remission of 14 years [2]. It is associated with high levels of disability across all areas of functioning and higher healthcare costs per patient than depression [3, 4].

The most effective treatment for PTSD is trauma-focussed psychotherapy [5, 6]. However, this therapy requires patients to engage with their memories of the traumatic event, which many patients are unable to tolerate. Even in clinical settings with high levels of clinician training in evidence-based psychotherapies, only a small minority of individuals with PTSD receive trauma-focussed therapy [7]. Moreover, non-response is common, occurring in nearly 40% of cases [8]. Pharmacological options for PTSD include the selective serotonin reuptake inhibitors (SSRIs) sertraline, fluoxetine and paroxetine and the serotonin-noradrenaline reuptake inhibitor venlafaxine [5, 6]. These medications can reduce PTSD symptoms but they have small effect sizes, reflecting relatively modest clinical benefits [9]. Potential side effects include headache, nausea, vomiting, diarrhoea, agitation, tremor, insomnia, drowsiness and sexual dysfunction [10]. Sexual side effects occur in up to 80% of users and are a leading cause of non-adherence [11]. Methylenedioxymethamphetamine (MDMA)-assisted psychotherapy is a promising emerging treatment for PTSD [12]. However, clinical trials of MDMA-assisted therapy have been criticised on methodological grounds, including regarding the potential for functional unblinding and expectancy effects [13]. In addition, the treatment protocol followed in these trials is very resource-intensive, suggesting that there may be barriers to the translation of MDMA-assisted therapy into routine clinical care [14]. The limitations of current and emerging PTSD treatments highlight the need for new treatments that are effective, well-tolerated and accessible.

Silexan is a standardised lavender essential oil extract derived from steam distillation of *Lavandula angustifolia* flowers [15]. It is produced in the form of soft-gel capsules for oral administration. The main constituents of Silexan are the monoterpenoids linalool (36.8%) and linalyl acetate (34.2%) but it also contains lavandulol, lavandulyl acetate, borneol, eucalyptol and camphor in significant concentrations [16, 17]. Silexan and its main constituent, linalool, are potent inhibitors of voltage-gated calcium channels (VGCCs) [16, 18, 19]. At a concentration of 100 nmol/L, linalool reduces potassium-mediated calcium influx in murine synaptosomes by nearly 20% [16]. This action is similar to the mechanism of action of the gabapentinoids, which selectively inhibit P/Q-type VGCCs via their binding to the α2δ subunits of these channels. However, unlike the gabapentinoids, Silexan and linalool do not bind to the α2δ protein and show no selectivity for P/Q-type VGCCs, inhibiting N-type and T-type channels as well [16]. VGCCs are involved in regulating anxiety responses and have been proposed as potential targets for the pharmacological treatment of anxiety disorders [20–22]. L-type and T-type channels are also implicated in the mechanisms underlying the process of fear conditioning, which is believed to play a central role in PTSD pathogenesis [23, 24]. Silexan also promotes neuroplasticity, including by stimulating neurite outgrowth and synaptogenesis [25]. These neurotrophic effects are partly mediated by activation of protein kinase A and phosphorylation of the transcription factor cyclic AMP response element-binding protein (CREB), a common downstream effect of many antidepressant medications [26].

A growing body of evidence supports the efficacy of Silexan in the treatment of anxiety disorders, including Generalized Anxiety Disorder (GAD) and subthreshold anxiety disorders such as Anxiety Disorder Not Otherwise Specified and Mixed Anxiety and Depressive Disorder [27–29]. Doses of 80 mg daily and 160 mg daily have been used in clinical trials, with the higher dose showing greater efficacy [30]. Yap and colleagues [17] performed a meta-analysis pooling data from five randomised controlled trials with a total of 1,320 participants with GAD or a subthreshold anxiety disorder. They found that Silexan 160 mg was superior to Silexan 80 mg, paroxetine 20 mg, lorazepam 0.5 mg and placebo in reducing anxiety symptoms, as measured by the Hamilton Anxiety Rating Scale (HAM-A) [31]. Compared to placebo, Silexan 160 mg was associated with a weighted mean difference in HAM-A score of − 4.96 (95% confidence interval − 7.17 to − 2.76). More recently, Silexan has been found to be effective for mild-to-moderate Major Depressive Disorder (MDD) [32]. Silexan has an excellent safety and tolerability profile. Its only known adverse effects are mild gastrointestinal symptoms such as nausea, dyspepsia, eructation and lavender breath odour [17]. These side effects are uncommon, with individuals taking Silexan only 6% more likely than those taking placebo to experience side effects [33]. Silexan is licensed for use in 14 countries and is available as an over-the-counter preparation in Australia and the United States [33].

Pathological anxiety plays a significant role in the symptomatology of PTSD. Individuals with PTSD display acute anxiety in response to trauma-related cues, as well as chronic anxiety symptoms such as impaired concentration, poor sleep and irritability. This suggests that Silexan might be useful in the treatment of PTSD. However, to date, the only study of Silexan in PTSD has been a small, open-label pilot study [34]. This study found in a pre-post analysis that Silexan was associated with large reductions in anxiety and depressive symptoms in the subgroup of 30 participants who met criteria for PTSD.

The aim of the Silexan in the Treatment Of Posttraumatic stress disorder (STOP) trial is to investigate the effectiveness of adjunctive Silexan, compared with placebo, in improving PTSD symptoms in adults with PTSD. We hypothesise that adjunctive Silexan will be superior to placebo in improving PTSD symptoms. The STOP trial will also explore the effects of adjunctive Silexan on a number of secondary outcomes, including PTSD remission rates, anxiety symptoms, depressive symptoms, somatic symptoms, sleep quality, subjective wellbeing, quality-of-life and problematic alcohol use. Additional secondary outcomes will include measures of sleep, physical activity and physiology derived from data collected by actigraphy watches. The rationale for collecting physiological data is that PTSD is associated with physiological changes, including elevated resting heart rate, decreased heart-rate variability and elevated systolic and diastolic blood pressure [35, 36]. We hypothesise that adjunctive Silexan will be superior to placebo in improving all of these secondary outcomes.

## Methods

### Study design

The STOP trial is a 12-week, parallel-arm, randomised, double-blind superiority trial that aims to investigate the effectiveness of adjunctive Silexan (i.e. Silexan plus treatment-as-usual) compared with placebo (i.e. inactive placebo plus treatment-as-usual) in improving PTSD symptoms in adults suffering from PTSD.

### Study setting and personnel

All trial processes will be conducted at Phoenix Australia, a PTSD research and treatment centre based in Melbourne, Australia. Participants will be able to choose whether to attend trial assessments in person at the Phoenix Australia Traumatic Stress Clinic or to participate in them remotely using videoconferencing software. For online assessments, DocuSign will be used to obtain written informed consent, and self-report study measures will be emailed to participants as a Research Electronic Data Capture (REDCap) survey. All trial assessments will be conducted by researchers with an undergraduate degree in psychology or a related field and training and experience in administering psychological measures. Clinical supervision will be provided by the trial psychiatrist (GR), who will meet with the research team weekly.

### Study population

Inclusion criteria will be: (1) age 18 years or over, (2) fluent in English, (3) meets DSM-5 criteria for PTSD according to the Mini-International Neuropsychiatric Interview-7 for DSM-5 (MINI-7) [37] and (4) has a score ≥ 33 on the PTSD Checklist for DSM-5 (PCL-5) [38]. Exclusion criteria will be: (1) currently serving in the Australian Defence Force, (2) a lifetime history of a psychotic or bipolar disorder according to the MINI-7 or a diagnosis of Dissociative Identity Disorder (DID), (3) moderate or severe alcohol or other substance use disorder within the previous 3 months according to the MINI-7, (4) active suicidal or homicidal ideation, (5) a diagnosis of Borderline Personality Disorder (BPD) or a score of ≥ 7 on the Mclean Screening Instrument for Borderline Personality Disorder (MSI-BPD) [39], (6) acute or unstable medical illness or another serious medical condition that would make participation in the trial unsafe or inappropriate, (7) pregnancy, lactation or unwillingness to use an acceptable method of contraception, (8) commencement of a trauma-focussed psychotherapy (including Prolonged Exposure, Cognitive Processing Therapy and Eye Movement Desensitisation and Reprocessing) within the previous 3 months, (9) commencement or change in dose of psychoactive medications within the previous 4 weeks, (10) severe acquired brain injury, (11) ineligibility for public mental health services in Australia due to the person’s visa status or for any other reason, and (12) any other condition that in the opinion of the research team is likely to make completion of the trial requirements infeasible.

### Recruitment

Participants will be recruited through three sites: the Phoenix Australia Traumatic Stress Clinic, the Austin Health Psychological Trauma Recovery Service and the Ramsay Clinic Albert Road. All recruitment sites are tertiary mental health services based in Melbourne, Australia. Participants will also be recruited from the general Australian population via online advertisements. Advertisements will be placed on search engines and social media websites such as Google and Facebook as well as websites for groups at high risk of PTSD such as military veterans, police officers and emergency services workers.

### Eligibility assessment

After a potential participant contacts the intake team, a researcher will provide a brief overview of the trial via telephone. Written, informed consent to participate in the eligibility assessment will be obtained using DocuSign. The researcher will then email the potential participant three assessment measures: (1) the extended version of the Life Events Checklist for DSM-5 (LEC-5) [40], (2) the PCL-5 and (3) the MSI-BPD. If the potential participant’s PCL-5 score is ≥ 33, their MSI-BPD score is < 7 and their responses on the LEC-5 describe one or more traumatic events that meet Criterion A of the diagnostic criteria for PTSD in the fifth edition of the Diagnostic and Statistical Manual of Mental Disorders (DSM-5), a researcher will call them for a second intake phone call in which the researcher will conduct the remainder of the eligibility assessment. This includes administering the PTSD, Any Psychotic Disorder and Manic Episode modules of the MINI-7 to determine if the potential participant meets DSM-5 criteria for PTSD and if there is a lifetime history of a manic or psychotic disorder. It also includes collecting information regarding the other eligibility criteria. The researcher will also discuss the eligibility assessment with the trial psychiatrist (GR). If the potential participant is eligible for the trial, a baseline assessment will be arranged.

### Trial assessments

**Fig. 1:**
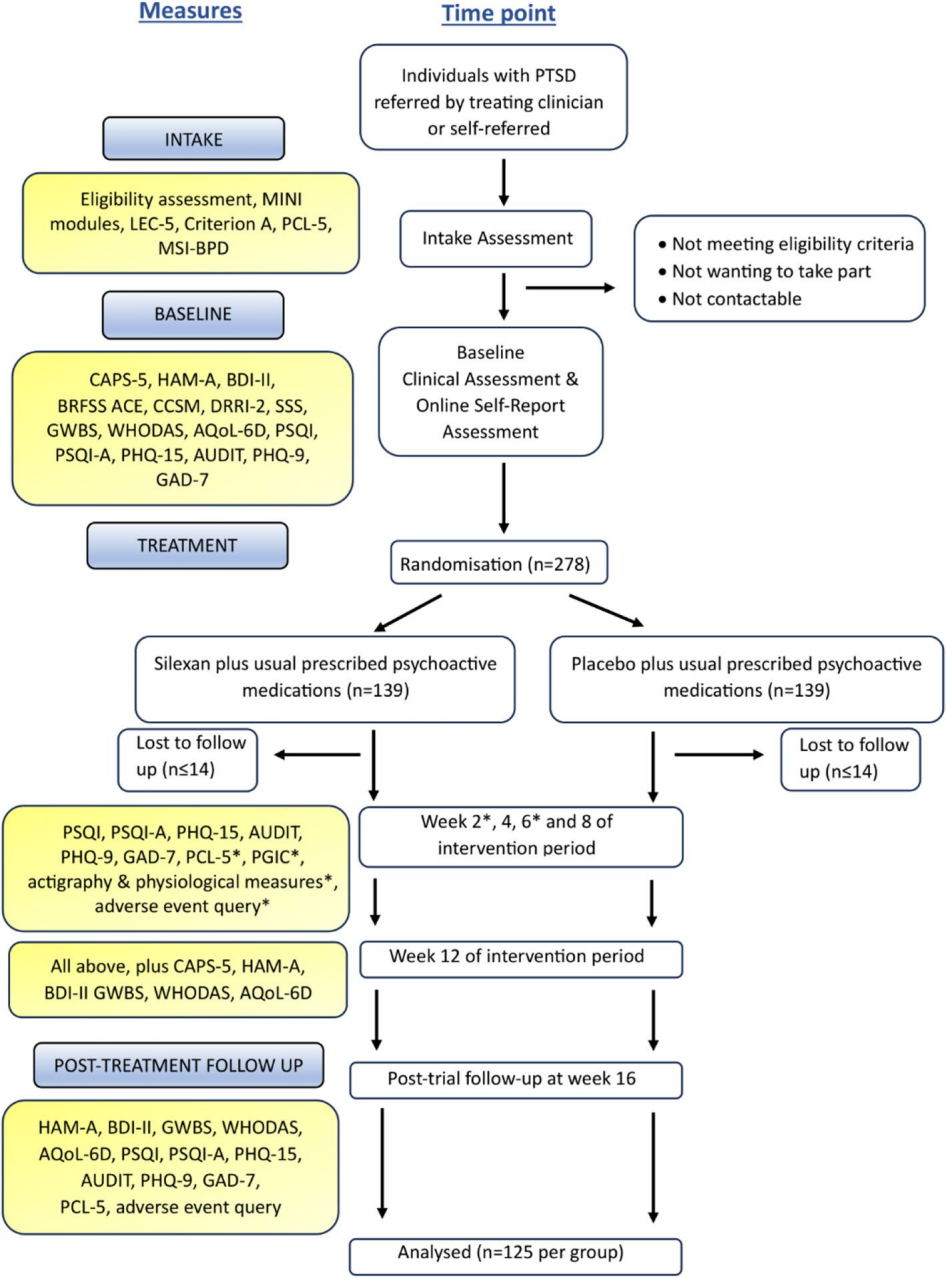
Study flow diagram*. Abbreviations*: CAPS-5, Clinician-Administered PTSD Scale for DSM-5; CGI, Clinical Global Impression Scale; PGIC, Patient Global Impression of Change; PCL-5, PTSD Checklist for DSM-5; HAM-A, Hamilton Anxiety Rating Scale; GAD-7, Generalized Anxiety Disorder-7; BDI-II, Beck Depression Inventory-II; PHQ-9, Patient Health Questionnaire-9; PHQ-15, Patient Health Questionnaire-15; PSQI, Pittsburgh Sleep Quality Index; PSQI-A, PTSD Addendum for PSQ; GWBS, General Well Being Schedule; WHODAS, World Health Organization (WHO) Disability Assessment Schedule; AQoL-6D, Assessment of Quality of Life-6D; I; AUDIT, Alcohol Use Disorders Identification Test

Participants will attend assessments at baseline, week 2, week 4, week 6, week 8, week 12 (primary endpoint) and week 16 (post-intervention follow-up). The baseline, week 12 and week 16 assessments include clinician-administered measures while the other assessments include only self-report measures. At the beginning of the baseline assessment, written informed consent to participate in the trial will be obtained by the trial psychiatrist (GR) following a discussion of the aims of the trial, the nature of the trial intervention and the potential benefits and risks of participation. Participants will also be asked to give their verbal consent to the audio recording of the clinician-assessed measures that are administered at baseline, week 12 and week 16. Approximately 10% of these recordings will be reviewed for fidelity purposes by the research team to ensure consistency and accuracy in assessment processes. Table 1 shows the study measures administered at each timepoint. Figure 1 is a study flow diagram showing the different stages of the trial.

**Table 1:** Study measures administered at each timepoint

	**Timepoint**							
	**Intake assessment**	**Baseline assessment**	**Intervention**					**Post-intervention**
			**Week 2**	**Week 4**	**Week 6**	**Week 8**	**Week 12**	**Week 16**
**Consent to participate in intake assessment**	X							
LEC-5	X							
PCL-5	X		X	X	X	X	X	X
MSI-BPD	X							
MINI-7*	X							
Other eligibility criteria	X							
**Consent to participate in trial**		X						
CAPS-5*		X					X	
HAM-A*		X					X	X
CGI*		X					X	X
BDI-II		X					X	X
BRFSS ACE		X						
CCSM		X						
Pregnancy test†		X						
DRRI-2‡		X						
SSS		X						
GWBS		X					X	X
WHODAS		X					X	X
AQoL-6D		X					X	X
PSQI & PSQI-A		X		X		X	X	X
PHQ-15		X		X		X	X	X
AUDIT		X		X		X	X	X
PHQ-9		X		X		X	X	X
GAD-7		X		X		X	X	X
PGIC			X	X	X	X	X	
Question regarding adverse events			X	X	X	X	X	X
Actigraphy and physiological measures		X	X	X	X	X	X	X
Allocation		X						
Dispensing of trial medication		X						

*Abbreviations*: *LEC-5* Life Events Checklist for DSM-5, *PCL-5* PTSD Checklist for DSM-5, *MSI-BPD* Mclean Screening Instrument for Borderline Personality Disorder, *MINI-7* Mini-International Neuropsychiatric Interview-7 for DSM-5, *CAPS-5* Clinician-Administered PTSD Scale for DSM-5, *HAM-A* Hamilton Anxiety Rating Scale, *CGI* Clinical Global Impression Scale, *BDI-II* Beck Depression Inventory-II, *BRFSS ACE* Behavioural Risk Factor Surveillance System Adverse Childhood Experience module, *CCSM* DSM-5 Level 1 Cross-Cutting Symptom Measure, *DRRI-2* Deployment Risk and Resilience Inventory-2, *SSS* Social Support Survey, *GWBS* General Well Being Schedule, *WHODAS* World Health Organization (WHO) Disability Assessment Schedule, *AQoL-6D* Assessment of Quality of Life-6D, *PSQI* Pittsburgh Sleep Quality Index, *PSQI-A* PTSD Addendum for PSQI, *PHQ-15* Patient Health Questionnaire-15; AUDIT, Alcohol Use Disorders Identification Test; PHQ-9, Patient Health Questionnaire-9; GAD-7, Generalized Anxiety Disorder-7; PGIC, Patient Global Impression of Change

^*^clinician-administered measure

^†^only for participants who are females of child-bearing potential

^‡^only for participants who have a history of military service

### Randomisation

Randomisation will occur after the baseline assessment. Participants will be randomised in a 1:1 ratio to the Silexan or placebo arms. The randomisation list will be created using permutated random-block randomisation (with block sizes of 2, 4 and 6). To ensure blinding and allocation concealment, random block sizes will be used and selected block sizes will be concealed from the research team. The generation of the allocation sequence using randomisation software will be conducted by an independent statistician who will also retain the random allocation list. Participants will be assigned sequentially to trial medication IDs and study assessors will not have access to the random allocation list.

### Intervention

Participants in the Silexan arm will receive two capsules of Silexan 80 mg daily for a total dose of 160 mg daily for 12 weeks in addition to their usual prescribed medications. Silexan is manufactured by Dr Willmar Schwabe GmbH & Co. KG (Willmar Schwabe) and is distributed in Australia under the name Seremind by Menarini Australia Pty Ltd. Participants in the placebo arm will receive an inactive matched placebo in addition to their usual medications. The trial medication will be dispensed to participants after completion of their baseline assessment. Participants who attend their baseline assessment remotely will have the trial medication sent to them by mail. No modifications to the allocated intervention will be permitted. Participants will be allowed to continue all concomitant treatment and care throughout the trial.

### Blinding/placebo

Blinding will be maintained by ensuring that the packaging, appearance and colour of the Silexan and placebo capsules are identical. This will be achieved via over-encapsulation of Silexan capsules. The over-encapsulated Silexan capsules and placebo capsules will be manufactured by Syntro Health. Consistent with previous clinical trials of Silexan, the lavender odour of the Silexan capsules when opened will be reproduced in the placebo capsules by adding 0.08 mg of lavender oil (0.05% of the daily treatment dose) to each placebo capsule [27–29, 32, 41]. Participants will be instructed to swallow the capsules unchewed. Blinding will also be maintained by identifying participants using anonymous participant ID numbers and not giving the research team access to the randomisation code. Unblinding will only occur if required on safety grounds, for example, if a serious adverse event occurs. The coordinating Principal Investigator (MB) will be responsible for determining if unblinding is required. The unblinded allocation list has been provided to an independent statistician at the sponsor site, an independent staff member at Phoenix Australia and the trial pharmacy. A Code Break Standard Operating Procedure has been established at the trial pharmacy.

### Assessing adherence

Delivery of trial medication sent by mail will be monitored using postal tracking numbers. In addition, medication bottles will include a QR code that participants can scan to document receipt and condition (damaged or undamaged) of the medication bottles. Participants will also receive a prepaid envelope to return the used medication bottles to the research team. Any capsules remaining in the returned bottles will be counted by the research team and the trial pharmacy prior to destruction.

### Outcome measures

The primary outcome measure will be the between-group difference in the change in the total symptom severity score on the Clinician-Administered PTSD Scale for DSM-5 (CAPS-5) from baseline to week 12 [42]. The CAPS-5 is regarded as the ‘gold standard’ for assessing PTSD symptoms in research settings [43]. Secondary outcome measures will include the between-group difference in the change in the proportion of participants meeting criteria for PTSD according to the CAPS-5 from baseline to week 12. They will also include the differences in the changes in self-reported PTSD symptoms (assessed by the PCL-5), anxiety symptoms, depressive symptoms, somatic symptoms, self-reported sleep quality, subjective wellbeing, quality of life, problematic alcohol use, clinical global impression and patient global impression from baseline to week 12. The between-group differences in the changes in these measures from baseline to week 16 will also be assessed. In addition, where appropriate, longitudinal trajectory analysis will be employed. Table 2 shows the primary and secondary outcome measures and the questionnaires used to assess each of these measures.

**Table 2:** Primary and secondary outcome measures and how they are assessed

Primary outcome measure	Method of assessment
Clinician-assessed PTSD symptoms	CAPS-5
**Secondary outcome measures**	
Self-report PTSD symptoms	PCL-5
Clinician-assessed anxiety symptoms	HAM-A
Self-report anxiety symptoms	GAD-7
Depressive symptoms	BDI-II, PHQ-9
Somatic symptoms	PHQ-15
Self-report sleep quality	PSQI, PSQI-A
Subjective wellbeing	GWBS
Quality of life	WHODAS, AQoL-6D
Problematic alcohol use	AUDIT
Clinical global impression	CGI
Patient global impression	PGIC
Heart rate	ActiGraph LEAP
Heart rate variability	ActiGraph LEAP
Blood pressure	ActiGraph LEAP
Respiratory rate	ActiGraph LEAP
Skin temperature	ActiGraph LEAP
Objective sleep measures (including total sleep time and sleep efficiency)	ActiGraph LEAP
Physical activity measures (including total movement and step count)	ActiGraph LEAP

*Abbreviations*: *CAPS-5* Clinician-Administered PTSD Scale for DSM-5, *CGI* Clinical Global Impression Scale, *PGIC* Patient Global Impression of Change, *PCL-5* PTSD Checklist for DSM-5, *HAM-A* Hamilton Anxiety Rating Scale, *GAD-7* Generalized Anxiety Disorder-7, *BDI-II* Beck Depression Inventory-II, *PHQ-9* Patient Health Questionnaire-9, *PHQ-15* Patient Health Questionnaire-15, *PSQI* Pittsburgh Sleep Quality Index, *PSQI-A* PTSD Addendum for PSQ, *GWBS*, General Well Being Schedule, *WHODAS* World Health Organization (WHO) Disability Assessment Schedule, *AQoL-6D* Assessment of Quality of Life-6D, I; *AUDIT* Alcohol Use Disorders Identification Test

### Actigraphy and physiological data

Actigraphy and physiological data will be collected using actigraphy watches. Participants will wear the Ametris LEAP (https://ametris.com/actigraph-leap), which is US FDA 510(k) cleared for the measurement of activity, sleep and mobility. The watch will be given to patients at their baseline assessment or, if they attend this assessment remotely, sent to them in the mail. They will be asked to wear it from the time they receive it until the end of the 16-week trial period. Data collected by the watch will include heart rate, heart rate variability, respiratory rate, skin temperature, sleep measures (including total sleep time and sleep efficiency) and physical activity measures (including total movement and step count). Additional secondary outcome measures will include the between-group differences in the changes in these markers of PTSD from baseline to week 12 and from baseline to week 16. Again, longitudinal trajectory analysis will be employed where appropriate. Table 2 includes a list of the physiology, sleep and physical activity secondary outcome measures.

### Sample size calculation

The target sample size will be 278 participants. Assuming a dropout rate of 10%, this will result in 250 participants who complete the trial, or 125 completers per treatment arm. For a two-tailed analysis with alpha set at 0.05, the study will have a power of 80% to detect a mean difference in the change from baseline to week 12 in CAPS-5 scores between the Silexan and placebo groups of 5 points, assuming a pooled standard deviation of 20 points. The expected effect size of *d* = 0.25 is similar to the effect size of SSRIs in PTSD (*d* = 0.28) [9], noting that a large randomised controlled trial recently found that Silexan and the SSRI sertraline had similar efficacy in treating mild-to-moderate Major Depressive Disorder [32].

### Participant remuneration

Participants will be reimbursed for expenses associated with participating in the study, including expenses associated with transport, parking, utilising a computer and recharging the actigraphy watches. A flat rate of $50 AUD will be provided per assessment attended by the participant, for a total of $350 AUD over the seven trial assessments. It may be provided in the form of a gift card.

### Monitoring of adverse events

Participants will be asked about adverse events at every study assessment. This will include questions about any health problems or problems relating to the trial medication or study questionnaires that they have experienced and any other notable recent events that have affected their mental or physical health. They will also be given a physical wallet insert that outlines the known side effects of Silexan and includes contact details for the research team and a 24-h phone number that they can use to contact the trial psychiatrist in an emergency. All reported adverse events will be monitored, recorded and reviewed by the trial psychiatrist. The study will also include a Data Safety and Monitoring Board (DSMB) comprised of an independent pharmacist, independent psychiatrist and independent statistician. The DSMB will meet every 6 months to review aggregate and individual participant data related to safety and to provide recommendations regarding the continuation or termination of the trial based upon this data. The DSMB charter will be made available to other researchers upon request. The conduct of the trial will also be monitored by an independent clinical trial monitor in accordance with a clinical trial monitoring plan. The clinical trial monitor will conduct visits remotely or in-person at the Phoenix Australia site every 6 months to verify that the conduct of the trial complies with Good Clinical Practice guidelines.

### Participant withdrawal

Participants will be free to withdraw from the study at any time upon their request. Withdrawal from the trial may occur in the following circumstances: (1) non-adherence to the trial medication for seven consecutive days, (2) pregnancy or cessation of effective contraception, (3) withdrawal of consent, (4) emergence of serious adverse effects and (5) emergence of new information about Silexan that means that withdrawal is in the participant’s best interests. In the event of withdrawal and cessation of the trial medication, data collection will continue if the participant consents to continue to attend study assessments.

### Data collection and management

Written informed consent to participate in the eligibility assessment and in the trial itself will be obtained using DocuSign unless the participant does not have access to a computer, in which case a hard copy consent form will be used. All study data will be entered by participants and researchers directly into electronic case report forms in REDCap. Data will be de-identified by associating each participant with a unique code identifier. Data from the actigraphy watches will be uploaded, stored and managed using Ametris’s secure, permissions-based CentrePoint cloud software program. Participants will be instructed to download the CentrePoint Connect phone application after receiving the watches. Data from the watches will be automatically uploaded to CentrePoint Connect via Bluetooth at regular intervals. All participant communication, and collection and storage of personal information for the purpose of trial enrolment, will be coordinated through secure University of Melbourne emails and servers with access restricted to the study team.

### Lived experience expertise

The study will include a Community Advisory Board (CAB) comprising two community members with lived experience of PTSD and two psychiatrists (RK and GR). The CAB is a strategic advisory committee that has the overarching aim of ensuring that the design and implementation of the trial meet the needs of and reflect the perspectives of those with lived experience of PTSD. It will meet quarterly via videoconference or in-person to review the progress of the trial and provide advice regarding its conduct.

### Statistical analysis

All analyses will be performed by an independent biostatistician who is blinded as to treatment allocation. The analysis will be conducted on an intention-to-treat basis, comprising all randomised participants. All intercurrent events will be handled using a treatment-policy strategy. In addition, a per-protocol analysis will be conducted, comprising all randomised participants who provided primary outcome data (baseline and week 12 CAPS-5) and completed 50% or more of the study visits (4 or more). A safety population analysis will also be conducted, comprising all randomised participants who received at least one treatment dose. Analyses will assess the impact of the intervention or, in other words, the between-group (intervention vs placebo) differential change from baseline to week 12 and other post-baseline timepoints in the primary and secondary outcome measures, over the study period. Analyses will use a multi-level Linear Mixed Model for Repeated Measures (MMRM) approach that will account for the correlation between assessment timepoints. The model will be adjusted for CAPS-5 scores obtained during the baseline assessment. Patterns of missing data in the primary and secondary outcomes will be investigated and if there is evidence of non-random missingness, appropriate sensitivity analyses will be performed [44]. Multiple imputations will be used to impute the missing data using all relevant demographics and other important chartists as auxiliary variables in the imputation process to mitigate the impact of non-random missingness. Important potential confounders such as sex, a history of childhood trauma, the presence of a comorbid psychiatric disorder and the use of other psychoactive medications, will be examined in additional exploratory sub-group analyses. A detailed statistical analysis plan has been prepared for the STOP trial and is included in the supplementary materials (Additional File 2).

### Data sharing plan and dissemination

The National Institute of Mental Health Data Archive (NDA) will be used as an online data-sharing repository (collection ID 5561). Participant data will be linked in the shared data repository using an assigned NDA Global Unique Identifier (GUID). The Participant Information and Consent Form (PICF) includes informed consent to NDA data-sharing. Study results will also be published in peer-reviewed scientific journals. Once the study has been completed by all participants, a notification letter will be sent to each participant revealing whether they were in the Silexan or placebo arm. Additionally, once the data have been analysed, participants will be sent a letter that includes a summary of the results of the trial.

## Discussion

The STOP trial is a 12-week, parallel-arm, randomised, placebo-controlled, double-blind trial that will investigate the effectiveness of adjunctive Silexan in adult PTSD. If Silexan is found to be an effective treatment for PTSD, it is likely to be an attractive treatment option for many patients given its favourable safety and tolerability profile. Silexan is already approved for use in the United States, the United Kingdom, Australia and many continental European countries. Patients with PTSD in these countries who are interested in trialling Silexan will therefore face relatively few barriers to accessing it.

The STOP trial will be the first clinical trial of Silexan conducted by a research team that is independent of the company that manufactures Silexan, Willmar Schwabe. All previous trials of Silexan have been sponsored by Willmar Schwabe. In their meta-analysis of randomised controlled trials of Silexan for anxiety disorders, Yap and colleagues [17] assessed the included studies as being at low risk of bias across almost all domains of bias except for the potential bias arising from the financial conflict of interest that existed due to the studies having been sponsored by Willmar Schwabe. If an independent research team finds that Silexan is an effective treatment for a psychiatric disorder in a large and rigorously conducted randomised controlled trial, this will be an important milestone in Silexan research.

Another point of difference between the STOP trial and previous Silexan trials is that the study population in the STOP trial is likely to be significantly more heterogeneous than those in previous trials. Other Silexan trials have generally excluded patients with active comorbid psychiatric disorders. For instance, the largest trial of Silexan for GAD excluded individuals with another DSM-IV-TR Axis I diagnosis (including MDD) within the previous six months [29]. In the STOP trial, the only psychiatric comorbidities that will result in exclusion are a psychotic or bipolar disorder, DID, BPD and recent moderate or severe alcohol or substance abuse. The rationale for including persons with comorbid disorders is that comorbidity is extremely common in PTSD, occurring in nearly 80% of cases [45]. Including participants with comorbid disorders improves the generalisability of the study’s findings. However, the heterogeneity of the study population could mean that it includes a larger proportion of participants with complex or treatment-resistant illness. This could decrease the likelihood that the trial will find a treatment effect of Silexan if one exists or, in other words, the trial’s assay sensitivity. In discussing comorbidity, it is important to note that both BPD and alcohol or drug abuse are very common PTSD comorbidities [46, 47]. Although it is not unusual for PTSD pharmacotherapy trials to exclude participants with these conditions, their exclusion does significantly reduce the trial’s external validity. If the STOP trial finds that Silexan is effective for PTSD, further trials in patients with comorbid BPD and alcohol or substance abuse will be required to determine if it is helpful for these populations as well.

The STOP trial will also be the first Silexan trial in which Silexan is given to participants adjunctively, or in addition to their other prescribed psychoactive medications. In previous trials, participants have been required to cease all psychoactive medications and undergo a washout period before commencing Silexan. The adjunctive nature of the trial intervention will have similar advantages and disadvantages as the inclusion of participants with psychiatric comorbidities. Advantages include that the study population is likely to be more representative of the overall population of people suffering from PTSD since it will include persons who are taking medications for PTSD or comorbidities and are unable or unwilling to cease these medications. Potential disadvantages include that the study population may include a larger number of participants with complex or treatment-refractory illness. In addition, it may include participants who are taking medications whose mechanism of action overlaps with, or interferes with, the mechanism of Silexan. Again, these factors could decrease the assay sensitivity of the trial. To assess if the adjunctive nature of the trial intervention has adversely impacted on the trial’s assay sensitivity, an exploratory subgroup analysis will be performed examining the effects of Silexan in those participants who received it as monotherapy.

Participants in the STOP trial will have the option of taking part in the trial remotely, with assessments occurring via videoconferencing software and the trial medication and actigraphy watches mailed to them. This is a relatively novel aspect of the trial design. Interest in decentralised clinical trials (DCTs) surged during the COVID-19 pandemic. Over the last six years, a number of DCTs have been performed in physical medicine [48]. However, other than one phase II trial in MDD [49], we are not aware of any fully remote pharmacotherapy trials in psychiatry with published results. No doubt remote participation might be inappropriate for some pharmacological agents, such as those with potentially dangerous side effects or narrow therapeutic windows. We anticipate that permitting remote participation will have significant advantages for the STOP trial. Social avoidance is very common in PTSD and many patients have difficulty attending public places such as research centres [50]. Allowing remote participation will likely remove significant barriers to participation for many patients. It will also enable people living outside the city of Melbourne, including those living in remote and rural areas, to take part in the trial. Online participation does create potential difficulties, however. Participants taking part in the trial remotely must use a computer, mobile phone or tablet to attend videoconferences and fill out REDCap questionnaires, necessitating some degree of computer literacy. There is also the potential for computer problems to interfere with trial assessments, for environmental distractions to arise during assessments and for problems in the postal system to delay delivery of the trial medication. In addition, it is possible that vital data might be lost over the videoconferencing format. For instance, it might be more difficult for researchers to assess a participant’s affect or behaviour in a videoconference. The experience of the investigators and participants in the STOP trial will provide valuable information regarding the advantages and disadvantages of DCTs in psychiatry.

A final point of novelty of the STOP trial is the use of actigraphy watches to collect data regarding participants’ physiology, sleep and physical activity levels. This also appears to be innovative for a PTSD pharmacotherapy trial. Although some previous trials have used wearable devices to collect data concerning sleep outcomes, we are not aware of any previous trials using such devices to collect data concerning physiological and activity-related markers putatively related to PTSD. Psychiatry research has been criticised for relying too heavily on clinician- and patient-rated symptom scales, which are inherently subjective and may not correspond well with the outcomes that actually matter to patients, carers and healthcare systems [51]. Such scales contrast with the objective outcome measures typically used in other areas of medical research. To date, the only objective data collected in psychiatry trials have tended to be blood, neuroimaging and electrophysiological data. The aim in collecting these data has been to obtain measures of the underlying biological processes responsible for psychiatric disorders. However, the pathophysiology of mental illness remains largely unknown and the search for biomarkers of mental illness has been a disappointment [52]. Wearable devices offer another means of collecting objective outcome measure data in psychiatry trials. They present at least two points of novelty. First, in at least some cases, rather than assessing underlying biological processes, they provide objective measures of psychiatric symptoms such as physical inactivity and poor sleep. This objective data may complement clinician-administered or self-report measures of these symptoms. For instance, in the STOP trial, the self-report data concerning sleep provided by participants will be complemented by the objective sleep data collected by the actigraphy watches. Secondly, wearable devices are likely to provide many more points of measurement than blood, neuroimaging or electrophysiology investigations, which are time-consuming and costly to perform, usually require a laboratory setting and can usually only be done a small number of times. The results of the STOP trial may shed light on whether or not this is a more promising approach to the collection of objective outcome measure data in psychiatry trials.

## Supplementary Information

Supplementary Material 1: STOP Trial protocol, version 9.

Supplementary Material 2: STOP Trial Statistical Analysis Plan, version 2.

## Data Availability

No datasets were generated or analysed during the current study.
