# Supplementary material for: The Silexan in the Treatment Of Posttraumatic stress disorder (STOP) trial: protocol for a 12-week randomised controlled trial of adjunctive Silexan for PTSD
Source: BMC Complement Med Ther. 2026 Feb 24;26:122. doi: 10.1186/s12906-026-05312-7 (PMC13041431; doi:10.1186/s12906-026-05312-7)
Supplement: Supplementary file 1 — Supplementary Material 1: STOP Trial protocol, version 9. [file 12906_2026_5312_MOESM1_ESM.docx]

PROTOCOL­­

| Silexan in the Treatment Of Posttraumatic stress disorder (STOP) trial |
| --- |
| Public Title: The STOP Trial  University of Melbourne HREC Reference/Protocol # 29307  Version # 9.0  Date: 28/11/25 |
|  |
| Contact person: Dr Greg Roebuck  Email: g.roebuck@deakin.edu.au  Postal address: Deakin University, School of Medicine, PO Box 281, Geelong, Victoria, 3220  Contact for scientific queries: stop-trial@unimelb.edu.au  Coordinating Investigator:  Prof Michael Berk, Deakin University  Investigators:  Prof Richard Kanaan, Austin Health  Dr Rahul Khanna, Austin Health and Phoenix Australia  Dr Greg Roebuck, Deakin University and Phoenix Australia  Dr Mohammadreza Mohebbi, Deakin University  Prof David Forbes, Phoenix Australia  Prof Meaghan O’Donnell, Phoenix Australia  Prof Malcolm Hopwood, Phoenix Australia and Albert Road Clinic  Associate Prof Olivia Dean, Deakin University  Mr William Jensen |
| CONFIDENTIAL  This document is confidential and the property of Deakin University, The University of Melbourne, Austin Health, and Albert Road Clinic. No part of it may be transmitted, reproduced, published, or used without prior written authorisation from the institutions.  STATEMENT OF COMPLIANCE  This study will be conducted in compliance with all stipulation of this protocol, the conditions of the ethics committee approval, the NHMRC National Statement on ethical Conduct in Human Research (2007) and the ICH Guidelines for Good Clinical Practice (ICH-GCP).  SOURCE OF MONETARY OR MATERIAL SUPPORT  This trial is supported by a Fiscal Year 2022 (FY22) Traumatic Brain Injury and Psychological  Health Research Program (TBIPHRP) Clinical Trial Award - Research Level 2, from the Department of Defense (DoD) office of the Congressionally Directed Medical Research Programs (CDMRP). This support has been awarded to the coordinating PI, Professor Michael Berk, at the Institute for Mental and Physical Health and Clinical Translation (IMPACT), Deakin University.  PRIMARY SPONSOR  Deakin University  ROLE OF PRIMARY SPONSOR AND FUNDER  Deakin University, and separately the DoD CDMRP, will not have any role in collection, management, analysis, and interpretation of data; writing of the report; and the decision to submit the report for publication, nor ultimate authority over any of these activities. Inclusion of select assessment modules (see section 7.5.1), and use of a data-sharing repository (see section 11.3), were required by the DoD CDMRP; exclusive of this, neither Deakin University, nor the DoD CDMRP, will have any role in study design.  **Document History:**   \| **Version** \| **Date of Change** \| **Summary of Changes** \| \| --- \| --- \| --- \| \| Version 1.0 \| 20/02/2024 \| Original submission \| \| Version 2.0 \| 05/03/2024 \| Clarification made to section 12.1 following HREC pre-submission review \| \| Version 3.0 \| 10/04/2024 \| Clarifications/amendments made to eligibility, actigraphy, risk, data storage, in response to HREC reviewer comments \| \| Version 4.0 \| 26/06/2024 \| Addition of Trialfacts recruitment, Addition of self-reported height and weight to section 16.3, Amendment of secondary outcome 2 (section 5.3) to improve clarity, Update of section 2 to match section 7.3.5, correction of typos and addition of 1800 RESPECT in PLS, Addition of medical record declaration to trial consent form, Clarification that qualified clinical psychologist and psychiatrist researchers are not required to complete training courses intended for researchers without such qualifications (section 3.5); clarification of protocol and associated document wording and exclusion criteria as requested by the DDVA HREC \| \| Version 5.0 \| 19/08/2024 \| Amendment to PLS wording as requested by the DDVA HREC; replacement of audio recording of 10% of intake assessments with recording of 10% of primary outcome measure (CAPS-5) assessments; Removal of Barwon Health logo from all participant-facing material; Movement of MINI alcohol and substance abuse questions from intake clinician interview to intake self-report questionnaire; Amendment of Actigraph information sheet; Addition of Actigraph registration information sheet; Addition of handedness question to demographic questionnaire; Replacement of MADRS with BDI-II \| \| Version 6.0 \| 22/11/2024 \| Expansion of recruitment and study participation to inter-state individuals; amendment of clinical trial monitoring section (section 13.1); addition of new public- and clinician-facing flyers; addition of bottle wrap confirmation to lip of trial medication bottles \| \| Version 7.0 \| 28/01/2025 \| Removal of week 16 CAPS-5 assessment; amendment of exclusion criterion 6 \| \| Version 8.0 \| 20/05/2025 \| Amendment of 7.5.3 (Schedule of assessments) and 8.2.1 (Monitoring treatment adherence) to dispense all medication bottles to participant following the baseline visit \| \| Version 9.0 \| 28/11/25 \| Amendment to sample size and duration of study \| |

# Table of Contents

[Table of Contents 4](#_Toc173840715)

[1. Acronyms 1](#_Toc173840716)

[2. Study synopsis 4](#_Toc173840717)

[3. Administrative information 7](#_Toc173840718)

[3.1 Project team roles and responsibilities 7](#_Toc173840719)

[3.2 Conflict of interest 10](#_Toc173840720)

[3.3 Disclosure of intellectual property use 10](#_Toc173840721)

[3.4 Expected duration of study 10](#_Toc173840722)

[3.5 Assessors 11](#_Toc173840723)

[4. Introduction and background information 13](#_Toc173840724)

[4.1 Silexan – pharmacology 13](#_Toc173840725)

[4.2 Anxiolytic effects of Silexan 14](#_Toc173840726)

[4.3 Antidepressant and other effects of Silexan 15](#_Toc173840727)

[4.4 Safety and tolerability of Silexan 15](#_Toc173840728)

[4.5 Rationale for potential utility of Silexan in PTSD 15](#_Toc173840729)

[4.6 Pilot evidence of efficacy and tolerability of Silexan in PTSD 15](#_Toc173840730)

[4.7 Impact and relevance to military mental health 16](#_Toc173840731)

[5. Study Objectives 16](#_Toc173840732)

[5.1 Study aim 16](#_Toc173840733)

[5.2 Study hypotheses 16](#_Toc173840734)

[5.3 Study Outcome Measures 17](#_Toc173840735)

[5.4 Potential benefits and impact 17](#_Toc173840736)

[6. Study design and methods 18](#_Toc173840737)

[6.1 Study population 19](#_Toc173840738)

[7. Recruitment procedure 19](#_Toc173840739)

[7.1 Recruitment, referral and advertising study sites 19](#_Toc173840740)

[7.2 Research project setting 20](#_Toc173840741)

[7.3 Eligibility 21](#_Toc173840742)

[7.3.1 Initial contact 21](#_Toc173840743)

[7.3.2 Electronic consent to intake assessment 22](#_Toc173840744)

[7.3.3 Intake assessment 22](#_Toc173840745)

[7.3.4 Inclusion criteria 22](#_Toc173840746)

[7.3.5 Exclusion criteria 22](#_Toc173840747)

[7.3.6 Acceptable contraception 23](#_Toc173840748)

[7.4 Written informed consent 24](#_Toc173840749)

[7.5 Baseline assessment measures 24](#_Toc173840750)

[7.5.1 Use of videoconference programs 25](#_Toc173840751)

[7.5.2 Baseline assessments 25](#_Toc173840752)

[7.5.3 Schedule of assessments 30](#_Toc173840753)

[8. Randomisation, blinding and intervention 32](#_Toc173840754)

[8.1 Randomisation and blinding 32](#_Toc173840755)

[8.2 Intervention treatment 32](#_Toc173840756)

[8.2.1 Monitoring treatment adherence 33](#_Toc173840757)

[8.3 Intervention assessments 33](#_Toc173840758)

[8.3.1 Actigraphy and physiological measures 33](#_Toc173840759)

[8.4 GP Letter 34](#_Toc173840760)

[8.5 Participant remuneration 34](#_Toc173840761)

[9. Participant withdrawal 35](#_Toc173840762)

[9.1 Determination of loss-to-follow-up 35](#_Toc173840763)

[10. Adverse events and risks 36](#_Toc173840764)

[10.1 Definitions 36](#_Toc173840765)

[10.1.1 Causality 37](#_Toc173840766)

[10.2 Eliciting adverse event information 37](#_Toc173840767)

[10.3 Safety reporting 38](#_Toc173840768)

[10.3.1 Reporting of AEs 38](#_Toc173840769)

[10.3.2 Reporting of SAEs 38](#_Toc173840770)

[10.4 Risks 39](#_Toc173840771)

[10.4.1 Mental state 39](#_Toc173840772)

[10.4.2 Management of suicidality 39](#_Toc173840773)

[10.4.3 Treatment-related direct effect 40](#_Toc173840774)

[10.4.4 Treatment-related indirect effect 40](#_Toc173840775)

[10.4.5 Legal risk 41](#_Toc173840776)

[10.4.6 Management of pregnancy 41](#_Toc173840777)

[10.5 Benefits 41](#_Toc173840778)

[11. Data collection 41](#_Toc173840779)

[11.1 Data capture methods 41](#_Toc173840780)

[11.1.1 Actigraph data collection 42](#_Toc173840781)

[11.2 Data storage 42](#_Toc173840782)

[11.2.1 Electronic data storage (REDCap) 42](#_Toc173840783)

[11.2.2 Other electronic data 43](#_Toc173840784)

[11.2.3 Paper source documents 43](#_Toc173840785)

[11.3 Data sharing plan 43](#_Toc173840786)

[12. Data analysis 43](#_Toc173840787)

[12.1 Analysis of quantitative data 44](#_Toc173840788)

[12.2 Power calculations 44](#_Toc173840789)

[12.3 Feasibility 44](#_Toc173840790)

[13. Study oversight 44](#_Toc173840791)

[13.1 Clinical monitoring plan 44](#_Toc173840792)

[13.2 Data Safety Monitoring Board 46](#_Toc173840793)

[13.3 Community-based participatory research 46](#_Toc173840794)

[14. Results 47](#_Toc173840795)

[15. References 48](#_Toc173840796)

[16. Appendices 53](#_Toc173840797)

[16.1 Therapeutic Goods Administration – ARTG Entry – Seremind 53](#_Toc173840798)

[16.2 Food and Drug Administration Approval of Lavender 54](#_Toc173840799)

[16.3 Demographics 55](#_Toc173840800)

[16.4 McLean Screening Instrument for BPD 60](#_Toc173840801)

[16.5 Life Events Checklist for DSM-5 (LEC-5) with Criterion A 61](#_Toc173840802)

[16.6 Beck’s Depression Inventory 63](#_Toc173840803)

[16.7 Hamilton Anxiety Rating Scale (HAM-A) 64](#_Toc173840804)

[16.8 DSM-5-TR Self-Rated Level 1 Cross-Cutting Symptom Measure—Adult (CCSM) 65](#_Toc173840805)

[16.9 Clinical Global Impression Scale/ Patient Global Impression of Change 66](#_Toc173840806)

[16.10 World Health Organization Disability Assessment Schedule 2.0 67](#_Toc173840807)

[16.11 Pittsburgh Sleep Quality Index 68](#_Toc173840808)

[16.12 Patient Health Questionnaire-15 69](#_Toc173840809)

[16.13 Deployment Risk & Resilience Inventory-2 70](#_Toc173840810)

[16.14 Assessment of Quality of Life-6D scale 71](#_Toc173840811)

[16.15 Behavioural Risk Factor Surveillance Survey Adverse Childhood Experience Module 72](#_Toc173840812)

[16.16 Social Support Survey 73](#_Toc173840813)

[16.17 Patient Health Questionnaire – 9 75](#_Toc173840814)

[16.18 PTSD Checklist for DSM-5 (PCL-5) 76](#_Toc173840815)

[16.19 General Well-Being Schedule 77](#_Toc173840816)

[16.20 Generalized Anxiety Disorder – 7 78](#_Toc173840817)

[16.21 Alcohol Use Disorders Identification Test 79](#_Toc173840818)

[16.22 Clinician-Administered PTSD Scale (CAPS-5) 80](#_Toc173840819)

[16.23 Personally Identifiable Information for the generation of NDA Global Unique Identifier (GUID) 81](#_Toc173840820)

# Acronyms

**Abbreviation Description**

APS Australian Psychological Society

AQoL-6D Assessment of Quality of Life-6D scale

AUDIT Alcohol Use Disorders Identification Test

BDI-II Beck Depression Inventory-II

BRFSS ACE Behavioural Risk Factor Surveillance Survey Adverse Childhood Experience Module

BPD Borderline Personality Disorder

CAPS-5 Clinician-Administered PTSD Scale for DSM-5

CCSM DSM-5 Level 1 Cross-Cutting Symptom Measure

CGI Clinical Global Impression scale

CREB cAMP Response Element-Binding Protein

CRF Case Report Form

DRRI-2 Deployment Risk and Resilience Inventory-2

FDA Food and Drug Administration

GAD Generalized Anxiety Disorder

GCP Good Clinical Practice

GWBS General Well Being Schedule

HAM-A Hamilton Anxiety Rating Scale

HREC Human Research Ethics Committee

ICH International Council for Harmonisation of Technical Requirements for Pharmaceuticals for Human Use

IMPACT Institute for Mental and Physical Health and Clinical Translation

LEC-5 Life Events Checklist for DSM-5

MAPK Mitogen-activated protein kinases

MINI-7 Mini International Neuropsychiatric Interview-7

MSI-BPD Mclean Screening Instrument for Borderline Personality Disorder

NHMRC National Health and Medical Research Council

OHRO The USAMRDC Office of Human Research Oversight

PCL-5 PTSD Checklist for DSM-5

PGIC Patient Global Impression of Change scale

PHQ-9 Patient Health Questionnaire-9

PHQ-15 Patient Health Questionnaire-15

PKA Protein Kinase A

PI Principal Investigator

PICF Participant Information and Consent Form

PSQI Pittsburgh Sleep Quality Index

PSQI-A PSQI-Addendum for PTSD

PTRS Austin Health Psychological Trauma Recovery Service

PTSD Posttraumatic stress disorder

RACGP The Royal Australian College of General Practitioners

RANZCP The Royal Australian and New Zealand College of Psychiatrists

RCT Randomised Controlled Trial

REDCap Research Electronic Data Capture

SNRI Serotonin and norepinephrine reuptake inhibitors

SSRI Selective serotonin reuptake inhibitor

SSS Social Support Survey

STOP Trial Silexan in the Treatment Of Posttraumatic stress disorder (STOP) trial

SDV Source Data Verification

USAMRDC the U.S Army Medical Research And Development Command

VHA Veterans Health Administration

WHODAS 2.0 WHO Disability Assessment Schedule

# Study synopsis

| Design | Phase 3, 12-week, parallel-arm, randomized, placebo-controlled, double-blind trial. |
| --- | --- |
| Study Centres: | Deakin University, The University of Melbourne, Austin Health, Ramsay Health (Albert Road Clinic) |
| Aim | The aim of this randomised controlled trial (RCT) is to investigate the effectiveness of adjunctive Silexan, compared with placebo, over 12 weeks in improving PTSD symptoms in adults with PTSD. |
| Hypothesis (where relevant) | 1. Adjunctive Silexan will be superior to placebo in improving PTSD symptoms, as measured by the change in CAPS-5 score from baseline to week 12, in adults with PTSD. 2. Adjunctive Silexan will be superior to placebo in inducing remission of PTSD, improving clinical global impression, anxiety symptoms, depressive symptoms, somatic symptoms, sleep quality, quality of life, problematic alcohol use and physiological markers of PTSD in adults with PTSD.   The overall results of the study are expected to support the inclusion of adjunctive Silexan as an evidence-based, guideline-recommended treatment for PTSD. |
| Primary Outcome | The Primary Outcome Measure will be the between-group difference in the change from baseline to week 12 in the total symptom severity score on the Clinician-Administered PTSD Scale for DSM-5 (CAPS-5). |
| Secondary Outcomes | Secondary Outcome Measures will include:   - 1. The between-group difference in the change from baseline to week 12 in measures of anxiety symptoms (HAM-A, GAD-7), depressive symptoms (BDI-II, PHQ-9), somatic symptoms (PHQ-15), sleep quality (PSQI & PSQI-A), problematic alcohol use (AUDIT), functional disability (WHODAS), general well-being (GWBS) and quality of life (AQoL-6D).   2. The between-group difference in the change from baseline to week 16 (while including all available time-points including the off-treatment period) in measures of anxiety symptoms (HAM-A, GAD-7), depressive symptoms (BDI-II, PHQ-9), somatic symptoms (PHQ-15), sleep quality (PSQI & PSQI-A), problematic alcohol use (AUDIT), functional disability (WHODAS), general well-being (GWBS), quality of life (AQoL-6D) and PTSD symptom severity (PCL-5). Additional comparisons for between-group differences from week 12 to week 16 will also be performed.   3. Longitudinal trajectory analysis of measures of sleep (PSQI, PSQI-A, actigraphy), somatic symptoms (PHQ-15), problematic alcohol use (AUDIT), depression (PHQ-9), anxiety (GAD-7), PTSD symptom severity (PCL-5), physiological markers of PTSD, and patient global impression of change (PGIC) in response to treatment. These measures will be assessed at baseline, week 4, week 8 and week 12 of treatment; the PCL-5 and PGIC will additionally be measured at week 2 and week 6 of treatment, and actigraphy and physiological measures will be collected continuously from baseline until week 16. |
| Inclusion criteria | Participants will be included in the study on the basis that they:   1. Are aged 18 years or over. 2. Are fluent in English. 3. Meet DSM-5 criteria for PTSD, irrespective of occupation (e.g first responder, police force, ex-military, civilian), determined using the MINI-7. 4. Have a score on the PCL-5 equal to or over 33. |
| Exclusion criteria | Participants will be excluded if they:   1. Are currently serving in the Australian Defence Force 2. Have a lifetime history of a psychotic or bipolar disorder, or dissociative identity disorder. 3. Have a history of moderate or severe alcohol or other substance use disorder within 3 months of screening. 4. Have active suicidal or homicidal ideation. 5. Have Borderline Personality Disorder. 6. Have an acute or unstable medical illness or other significant medical condition that would make participation in the trial unsafe or inappropriate. 7. Are pregnant, lactating or unwilling to use an acceptable method of contraception. 8. Have commenced a trauma-focussed psychotherapy within 3 months of screening. 9. Have commenced or changed the dose of a psychoactive medication within 4 weeks of screening. 10. Participants will be asked not to initiate psychotherapy or change the dose of psychoactive medications during the course of the study except in clinically urgent circumstances; if this becomes necessary, a decision will be made on a case-by-case basis with regard to retaining the participant or terminating participation. 11. Have a severe acquired brain injury. 12. Are not eligible for public mental health services due to their visa status in Australia or for any other reason. 13. Have any other condition that in the opinion of the research team is likely to make completion of the trial requirements infeasible. 14. Are unable to understand or speak English to the extent necessary to consent and complete the trial (researcher or clinician-determined). |
| Number of Planned Participants | 278 |
| What organisation has overall responsibility for the project? | Deakin University |
| Anticipated start date | June 2024 |
| Anticipated finish date | October 2027 |
| Five keywords/phrases to describe or define the field of research | PTSD, Intervention, Silexan, Lavender Oil, Randomized Placebo-Controlled Trial |
| Risk level | Negligible risk  *☐*   Low risk  *☐*   Greater than low risk  *☒* |

The research will involve the following:

| Active participation of human participants | Yes  *☒*       No  *☐* |
| --- | --- |
| Human biospecimens only | Yes  *☐*       No  *☒* |
| Data associated with human participants | Yes  *☒*      No   *☐* |

# Administrative information

## 3.1 Project team roles and responsibilities

The STOP study is a collaboration between the Institute for Mental and Physical Health and Clinical Translation (IMPACT) at Deakin University, Phoenix Australia: the Centre for Posttraumatic Mental Health (which is affiliated with the University of Melbourne), the Austin Health Psychological Trauma Recovery Service (PTRS) and the Albert Road Clinic. Recruitment will occur through all trial sites, via the posting and circulation of study flyers, and subsequent participant self-referral. Screening and study assessments will be performed by Phoenix Australia staff or other study site staff trained and supervised by Phoenix Australia staff, either on-site at a study site or via teleconference.

| Name | Professor Michael Berk | Institution | Deakin University  Barwon Health |
| --- | --- | --- | --- |
| Position | Alfred Deakin Chair of Psychiatry  Director of the Institute for Mental and Physical Health and Clinical Translation. | Role | Coordinating Principial Investigator (PI) |
| Responsibilities | Professor Berk will have overall responsibility for the trial and will ensure that it conforms with ethical and clinical practice standards. He will be involved in all aspects of trial design and execution as well as the reporting and dissemination of the trial’s findings. | | |

| Name | Professor Richard Kanaan | Institution | Austin Health |
| --- | --- | --- | --- |
| Position | Chair of Psychiatry | Role | Co-PI |
| Responsibilities | Professor Kanaan will be responsible for the management of the trial at the Austin Health Psychological Trauma Recovery Service site. He will be involved in all aspects of trial design and execution as well as the reporting and dissemination of the trial’s findings. | | |

| Name | Dr Rahul Khanna | Institution | Austin Health  Phoenix Australia |
| --- | --- | --- | --- |
| Position | Consultant Psychiatrist  Director of Innovation & Medical Governance; | Role | Co-PI  Community Advisory Board (CAB) clinician representative |
| Responsibilities | Dr Khanna will assist Dr Roebuck with the day-to-day management of the trial at all trial sites. He will also be a clinician representative on the CAB. He will be involved in all aspects of trial design and execution as well as the reporting and dissemination of the trial’s findings | | |

| Name | Dr Greg Roebuck | Institution | Deakin University  Phoenix Australia, The University of Melbourne |
| --- | --- | --- | --- |
| Position | Consultant Psychiatrist | Role | Co-PI |
| Responsibilities | Dr Roebuck will be responsible for all aspects of the day-to-day management of the trial with the assistance of the trial coordinator. | | |

| Name | Professor David Forbes | Institution | Phoenix Australia, The University of Melbourne |
| --- | --- | --- | --- |
| Position | Director | Role | Co-PI |
| Responsibilities | Professor Forbes will, together with Professor O’Donnell, be responsible for the management of the trial at the Phoenix Australia site. He will be involved in all aspects of trial design and execution as well as the reporting and dissemination of the trial’s findings. He will also assist with translation of the trial’s findings into clinical practice. | | |

| Name | Professor Meaghan O’Donnell | Institution | Phoenix Australia, The University of Melbourne |
| --- | --- | --- | --- |
| Position | Professor/Head of Research | Role | Co-PI |
| Responsibilities | Professor O’Donnell will, together with Professor Forbes, be responsible for the management of the trial at the Phoenix Australia site. She will be involved in all aspects of trial design and execution as well as the reporting and dissemination of the trial’s findings. | | |

| Name | Dr Winnie Lau | Institution | Phoenix Australia, The University of Melbourne |
| --- | --- | --- | --- |
| Position | Senior Clinical Research Specialist | Role | Investigator |
| Responsibilities | Dr Lau will assist with oversight of the day-to-day running of activities at Phoenix Australia. Dr Lau will provide input into all phases of the project. | | |

| Name | Dr Mohammadreza Mohebbi | Institution | Deakin University |
| --- | --- | --- | --- |
| Position | Senior Research Fellow | Role | Co-PI |
| Responsibilities | Dr Mohebbi will provide advice regarding the design of the study, will assist with data monitoring and will have overall responsibility for the analysis of trial data. He will supervise the trial statistician. | | |

| Name | Professor Malcolm Hopwood | Institution | Albert Road Clinic |
| --- | --- | --- | --- |
| Position | Ramsay Health Care Professor of Psychiatry and Head of Professorial Psychiatry Unit | Role | Co-PI |
| Responsibilities | Professor Hopwood will be responsible for the management of the trial at the Albert Road Clinic site. He will be involved in all aspects of trial design and execution as well as the reporting and dissemination of the trial’s findings. | | |

| Name | Associate Professor Olivia Dean | Institution | Deakin University |
| --- | --- | --- | --- |
| Position | Director of TRIALS Team | Role | Co-PI |
| Responsibilities | Associate Prof Dean will assist with the management of the trial at the IMPACT site. She will be involved in all aspects of trial design and execution as well as the reporting and dissemination of the trial’s findings | | |

| Name | Mr William Jensen | Institution | N/A |
| --- | --- | --- | --- |
| Position | Australian Army veteran | Role | Community Advisory Board Chair |
| Responsibilities | Mr Jensen is an Australian Army veteran with lived experience of Posttraumatic Stress Disorder (PTSD). He will provide input into the design of the trial and chair a Community Advisory Committee that will include a second consumer with lived experience of PTSD and two clinician representatives. | | |

| Name | Dr Georgia Parkin | Institution | Phoenix Australia, The University of Melbourne |
| --- | --- | --- | --- |
| Position | Research Fellow | Role | Trial Coordinator |
| Responsibilities | Dr. Parkin will manage day-to-day coordination of the trial, under the supervision of PI Roebuck | | |

## 3.2 Conflict of interest

Silexan is manufactured by a German pharmaceutical company, Dr. Willmar Schwabe GmbH & Co KG. The research team have no connection with this company and no financial conflict of interest (or any other conflict of interest) regarding the trial.

## 3.3 Disclosure of intellectual property use

No intellectual property will be used in the conduct of the trial.

## 3.4 Expected duration of study

The study will commence in June 2024, following approval from the University of Melbourne Human Research Ethics Committee (HREC) and the U.S Army Medical Research And Development Command (USAMRDC) Office of Human Research Oversight (OHRO). After approval from the University of Melbourne HREC, approval will be sought from other Australian ethics review bodies under processes for minimising duplication of ethical review in accordance with the National Statement. Recruitment, screening assessment and randomisation will occur between June 2024 and February 2027, providing sufficient time to recruit 278 participants. In the fourth year of the project, we will be continually cleaning and analysing data for publication and initiating discussions with relevant agencies (e.g. the Royal Australian and New Zealand College of Psychiatrists (RANZCP), the Royal Australian College of General Practitioners (RACGP) and the Australian Psychological Society (APS)) regarding clinical translation of the trial’s findings.

***Table 1: Study timeline***

|  | July 2023 – June 2024 | June 2024 – Feb 2027 | | March 2027 – June 2027 | | July 2027 –  Jan 2028 | |
| --- | --- | --- | --- | --- | --- | --- | --- |
| Ethics (HREC, USAMRDC OHRO), staff training, trial registration (ClinicalTrials.gov), study set-up, develop trial (REDCap) database |  |  | |  | |  | |
| Recruitment, screening, & randomisation |  |  | |  | |  | |
| Intervention delivery |  |  | |  |  |  | |
| Follow up assessments |  |  |  |  | |  | |
| Shut down study sites, complete and close out trial administration |  |  | |  | |  |  |
| Data cleaning, analysis, publication, translation |  |  | |  |  |  | |

## 3.5 Assessors

Assessments for the STOP trial will be conducted by a trained research assistant or research fellow blinded as to the treatment condition in a private office room or via teleconference. Researchers conducting assessments will be Phoenix Australia (University of Melbourne), Deakin University, Austin Health or Albert Road Clinic staff members, who are, at a minimum, Honours graduates with training and experience in conducting psychological assessments. All researchers will have up-to-date Good Clinical Practice (GCP) training and will be supervised by a senior research fellow/clinical psychologist with extensive experience in managing and leading PTSD clinical trials (Dr Lau), as well as a consultant psychiatrist experienced in the assessment and treatment of PTSD (PI Roebuck). PI Roebuck will also provide clinical governance to the trial staff. All researchers will receive appropriate induction and training, including local security and risk management policies, as well as where the duress alarms are located, prior to being approved to conduct patient assessments. All assessors, who are not otherwise qualified clinical psychologists or psychiatrists, will have completed the Applied Suicide Intervention Skills Training (ASIST) workshop in suicide first aid accredited by Living Works Australia, Phoenix Australia’s Trauma-Informed Care course and the Department of Veterans’ Affairs ‘Understanding the military experience’ course. Assessors will be trained in administering the Mini-International Neuropsychiatric Interview, version 7.0.2 (MINI-7) and the Clinician-Administered PTSD Scale for DSM-5 (CAPS-5) by a clinical psychologist at Phoenix Australia. Executive oversight of the STOP Trial will be performed by Professor (CI) Michael Berk, who is one of the world’s leading psychiatry researchers and has made several groundbreaking contributions to psychopharmacology. Conduct of the STOP Trial at Phoenix Australia site will be overseen by PI O’Donnell, who is a clinical psychologist and internationally recognised expert in PTSD. PI O’Donnell is Director of Research at Phoenix Australia, and Professor in the Department of Psychiatry at University of Melbourne. Conduct of the STOP Trial at Deakin University will be overseen by CI Berk and PI Dean, who is an Associate Professor at the sponsor site, the Institute for Mental and Physical Health and Clinical Translation (IMPACT) at Deakin University and has a strong track record in the development of novel therapeutics for psychiatric disorders, including multi-centre randomized controlled trials. Conduct of the STOP Trial at Austin Health will be overseen by PI Kanaan, who is a world authority on functional neurological disorder, arguably the first trauma-related mental illness to be recognised. Conduct of the STOP Trial at Albert Road Clinic (Ramsay Health) will be overseen by PI Hopwood, who is a leading psychiatry researcher internationally, with an extensive track record in the areas of PTSD pharmacotherapy, as well as mood and anxiety disorders.

# Introduction and background information

Posttraumatic stress disorder (PTSD) is a debilitating psychiatric disorder characterised by re-experiencing symptoms, avoidance of trauma-related stimuli, deterioration in mood and cognitions and alterations in reactivity and arousal. It is common, with a lifetime prevalence in the general population of the United States of 6.8% (1). Serving military members and veterans are at particularly high risk of developing PTSD. The point prevalence of PTSD in veterans of Operation Enduring Freedom and Operation Iraqi Freedom is approximately 23% (2). PTSD follows a chronic course, with a median time to remission of 14 years (3). It is associated with high levels of disability across all areas of functioning and higher healthcare costs per patient than depression (4, 5). It is a significant factor contributing to veterans’ high rates of disability and their elevated suicide risk (6).

Evidence-based clinical practice guidelines agree that the most effective treatment for PTSD is trauma-focussed psychotherapy (7-9). However, this therapy requires patients to engage with memories of their trauma. Many patients find this process extremely distressing and are unable to tolerate it. Consequently, uptake of trauma-focussed psychotherapy is relatively low despite its status as the ‘gold standard’ treatment for PTSD (10, 11). A study of Veterans Health Administration (VHA) clinics in the United States found that 18 months after extensive training of all VHA therapists in Prolonged Exposure, the trauma-focussed psychotherapy with the largest evidence base, only 12% of VHA patients with PTSD were receiving Prolonged Exposure (12). Trauma-focussed psychotherapy also has high dropout rates (13). Around 35% of veterans disengage from this therapy before its completion (14). In addition to these problems with acceptability and tolerability, there are limitations in the efficacy of trauma-focussed psychotherapy. As many as 50% of patients with PTSD fail to experience a meaningful clinical improvement with therapy (15). Remission occurs in only a minority of cases. Approximately two-thirds of veterans with PTSD who receive trauma-focussed psychotherapy retain their PTSD diagnosis after treatment (16).

Current pharmacological interventions have an established, albeit limited, role in the treatment of PTSD (7-9). The selective serotonin reuptake inhibitors (SSRIs) sertraline, fluoxetine and paroxetine and the serotonin noradrenaline reuptake inhibitor (SNRI) venlafaxine can reduce PTSD symptoms (17, 18). However, the clinical effect of these medications is modest, with meta-analyses finding small effect sizes (17, 18). SSRIs and SNRIs are also associated with unpleasant adverse effects, including headache, nausea, vomiting, insomnia, drowsiness, agitation and sexual dysfunction (19). Sexual dysfunction occurs in up to 80% of patients and is a leading cause of non-adherence (20). There is emerging evidence that the atypical antipsychotics quetiapine and risperidone and the alpha-1 adrenergic antagonist prazosin may also be useful in treating PTSD (17, 18). However, these medications also have small effect sizes, reflecting equally small clinical benefits. Moreover, atypical antipsychotics have long-term cardiometabolic adverse effects that may tilt risk-benefit calculations against their use for many patients with PTSD (21).

The low tolerability and high non-response rates of trauma-focussed psychotherapy and the limited efficacy and significant adverse effects of current PTSD pharmacotherapy underline the urgent need for new pharmacological treatments for PTSD that are effective and well tolerated.

## 4.1 Silexan – pharmacology

Silexan is a standardized lavender oil preparation produced by steam distillation of *Lavandula* *angustifolia* flowers (22). It is available in orally administered immediate-release soft capsules. Its main constituents are the monoterpenoids linalool (36.8%) and linalyl acetate (34.2%) but it also contains in significant concentrations eucalyptol, camphor, borneol, lavandulyl acetate, terpineol and caryophyllene (23, 24). It is manufactured in accordance with the lavender oil monograph in the European Pharmacopoeia, ensuring consistency in its composition (22).

Silexan has a novel pharmacodynamic profile. Like the gabapentinoids, it is a potent inhibitor of voltage-gated calcium channels (24). However, unlike the gabapentinoids, it does not bind to the α2δ subunit of these channels and does not have a preference for P/Q-type channels. Rather, it binds non-selectively to P/Q-type, N-type and T-type channels. The latter two channel subtypes have a complex role in regulating anxiety and T-type channels are involved in fear conditioning mechanisms (25, 26). Silexan also has effects on serotonin-1A receptors, with a position emission tomography study finding that it inhibits binding potential at these receptors in the temporal gyrus, fusiform gyrus, hippocampus, insula and anterior cingulate cortex (27). Inhibition of serorotonin-1A autoreceptors is implicated in the mechanism of action of the SSRIs and the serotonin-1A receptor antagonist pindolol enhances SSRI efficacy (28). Finally, Silexan enhances synaptic plasticity, including by promoting neurite outgrowth and synaptic density (29). These effects are at least partly mediated by activation of protein kinase A (PKA) and mitogen-activated protein kinase (MAPK) signalling pathways, resulting in phosphorylation of the transcription factor, cyclic AMP response element binding protein (CREB). Increased expression and activation of CREB is a common downstream effect of many antidepressant medications (30).

The main constituents of Silexan, linalool and linalyl acetate, have good oral bioavailability (31). Linalyl acetate functions as a prodrug since, once absorbed, it is rapidly hydrolysed to linalool by β-esterases in the liver and other tissues. Evidence from human and animal studies demonstrates that linalool penetrates the blood-brain barrier (31, 32). Linalool is subject to hepatic metabolism and is mostly excreted in the urine (33). The mean terminal elimination half-life of linalool in humans is approximately 4 hours after a single dose and approximately 9 hours after 14 days of once daily administration (34).

## 4.2 Anxiolytic effects of Silexan

Silexan has dose-dependent anxiolytic effects in murine models of anxiety, including the open-field test, elevated zero maze test, elevated plus maze test, social interaction test and novelty-induced suppressed feeding latency test (24, 35). The anxiolytic effects of lavender oil persist in mice with anosmia, suggesting that these effects are not related to the activation of olfactory pathways (36).

Silexan is also effective in the treatment of clinical anxiety disorders, including Generalized Anxiety Disorder (GAD) (37). A 2010 randomized controlled trial (RCT) involving 77 patients with GAD found that treatment with Silexan (80 mg) over 6 weeks was as effective as lorazepam (0.5 mg) in reducing anxiety symptoms, as measured by the Hamilton Anxiety Rating Scale (HAM-A) (38). A subsequent 2014 RCT involving 539 patients with GAD found that treatment with Silexan (80 mg and 160 mg) over 10 weeks was as effective as paroxetine (20 mg) and more effective than placebo in reducing HAM-A score (39). Compared with placebo, Silexan 160 mg had a moderate effect size (Cohen’s *d* = 0.5). Evidence from large RCTs also demonstrates that Silexan is effective in mild or ‘subthreshold’ anxiety disorders, including Anxiety Disorder Not Otherwise Specific (DSM-IV 300.00; ICD-10 F41.9), Mixed Anxiety and Depressive Disorder (ICD-10 F41.2) and Restlessness and Agitation (ICD-10 R45.1) (40).

A recent independent network meta-analysis by Yap and colleagues pooled data from five double-blind RCTs involving a total of 1,320 participants with GAD and subthreshold anxiety disorders (37). It found that Silexan 160 mg was superior to Silexan 80 mg, paroxetine 20 mg, lorazepam 0.5 mg and placebo in reducing anxiety symptoms. Compared to placebo, Silexan 160 mg was associated with a weighted mean difference in HAM-A score of −4.963 (95% confidence interval −7.167 to −2.759). Silexan has been included as a guideline-recommended treatment for GAD in the recently updated World Federation of Societies of Biological Psychiatry guidelines for the treatment of anxiety, obsessive-compulsive and posttraumatic stress disorders (41).

## 4.3 Antidepressant and other effects of Silexan

Silexan also has antidepressant effects in animal models of depression, performing comparably to the tricyclic antidepressant imipramine in the forced swimming test (29). A recent meta-analysis found that Silexan improved comorbid depressive symptoms in patients with GAD and subthreshold anxiety disorders (42). In addition, it has been shown to improve somatic symptoms, sleep and quality of life in these patients(40, 43).

## 4.4 Safety and tolerability of Silexan

Silexan has an excellent safety profile and has been licensed for use in 14 countries, including Australia (40). In the United States, it is available over-the-counter under the proprietary name ‘CalmAid’. Lavender oil preparations, including Silexan, have been granted ‘generally recognised as safe’ status by the Food and Drug Administration (FDA) and are exempt from FDA regulation (44). A copy of the

relevant federal regulation is appended to this document (Appendices 16.2). Note that *lavandula officinalis* is an older name for the *lavandula angustifolia* plant.

Silexan is well tolerated. The only adverse effects identified so far are mild gastrointestinal symptoms, including eructation and breath odour (37). These side effects are uncommon, with a recent meta-analysis finding that participants randomized to Silexan were 6% more likely to experience adverse effects than those randomized to placebo (40). No serious adverse effects due to Silexan have been reported in clinical trials (37). Silexan has no effects on major cytochrome P450 enzymes (including 1A2, 2C9, 2C19, 2D6 and 3A4 enzymes) and does not interact with the oral contraceptive (45, 46). Unlike many anxiolytic agents, it is non-sedating. It does not impair driving performance, does not cause a withdrawal syndrome when ceased and appears to have no abuse potential (47-49).

## 4.5 Rationale for potential utility of Silexan in PTSD

The pathophysiology of PTSD is believed to involve maladaptive changes in the functioning of neural circuits responsible for fear and anxiety responses (50). In particular, it appears to involve fear conditioning in response to severe trauma, leading to an association between trauma-related stimuli and anxiety responses. It also appears to involve impairments in fear extinction, the process by which conditioned fear is unlearned (50). PTSD sufferers display acute anxiety in response to trauma-related cues. They also experience chronic anxiety symptoms, including hypervigilance, irritability, impaired concentration, poor sleep and exaggerated startle response. The foreground presence of anxiety in the pathophysiology and symptomatology of PTSD suggests that Silexan may be useful in the treatment of PTSD.

## 4.6 Pilot evidence of efficacy and tolerability of Silexan in PTSD

An exploratory, open-label trial in 2012 examined the efficacy and tolerability of Silexan in 50 participants with PTSD (ICD-10 F43.1), Neurasthenia (ICD-10 F48.0) or Somatization Disorder (ICD-10 F45.0 or F45.1) (51). Thirty of the participants met criteria for PTSD and 17 had PTSD alone (i.e. without Neurasthenia or Somatization Disorder). All participants received Silexan 80 mg daily for 6 weeks. A pre-post analysis found that participants showed significant improvements in anxiety symptoms, depressive symptoms, quality of life and global distress levels. 44.7%, 51.1%, 57.4%, and 61.7% of participants showed improvements in anxiety symptoms, sleep, depressed mood and restlessness, respectively. A subgroup analysis for participants with PTSD alone found very large effect sizes (Cohen’s *d* ≥ 3) for improvements in anxiety symptoms, depressive symptoms, interpersonal sensitivity and global distress levels (51). However, these findings must be interpreted cautiously given the small size of this subgroup. The trial did not measure PTSD symptoms so the effects of Silexan on symptoms specific to PTSD such as re-experiencing symptoms and avoidance symptoms is unknown.

Data from this trial also suggest that Silexan is well-tolerated in patients with PTSD. Adverse events during the trial were uncommon, with a frequency of 0.02 events per days of treatment with Silexan. Consistent with other Silexan trials, only mild gastrointestinal side effects were reported and there were no serious adverse effects. The rate of dropouts due to adverse effects was 10% (51).

## 4.7 Impact and relevance to military mental health

Serving military members and veterans are at high risk of developing PTSD; the point prevalence of PTSD in veterans of Operation Enduring Freedom and Operation Iraqi Freedom is approximately 23% (2). PTSD is an important contributing factor to the high rates of disability experienced by veterans and their elevated risk of suicide (6). Existing treatments for PTSD have significant limitations and appear to fail veterans in particular (52). Research suggests that these treatments have disappointingly low real-world effectiveness for veterans: a recent large Israeli study found that only 39.4% of veterans who received treatment in a clinic for combat-related PTSD lost their PTSD diagnosis after completing treatment and that even those who lost their diagnosis continued to experience significant mental health difficulties (52). The STOP trial has the potential to provide definitive evidence regarding the effectiveness of Silexan in adult PTSD, thereby supporting the use of an important alternative treatment for serving members and veterans with PTSD.

# Study Objectives

## 5.1 Study aim

The STOP trial aims to investigate the effectiveness of adjunctive Silexan, compared with placebo,

over 12 weeks in improving PTSD symptoms in adults with PTSD.

## 5.2 Study hypotheses

The primary hypothesis is that adjunctive Silexan will be superior to placebo in improving PTSD symptoms, following 12 weeks of daily treatment, in adults with PTSD.

Secondary hypotheses are that adjunctive Silexan will be superior to placebo in:

- 1. Inducing remission of PTSD
  2. Improving clinical global impression
  3. Reducing secondary symptoms of PTSD (including anxiety symptoms, depressive symptoms, somatic symptoms and insomnia)
  4. Improving quality of life
  5. Reducing problematic alcohol use, where present
  6. Reducing physiological markers of PTSD

## 5.3 Study Outcome Measures

The Primary Outcome Measure will be the between-group difference in the change from baseline to week 12 in the total symptom severity score on the CAPS-5 (53).

Secondary Outcome Measures will include:

- - 1. The between-group difference in the change from baseline to week 12 in measures of) anxiety symptoms (HAM-A, GAD-7), depressive symptoms (BDI-II, PHQ-9), somatic symptoms (PHQ-15), sleep quality (PSQI & PSQI-A), problematic alcohol use (AUDIT), functional disability (WHODAS), general well-being (GWBS) and quality of life (AQoL-6D).
    2. The between-group difference in the change from baseline to week 16 (while including all available time-points including the off-treatment period) in measures of anxiety symptoms (HAM-A, GAD-7), depressive symptoms (BDI-II, PHQ-9), somatic symptoms (PHQ-15), sleep quality (PSQI & PSQI-A), problematic alcohol use (AUDIT), functional disability (WHODAS), general well-being (GWBS), quality of life (AQoL-6D) and PTSD symptom severity (PCL-5). Additional comparisons for between-group differences from week 12 to week 16 will be performed.
    3. Longitudinal trajectory analysis of measures of sleep (PSQI&PSQI-A, actigraphy), somatic symptom (PHQ-15), problematic alcohol use (AUDIT), depression (PHQ-9), anxiety (GAD-7) PTSD symptom severity (PTSD Checklist for DSM-5 [PCL-5]), physiology (actigraphy), and patient global impression of change (PGIC) in response to treatment. These measures will be assessed at baseline, week 4, week 8 and week 12 of treatment; the PCL-5 and PGIC will additionally be measured at week 2 and week 6 of treatment, and actigraphy will be collected continuously from baseline until week 16.

## 5.4 Potential benefits and impact

There is an urgent need to develop new treatments for PTSD that are effective and well-tolerated. The STOP trial has the potential to provide definitive evidence of the efficacy of Silexan in adult PTSD. Silexan is safe, well-tolerated, currently available and affordable, facilitating a rapid translation into clinical care. If Silexan is found to be an effective treatment for PTSD, the pool of patients who could potentially benefit from this treatment includes any adults with PTSD.

While we cannot guarantee any direct benefit to participants, the results of the study may help people in the community with similar problems of PTSD in the future. The conduct and results of this study may also advance the academic standing of researchers involved; however, this is not an objective of the study. The researchers strive to advance their knowledge and the knowledge of the PTSD research community regarding this topic and to build additional expertise in the research group, particularly for early- to mid-career staff members.

# Study design and methods

The trial is a phase 3, 12-week, parallel-arm, randomised, placebo-controlled, double-blind trial. Participants will be randomly allocated to receive either (a) Silexan 160 mg daily for 12 weeks in addition to their usual prescribed medications or (b) an identical appearing inert placebo. Participants will be randomly allocated to each of these groups (1:1 ratio; see section 8). The study will employ a 2 (Experimental treatment: Silexan, placebo) x 3 (Assessment point: baseline, week 12, and week 16 [4 weeks post-treatment]) design.


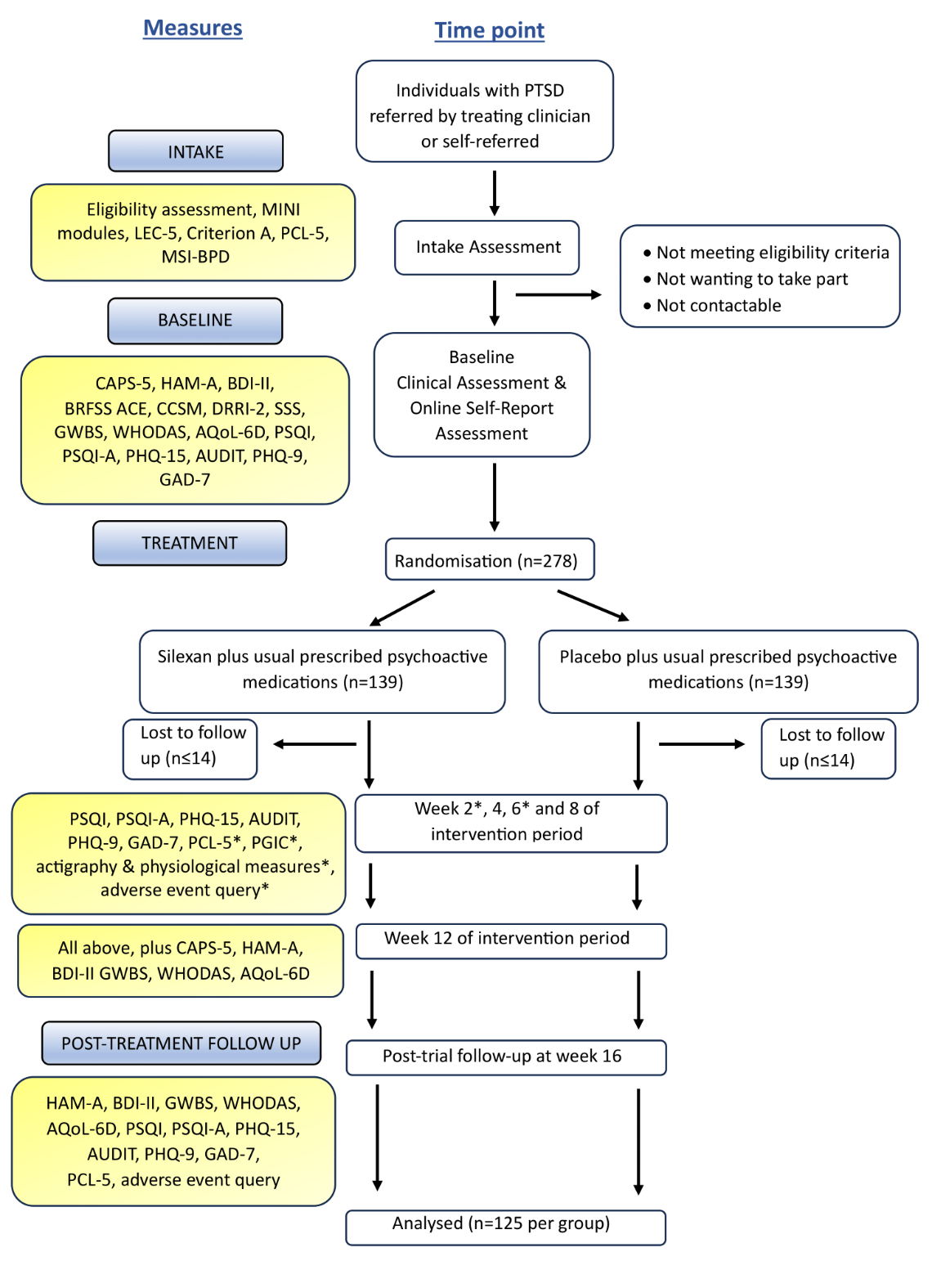


**Figure 1.** Study flow diagram.

## 6.1 Study population

Participants will be adults suffering from PTSD without a history of psychosis, bipolar disorder, Borderline Personality Disorder (BPD) or moderate or severe substance use disorder within the last 3 months.

# Recruitment procedure

## 7.1 Recruitment, referral and advertising study sites

Recruitment and/or advertising will primarily occur through three clinics:

- The Austin Health Psychological Trauma Recovery Service (PTRS).
  - The PTRS is a state-wide service for people with trauma-related mental health conditions. It includes inpatient and outpatient arms and provides care to approximately 350 patients annually.
- The Phoenix Australia Traumatic Stress Clinic
  - The Phoenix Australia Traumatic Stress Clinic is a specialist PTSD outpatient service operated by Phoenix Australia, the National Centre of Excellence in Posttraumatic Mental Health. It is based in Melbourne, Victoria. It receives referrals from a wide range of sources, including General Practitioners, private psychologists, the University of Melbourne health service, Open Arms – Veterans’ & Families Counselling (the national counselling service for military veterans) and welfare organisations for police officers and emergency services workers.
- The Albert Road Clinic (Ramsay Health).
  - The Albert Road Clinic is a private mental health facility. It includes an inpatient unit and outpatient clinics and programs, which together manage approximately 400 patients with PTSD annually.

Treating clinicians at these sites who identify potential participants interested in taking part in the trial will provide those potential participants with a study flyer inclusive of contact details for the intake team, which will be based out of Phoenix Australia. Experienced researchers at Phoenix Australia may also train and supervise staff at other study sites as appropriate. Potential participants will then be required to self-refer to the intake team. Health professionals in Australia will also be made aware of the trial using the email networks for the Royal Australian and New Zealand College of Psychiatrists (RANZCP), the Royal Australian College of General Practitioners (RACGP) and the Australian Psychological Society (APS). In addition, advertisements for the trial will be placed in verified print and electronic media and on verified websites and online social media groups for military veterans (e.g Open Arms – Veterans’ & Families Counselling, Soldier On), police officers, emergency services workers and other high-risk populations; posts in social media groups for military veterans will specify that this study is limited to ex-serving veterans (excluding current serving members). Advertisements will list a contact number and email address that potential participants can contact to discuss the study further.

The study will also utilise Trialfacts to support recruitment efforts. Trialfacts is an Australian company. Its employees work remotely and are based worldwide. It will assist our recruitment efforts using online advertising platforms, as well as its own landing page website for the trial. Potential participants will complete a Trialfacts pre-screening questionnaire (see Trialfacts Promotional Material,) and these answers will then be populated by Trialfacts into a STOP Trial REDcap database. The study sponsor, Deakin University, has contracted Trialfacts for a recruitment plan that guarantees 175 STOP Trial referrals over a 6-month period. All of these referrals will be individuals who reside within Australia. It is estimated that 25 of the 175 referrals will be individuals who are eligible to participate in the trial. All advertising and promotional material utilised by Trialfacts will be generated from content included in the Trialfacts Promotional Material. Potential participants will not be compensated for completing the Trialfacts pre-screening questionnaire; participants recruited through Trialfacts will receive reimbursement in line with study participation and as outlined in section 8.5.

Potential participants who self-refer in response to advertisements, or flyers provided to them by their clinicians, or complete the Trialfacts pre-screening questionnaire and are potentially eligible, will be contacted by telephone for an intake (screening) assessment to assess their eligibility. If they appear to be eligible for the trial, a baseline assessment session will be arranged.

## 7.2 Research project setting

The trial will be conducted in several settings:

1. Phoenix Australia Traumatic Stress Research Clinic – Royal Melbourne Hospital, Royal Park Campus, Parkville, Melbourne.

Trial coordination and study assessments will be conducted in this research clinic using videoconferencing facilities available at this location, as well as in-person office space. Participants can choose to have in-person assessments at this location, another study site location, or, alternatively, assessments via videoconference.

1. Home-based offices of the research team.

Where necessary, trial coordination and study assessments will be conducted out of home-based offices using Phoenix Australia computers and secured networks and Phoenix Australia videoconferencing software (Zoom).

1. Private consulting rooms at other study sites listed in section 7.1

All recruitment and referral sites have consulting rooms that can be used for study assessment sessions and office space that can be used for administrative work related to the study.

1. At participants’ videoconferencing locations.

Participants will receive questionnaires delivered via Research Electronic Data Capture (REDCap) survey from location 1 and/or 2 above, to be completed in their homes. Participants will also be instructed to take the trial medication (Silexan or placebo) daily in their homes. Interview-based assessments with a research assistant may also occur via videoconferencing delivered from locations 1 and/or 2 above, if the participant does not opt for an in-person assessment.

5. Barwon Health Pharmacy

The Barwon Health Pharmacy will operate as the trial pharmacy. The Barwon Health Pharmacy will store trial or placebo medication, which will be picked up and dispensed by blinded study team researchers to randomised participants as appropriate. Trial medication will be prepared - and labelled using randomly generated sequences - by a pharma services company; the code linking these sequences to cohort allocation will be provided to Barwon Health Pharmacy, but not to the study team. This process is therefore organisationally and physically removed from trial coordination and study assessments occurring at Phoenix Australia and other study sites and limits the potential for breaking of study blind.

## Eligibility

### 7.3.1 Initial contact

When potential participants contact the intake team, a research assistant will contact them via telephone and provide a brief overview of the trial. The individual will be asked whether they understand the purpose, methods, demands, potential risks and benefits of the trial as well as its voluntary nature. The research assistant will explain the electronic consent process for intake assessment and that the potential participant will be asked to complete the Life Events Checklist for DSM-5 (LEC-5), Criterion A questionnaire, PCL-5, McLean Screening Instrument for BPD (MSI-BPD) and Mini-International Neuropsychiatric Interview-7 for DSM-5 (MINI-7) Alcohol Disorder and (non-alcohol) Substance Use Disorder questions, following electronic consent. The research assistant will also schedule a time with the potential participant to complete an intake assessment via telephone. This initial contact will take 5-10 minutes.

LEC-5: The **Life Events Checklist for DSM-5** (**LEC-5**) (Appendix 16.5), is a measure of exposure to 16 potentially traumatic events (plus a 17^th^ ‘undefined’/other option) that may result in PTSD (54). Each item is scored on a nominal scale: happened to me, witnessed it, learned about it, part of my job, not sure, and does not apply. The previous iteration, which only differed through the absence of the ‘part of my job’ response option, has demonstrated good convergent and divergent validity, test-retest reliability, and concurrent validity (55).

Criterion A: The **Criterion A** checklist for the DSM-5 Criteria for PTSD (Appendix 16.5), along with the LEC-5 (above) and PCL-5 (below), has been incorporated into a single measurement instrument (56). Inclusion of Criterion A will identify the event in an individual’s life that they consider the worst event, which may then be used as the index event for the PCL-5.

PCL-5: The **PTSD Checklist for DSM-5** (Appendix 16.18) is a 20-item self-report measure that assesses the presence and intensity of each of the 20 DSM-5 PTSD symptoms. Symptoms are scored on a Likert-type scale from 0 (“not at all”) to 4 (“extremely”), with the total score ranging from 0 – 80. At screening, the past-month version of the PCL-5 will be used. The PCL-5 has been shown to have strong reliability and validity (57). A score of ≥ 33 suggests PTSD (56).

MSI-BPD: The **Mclean Screening Instrument for Borderline Personality Disorder** (Appendix 16.4) will be administered to screen potential participants for BPD. The recommended cut-off score of ≥7 will be used to screen for BPD (58).

MINI-7: The **Mini-International Neuropsychiatric Interview-7 for DSM**-5 is a structured diagnostic interview based on DSM-5 criteria (59). MINI-7 modules are comprised of questions requiring Yes/No responses. The Alcohol Disorder, and (non-alcohol) Substance Use Disorder modules will be completed via self-report questionnaire, and the PTSD, Manic Episode, and Any Psychotic Disorder modules will be completed via structured interview.

### 7.3.2 Electronic consent to intake assessment

Initial electronic consent to participate in the intake assessment, as an assessment of study eligibility, will be obtained via DocuSign (21 CFR Part 11 compliant). Following completion of electronic consent, the self-report PCL-5, LEC-5, Criterion A and MSI-BPD questionnaires will be sent to the participant for completion via REDCap survey. If participants are unable to provide electronic consent (for instance, due to lacking access to a computer), they will be mailed hard copy versions of the consent form and intake assessment measures and a self-addressed, pre-paid envelope for sending these documents back to the intake team.

### 7.3.3 Intake assessment

Following completion of the PCL-5, LEC-5, Criterion A, MSI-BPD, and MINI-7 Alcohol and Substance Use modules, as outlined in section 7.3.2, the remainder of the intake assessment will be conducted over the phone and will occur through and be nested within the broader intake process for Phoenix Australia clinical trial participation. All intake processes will be carried out by Phoenix Australia staff, or other study site staff trained and supervised by Phoenix Australia staff, allowing for a standardisation of procedures. An intake worker will call the potential participant to conduct the intake assessment with the purpose of 1) reintroducing the trial, and 2) collecting information to allow assessment of a participant’s eligibility for the trial.

The intake assessment will determine whether potential participants meet the inclusion and exclusion criteria and will utilise the  following MINI-7 modules: PTSD, Manic Episode, Any Psychotic Disorder (60).

### 7.3.4 Inclusion criteria

1. Age 18 years or over.
2. Fluent in English.
3. Meet DSM-5 criteria for PTSD, irrespective of occupation (e.g first responder, police force, ex-military, civilian), determined using the MINI-7.
4. Have a score on the PCL-5 equal to or over 33.

### 7.3.5 Exclusion criteria

1. Are currently serving in the Australian Defence Force
2. Lifetime history of a psychotic or bipolar disorder, or dissociative identity disorder.
3. Moderate or severe alcohol or other substance use disorder within 3 months of screening.
4. Active suicidal or homicidal ideation.
5. Borderline Personality Disorder (BPD).
6. Acute or unstable medical illness or other serious medical condition that would make participation in the trial unsafe or inappropriate.
7. Pregnancy, lactation or unwillingness to use an acceptable method of contraception (see section 7.3.6; required for both males and females who are of reproductive potential and sexually active with partners of the opposite sex) through the duration of participants’ involvement in the study, up to and including week 16). Participants will also be advised not to donate egg or sperm during the study period.
8. Commencement of a trauma-focussed psychotherapy (including Prolonged Exposure, Cognitive Processing Therapy and Eye Movement Desensitisation and Reprocessing) within 3 months of screening.
9. Commencement or change in dose of psychoactive medications within 4 weeks of screening.
10. Participants will be asked not to initiate psychotherapy or change the dose of psychoactive medications during the course of the study except in clinically urgent circumstances; if this becomes necessary, a decision will be made on a case-by-case basis with regard to retaining the participant or terminating participation.
11. Severe acquired brain injury.
12. Individual is not eligible for public mental health services due to their visa status in Australia or for any other reason.
13. Any other condition that in the opinion of the research team is likely to make completion of the trial requirements infeasible.
14. Inability to understand or speak English to the extent necessary to consent and complete the trial (researcher or clinician-determined)

### 7.3.6 Acceptable contraception

Acceptable contraception/birth control methods are defined as: hormonal contraceptives, intrauterine device, or double barrier contraception (ie, male condom and diaphragm, male condom or diaphragm with spermicidal gel or foam). Hormonal contraceptives must have been started at least 2 months prior to the baseline visit.

If it is unclear whether a potential participant is eligible for the trial following the intake assessment, the intake worker will discuss the potential participant with PI Roebuck, who is a consultant psychiatrist experienced in the assessment and treatment of PTSD. PI Roebuck will provide clinical governance to the trial staff. Where necessary, he will escalate the issue to the Phoenix Australia weekly psychiatric oversight meeting. The clinical review team in attendance at this meeting includes the Head of Research and Director of Clinical Services at Phoenix Australia.

If a potential participant is deemed eligible from the intake assessment, they will then be progressed to the baseline assessment stage, where formal written consent to the trial is obtained. They will be contacted (via phone or email when unable to be contacted by phone) by a research assistant or trial coordinator to schedule a baseline assessment.

Individuals who are not eligible to participate as determined by the intake process due to not meeting the inclusion criteria or meeting at least one exclusion criterion will be informed of this (via phone or email when unable to be contacted via phone) by the intake worker or trial coordinator. Individuals will be informed that the trial is not suitable for their current needs and will be provided with information regarding other support options (e.g. referral back to existing treatment providers, GP, Open Arms – Veterans’ & Families Counselling, Beyond Blue). If appropriate, individuals will be advised of other trials at Phoenix Australia that they may be interested in and eligible for and provided alternate study flyers.

The meeting of inclusion/exclusion criteria will be documented on REDCap.

## Written informed consent

Written informed consent to participate in the trial will be obtained at the baseline assessment. Prior to the baseline assessment session, potential participants will be mailed (or emailed according to their preference) a copy of the Participant Information and Consent Form (PICF). They will be encouraged to discuss their participation in the trial with their partners, relatives, friends and treating physicians. At the baseline assessment session, the researcher conducting the session will explain the trial to the participant, read through the PICF with them and answer any questions they have regarding the trial. The researcher conducting the baseline assessment will assess the participant’s capacity to give informed consent to participating in the study, including their understanding of the potential benefits and risks of participating and their apparent ability to weigh this information to reach a reasoned decision about whether to participate.

If the participant wishes to participate in the study and the researcher conducting the baseline assessment considers that they have capacity to give informed consent, the participant and the researcher will both sign the PICF. If the baseline assessment is conducted remotely, this consent process will be conducted via DocuSign and the participant will receive an emailed copy of the completed PICF. If the baseline assessment is conducted in-person, two copies of the PICF will be signed, with the participant retaining one copy and the other copy being retained by the STOP trial study team. If a participant is not comfortable with the use of DocuSign, they will be required to attend the baseline visit in person.

During the consent process, the researcher will confirm that the participant has not commenced a trauma-focussed psychotherapy within 3 months, or commenced or changed the dose of a psychoactive medication within 4 weeks of the intake assessment and will reiterate that they should not commence such a treatment while they are participating in the trial. Participants will also be informed that if they wish to commence any psychological treatments or commence or change the dose of any psychoactive medication during the trial, they need to inform a member of the study team.

At every study visit, participants will be reminded of the study purpose, that participation in the study is voluntary, and that they are free to withdraw from the study at any time.

As per FY22 TBIPHRP CTA requirements, a copy of the HREC-approved consent form will be posted on a publicly available federal website, after the trial is closed to recruitment and no later than 60 days after the last study visit, in accordance with United States federal requirements described in Code of Federal Regulations, Title 32, Part 219 (32 CFR 219).

## Baseline assessment measures

### 7.5.1 Use of videoconference programs

Participants who express interest in attending assessments via videoconference (Zoom) will be emailed a Teleconference Orientation Information Sheet (TOIS) outlining setup instructions for the videoconference, including basic requirements as well as instructions for troubleshooting when utilising Zoom, prior to progressing to the baseline assessment stage. Participants will also be made aware of the inherent cybersecurity risks associated with any online tool (as described in the PICF), and the steps that will be taken to maximise security of their online participation in the study. As per the standard risk management procedure, limits to confidentiality will also be outlined at this stage. Should participants have any concerns regarding equipment or set up of the Zoom videoconference, they will be asked to contact the research assistant prior to the baseline assessment, although potential technical difficulties will also be discussed at the baseline assessment.

Self-report baseline measures will be provided to the participant via email of a REDCap survey link and, if necessary, a QR code provided via videoconference. Generated links and QR codes will link to baseline measure forms within and specific to the participant’s unique REDCap record. Participants will only be able to view the forms associated with that survey link within REDCap, as designated by the research team. Generated links and QR codes do not contain any identifying information. Once forms have been completed by the participant, the generated link or QR code will not provide further access to these forms. Both self-report items and clinician-report items can be completed during the videoconference. Self-report baseline measures may also be completed by the participant outside of the videoconference if time or network connectivity limits completion during the scheduled assessment. Prior to commencing the baseline assessment, the research assistant/trial assessor will discuss with participants what they will do should the videoconference be disconnected. This will include confirming a phone number at which the participant can be reached should the videoconferencing be disconnected and the assessment be diverted to phone.

### 7.5.2 Baseline assessments

The baseline assessment comprises both self-report, and clinician-rated measures, as outlined below. All self-report questionnaires will be delivered using the REDCap survey and can be completed on any personal computer with internet access.

The following information will also be documented at the baseline assessment:

- Questionnaires developed by the investigators collecting information such as:
  - Demographic information, including sex assigned at birth, current gender identity, self-reported height and weight, race, education, socioeconomic status and marital status.
  - Past medical, surgical and psychiatric history, including comorbid mental health disorders.
  - Any pharmaceutical and non-pharmaceutical treatments or medications currently being taken, including over the counter medications, vitamins or herbal remedies, acupuncture or other alternative treatments or planned surgery.
  - Any illicit recreational drug use (with data being stored in a de-identified form and not disclosed without consent, except in circumstances where its disclosure is legally compelled).
  - Trauma-event related information including age at trauma and chronicity of symptoms.

The following clinician-rated measures will be completed at the baseline visit (see section 7.5.4):

CAPS-5: The **Clinician-Administered PTSD Scale for DSM-5** (Appendix 16.22) will be used to assess the severity of PTSD symptoms. The CAPS-5 index event will be the event that the participant indicated was the “worst” event on their LEC-5 and Criterion A questionnaires. The CAPS-5 has good internal consistency, interrater reliability, test-retest reliability and convergent validity in measuring PTSD symptom severity (53). It takes approximately 60-90 minutes to complete and is regarded as the ‘gold standard’ for measuring PTSD symptoms in research settings (61). Wording of the past week version of the CAPS-5 will be modified to query PTSD symptoms in the past two weeks; this two-week version will be used at all CAPS-5 time-points (see Section 7.5.3).

HAM-A: The **Hamilton Anxiety Rating Scale** (Appendix 16.7) consists of 14 items and is the most widely used interview instrument for measurement of anxiety in adults and children (62, 63). The HAM-A measures both psychic anxiety and somatic anxiety (manifestation of physical symptoms or complaints related to anxiety), with each item rated on a scale from 0 (“not present”) to 4 (“very severe”). A score of <17 indicates mild, 18-24 indicates moderate, and 25-30 indicates severe anxiety.

CGI: The **Clinical Global Impression Scale** (Appendix 16.9) is a clinician-rated measure of a patient’s global functioning before and after initiation of a study medication. The CGI comprises three global scores, two – ‘Severity of Illness’ and ‘Global Improvement’ (from baseline) - are rated on a 7-point scale and the third, ‘Efficacy Index’, considers both therapeutic effectiveness and adverse reactions on a scale from 0 (marked improvement and no side-effects) to 4 (unchanged or worse and side effects outweigh the therapeutic effects). There is no total score. Clinicians are required to rate each patient based on their own experience with other patients with the same diagnosis, with or without collateral information (64, 65). At baseline, only the ‘Severity of Illness’ question will be completed.

The following self-report measures will be completed at the baseline visit (see section 7.5.3):

BDI-II: The **Beck Depression Inventory-II** (Appendix 16.6) is the most frequently used self-report measure of depression. It comprises of 21 questions rated on 4-point scales of severity (0-3), with a total score that reflects the level or degree of depression. The BDI has a mean internal consistency (coefficient α) of 0.86 for psychiatric patients and 0.81 for non-psychiatric individuals (66).

CCSM: The **DSM-5 Level 1 Cross-Cutting Symptom Measure** (Appendix 16.8) is a transdiagnostic measure of psychiatric symptoms across different domains. The CCSM is intended to supplement a categorical psychiatric diagnosis, if present, and thereby provide an indication as to the underlying cause of the disorder. It comprises of 23 questions rated on a 5-point scale (0, “none”, to 4, “severe”), across 13 transdiagnostic domains (depression, anger, mania, anxiety, somatic symptoms, suicidal ideation, psychosis, sleep problems, memory, repetitive thoughts and behaviours, dissociation, personality functioning, and substance use). A score of 2 of more is considered a flag for that symptom, with the exception of the substance abuse, suicidal ideation and psychosis items, for which ratings of 1 are also flagged. Test-retest reliability, exclusive of mania, ranges from r=0.64 to 0.97 (67).

WHODAS 2.0: The 12-item self-report version of the **World Health Organization (WHO) Disability Assessment Schedule** (Appendix 16.10) is a measure of functioning and disability across six major life domains due to health conditions. These domains are cognition, mobility, self-care, getting along/social interaction, life activities, and participation in society. All domains were developed with correspondence to International Classification of Functioning, Disability and Health (ICF) items. Items are scored on a 5-point scale from “none” to “extreme or cannot do”. The WHODAS has high internal consistency (Cronbach’s α = 0.86) and test-retest reliability (intraclass correlation of 0.98) (68).

BRFSS ACE: The **Behavioural Risk Factor Surveillance System (BRFSS)** is a population-based system of telephone surveys covering health conditions, risk behaviours and use of preventive services, developed by the Centers for Disease Control and Prevention (69). The **Adverse Childhood Experience (ACE)** module (Appendix 16.15) of the BRFSS was released in 2009 and consists of 11 questions that address exposure to adversity in the time period before the respondent turned 18. The 11 questions span 8 categories: physical abuse, emotional abuse, sexual abuse, household mental illness, household substance use, incarcerated household member, parental separation or divorce, and household domestic violence. These categories may be dichotomised to convey the presence or absence of exposure (70).

PSQI: The **Pittsburgh Sleep Quality Index** (Appendix 16.11) is designed to measure overall sleep quality during the previous month in a clinical population. The PSQI comprises 19 questions across 7 domains or “component” scores, each weighted equally on a 0-3 scale: subjective sleep quality, sleep latency, sleep duration, habitual sleep efficiency, sleep disturbances, use of sleeping medication, and daytime dysfunction. Questions consist of both Likert-type items (rated from “not during the past month” to “three or more times a week”), as well as the documentation of sleep and wake time, time to sleep, and sleep duration. Internal reliability of the PSQI was determined to be α=0.83, test-retest reliability was 0.85. The PSQI showed a sensitivity of 89.6% and specificity of 86.5% in distinguishing good sleepers (healthy controls) from poor sleepers (major depressive disorder and/or a sleep disorder) when using a cut-off score of 5 (71). The PSQI will include the PTSD Addendum (PSQI-A), which are 10 additional questions designed to assess the frequency of disruptive nocturnal behaviours (72).

PHQ-15: The **Patient Health Questionnaire-15** (Appendix 16.12) is a Patient Health Questionnaire module designed to assess the presence of somatic symptoms. The PHQ-15 queries 15 somatic symptoms or symptom clusters that encompass 90% of all physical complaints reported by outpatients. Each item is scored on a scale from 0-2, with total scores ranging from 0-30. The PHQ-15 has good internal consistency (α=0.80-0.87) and test-retest reliability (r=0.65) (73).

DRRI-2: This questionnaire encompasses the deployment-related subscales of the **Deployment Risk and Resilience Inventory-2** (Appendix 16.13), a measure of exposure to potentially traumatic events during military deployments. Questions relate to the deployment environment (scored from 1 [“Almost none of the time”] to 5 [“Almost all of the time”]), combat experiences (scored from 1 [“Never’] to 6 [“Daily or almost daily”], post-battle experiences (scored from 1 [“Never”] to 6 [“Daily or almost daily”]), exposure to nuclear, biological, or chemical agents (scored as either Yes/No/Not Sure), and deployment concerns (scored from 1 [“Strongly disagree”] to 5 [“Strongly Agree”]). The DRRI-2 has high internal consistency reliability (average α= 0.92, all above 0.70), and overall, items showed moderate to strong relationships with PTSD symptom severity (74). It will only be administered to participants with a history of military service.

AQoL-6D: The **Assessment of Quality of Life-6D** scale (Appendix 16.14) is a 20-item, 6-dimensional measure with each item containing between 4 and 6 response levels. The AQoL-6D assesses the following 6 domains of quality of life: Independent Living, Relationships, Mental Health, Coping, Pain and Senses (75). The AQoL-6D may also be used to determine a utility score for assessments of Quality Adjusted Life Years (QALY) (76).

SSS: The **Social Support Survey** (Appendix 16.16) is a multidimensional measure of perceived social support, which addresses areas of emotional, informational support, tangible support, affectionate support, and positive social interaction. Respondents rate each of 19 support items based on how often each is available to them, from 0 (“none of the time”) to 5 (“All of the time”). The SSS has high internal-consistency (α ≥0.91 for all sub-scales) and test-retest reliability (r≥0.72 for all sub-scales) (77).

PHQ-9: The **Patient Health Questionnaire-9** (Appendix 16.17) is a screening measure of depressive symptoms (78). The PHQ-9 asks participants to rate each of the 9 DSM-IV criteria for a depressive disorder from 0 (“not at all”) to 3 (“nearly every day”), with PHQ-9 scores of ≥10 showing 88% sensitivity and 88% specificity for identifying major depression (78).

GWBS: The **General Well Being Schedule** (Appendix 16.19) is an 18-item measure of subjective psychological well-being and distress, developed at the United States National Center of Health Statistics (NCHS) for the U.S Health and Nutrition Examination Survey. The GWBS covers domains (and corresponding sub-scores (79)) of positive well-being, self-control, vitality, anxiety, depression, and general health experienced during the past month. Higher scores reflect better well-being. The GWBS has good test-retest reliability, as well as internal consistency for both sexes (80).

GAD-7: The **Generalized Anxiety Disorder-7** (Appendix 16.20) is a 7-item scale with each item scores on a scale of 0 (“not at all”) to 3 (“nearly every day”) based on symptom presentation in the past two weeks. A score of 0-4 indicates minimal anxiety, 5-9 indicates mild, 10-14 indicates moderate and 15-21 indicates severe anxiety (81).

AUDIT: The **Alcohol Use Disorders Identification Test** (Appendix 16.21) is a 10-item questionnaire developed through a 6-country (Australia, Bulgaria, Kenya, Mexico, Norway, United States) World Health Organization collaborative project, as a screening tool for harmful alcohol consumption. The AUDIT spans the domains of alcohol consumption, drinking behaviour, and alcohol-related problems that may have occurred in the past year, as well as lifetime exposure. Each item is scored on a scale of 0 – 4 (maximum score of 40); a score of 8 was shown to have 92% sensitivity and 94% specificity for hazardous and harmful alcohol use (82). The AUDIT is applicable for both males and females irrespective of ethnicity (83, 84).

#### 7.5.2.2 Verbal consent

With additional optional, verbal consent from the participant, the structured interview portion of the baseline, week 12, and/or week 16 assessments will be audio recorded to maintain inter-rater reliability and quality assurance of the primary outcome measure (CAPS-5); approximately 10% of interviews will be subject to fidelity assessment.

### 7.5.3 Schedule of assessments

Table 2. Schedule of assessments

|  | | Time point (Week, +/- 3 days) | | | | | | | |
| --- | --- | --- | --- | --- | --- | --- | --- | --- | --- |
| Time point (Week): | | T -1  Eligibility Screen (Intake Assessment) | T0  Baseline Assessment | Intervention | | | | | Follow-up/Off-Treatment |
|  |  |  |  | T2 | T4 | T6 | T8 | T12 | T16  Follow-up |
| Clinician/Researcher Administered / Participant Self-reported Measures | | | | | | | | | |
| **Electronic Consent to Screen** | | X |  |  |  |  |  |  |  |
| Inclusion/  Exclusion criteria | | X |  |  |  |  |  |  |  |
| MINI-7 |  | X |  |  |  |  |  |  |  |
| MSI-BPD | | X |  |  |  |  |  |  |  |
| LEC-5 | | X |  |  |  |  |  |  |  |
| Criterion A | | X |  |  |  |  |  |  |  |
| PCL-5 | | X |  |  |  |  |  |  |  |
| **Written Informed Consent** | |  | X |  |  |  |  |  |  |
| CAPS-5 | |  | X |  |  |  |  | X |  |
| HAM-A | |  | X |  |  |  |  | X | X |
| BDI-II | |  | X |  |  |  |  | X | X |
| BRFSS ACE | |  | X |  |  |  |  |  |  |
| CCSM | |  | X |  |  |  |  |  |  |
| Pregnancy test* | |  | X |  |  |  |  |  |  |
| DRRI-2** | |  | X |  |  |  |  |  |  |
| SSS | |  | X |  |  |  |  |  |  |
| GWBS | |  | X |  |  |  |  | X | X |
| WHODAS | |  | X |  |  |  |  | X | X |
| AQoL-6D | |  | X |  |  |  |  | X | X |
| PSQI & PSQI-A | |  | X |  | X |  | X | X | X |
| PHQ-15 | |  | X |  | X |  | X | X | X |
| AUDIT | |  | X |  | X |  | X | X | X |
| PHQ-9 | |  | X |  | X |  | X | X | X |
| GAD-7 | |  | X |  | X |  | X | X | X |
| PCL-5 | |  |  | X | X | X | X | X | X |
| PGIC | |  |  | X | X | X | X | X |  |
| Actigraphy and physiological measures | |  |  | X | X | X | X | X | X |
| Query of Adverse Events*** | |  |  | X | X | X | X | X | X |
| CGI | |  | X |  |  |  |  | X | X |
| Allocation | |  | X |  |  |  |  |  |  |
| Dispensing of trial drug | |  | X |  |  |  |  |  |  |

*for females of child-bearing potential only. If participant is not sexually active or the study team feels that a pregnancy test is not required, it will be omitted.

**for participants with a history of military service

***participants will be queried on adverse events verbally at study visits and via REDCap questionnaire; responses will be reviewed by the study team and followed up if necessary

LEC-5: The **Life Events Checklist for DSM-5** (**LEC-5**), a self-report measure of exposure to potentially traumatic events.

DRRI-2: The deployment-related subscales of the **Deployment Risk and Resilience Inventory-2** (**DRRI-2**), a self-report measure of exposure to potentially traumatic events during military deployments.

BRFSS ACE: The **Behavioural Risk Factor Surveillance Survey Adverse Childhood Experience Module** (BRFSS ACE),

a self-report measure of exposure to adverse childhood experiences.

MINI-7: The **MINI-7** PTSD, Manic Episode, Any Psychotic Disorder, Alcohol Use Disorder and (non-alcohol) Substance Use Disorder modules.

CCSM: The **DSM-5 Level 1 Cross-Cutting Symptom Measure** (**CCSM**), a self-report measure of psychiatric symptoms in different domains.

GWBS: The **General Well Being Schedule** (**GWBS**), a self-report measure of subjective wellbeing and distress.

SSS: The **Social Support Survey** (**SSS**), a self-report measure of social support.

PCL-5: The **PTSD Checklist for DSM-5** (**PCL-5**), a self-report measure of PTSD symptoms.

CGI/PGIC: The **Clinical Global Impression** (**CGI**) and **Patient Global Impression of Change** (**PGIC**) scales, clinician- and patient-rated measures of overall improvement.

HAM-A: The Hamilton Anxiety Rating Scale (**HAM-A)**, a clinician-rated measure of anxiety symptoms.

PHQ-9: The **Patient Health Questionnaire-9** (**PHQ-9**), a self-report screening measure of depressive symptoms

GAD-7: The **Generalized Anxiety Disorder-7** (**GAD-7**), a self-report screening measure of GAD symptoms.

BDI-II: The **Beck Depression Inventory-II (BDI-II),** a self-report measure of depressive symptoms.

PSQI: The **Pittsburgh Sleep Quality Index** (**PSQI**), a self-report measure of sleep quality.

PSQI-A: A **PTSD Addendum** to the PSQI, designed to assess disruptive nocturnal behaviors.

PHQ-15: The **Patient Health Questionnaire-15** (**PHQ-15**), a self-report measure of somatic symptoms.

AUDIT: The **Alcohol Use Disorders Identification Test** (**AUDIT**), a self-report screening measure of problematic alcohol use.

WHODAS 2.0: The 12-item self-report version of the **WHO Disability Assessment Schedule** (**WHODAS 2.0**), a measure of functioning.

AQoL-6D: The **Assessment of Quality of Life-6D** (**AQoL-6D**) scale, a self-report measure of health-related quality of life.

# Randomisation, blinding and intervention

## 8.1 Randomisation and blinding

Randomisation will take place after the eligibility of participants to take part in the trial has been confirmed, and they have had their baseline assessment. Participants will be randomised in a 1:1 ratio to the Silexan or placebo arm. The randomisation list will be created using permutated random-block randomisation (block sizes of 2, 4, and 6). To ensure blinding and allocation concealment, random block sizes will be used and selected block sizes will be concealed from the research team. Randomisation will be stratified by site. The generation of the allocation sequence using randomisation software will be conducted by an independent statistician, who will also retain the random allocation list.

Participants in the placebo arm will receive capsules containing an inert placebo in addition to their usual medications. Blinding will be maintained by ensuring that the packaging, appearance and colour of the Silexan (treatment) and placebo capsules are identical. In addition, the lavender odour of the Silexan capsules when opened will be matched by adding 0.08 mg of lavender oil (0.05% of treatment dose) to each placebo capsule (39). Participants will be instructed to swallow capsules unchewed. Blinding will also be maintained by identifying participants using anonymous participant ID numbers and not giving the research team access to the randomisation code.

Treatment unblinding will only occur in a medical emergency where the appropriate management of the participant necessitates knowledge of the treatment allocation (see section 10.4). All cases of emergency unblinding will be thoroughly documented and reported to the data safety monitoring board (DSMB). After all participants have completed the study, participants will be notified which arm of the study they took part in.

## 8.2 Intervention treatment

Silexan is a lavender oil preparation produced by steam distillation of *Lavandula* *angustifolia* flowers and has been licensed for use in 14 countries, including Australia. It is manufactured in accordance with the lavender oil monograph in the European Pharmacopoeia, ensuring consistency in its composition (22). It is stable at ambient temperatures and does not require any special handling or storage procedures.

In Australia, Silexan is available as an over-the-counter complementary oral medicine under the proprietary name ‘Seremind’. It is a ‘listed medicine’ in the Australian Register of Therapeutic Goods (ARTG). Permitted indications for which it can be marketed include mild anxiety symptoms and sleep

problems. The public summary of its ARTG entry is appended to this document (Appendix 16.1). Seremind will be purchased for this study and repackaged to make it indistinguishable from the placebo packaging.

Participants in the Silexan arm will receive Silexan 160 mg daily in the morning for 12 weeks in addition to their usual prescribed psychoactive medications. Participants will also be followed up at 4 weeks post-treatment (off-treatment period). No modifications of allocated interventions will be made for any trial participants; if appropriate (i.e following the emergence of adverse events) participants will be withdrawn from the intervention (see section 9).

### 8.2.1 Monitoring treatment adherence

Participants will be asked to return medication packs at in-person visits or via pre-paid envelope to allow capsule counts to monitor adherence.

## 8.3 Intervention assessments

The AQoL-6D, GWBS and WHODAS will be collected at baseline, week 12 of the intervention period, as well as at week 16 (+4 weeks post-trial).

The PSQI, PHQ-15, AUDIT, PHQ-9 and GAD-7 will be collected at baseline, week 4, 8 and 12 of the intervention period, as well as at week 16.

The PGIC, PCL-5 and Query of Adverse Event will be collected at week 2, 4, 6, 8 and 12 of the intervention period, as well as at week 16 (Table 2; see section 7.5 for assessment overviews).

The CGI will be collected at week 12 of the intervention, as well as at week 16.

The CGI and PGIC are clinician- and patient-rated measures of overall improvement:

CGI: The **Clinical Global Impression Scale** (Appendix 16.9) is a clinician-rated measure of a patient’s global functioning before and after initiation of a study medication. The CGI comprises three global scores, two – ‘Severity of Illness’ and ‘Global Improvement’ (from baseline) - are rated on a 7-point scale and the third, ‘Efficacy Index’, considers both therapeutic effectiveness and adverse reactions on a scale from 0 (marked improvement and no side-effects) to 4 (unchanged or worse and side effects outweigh the therapeutic effects). There is no total score. Clinicians are required to rate each patient based on their own experience with other patients with the same diagnosis, with or without collateral information (64, 65).

PGIC: The **Patient Global Impression of Change Scale** (Appendix 16.9) asks participants to rate the change (if any) that they have experienced since starting treatment, on a scale of 1 (“No change”) to 7 (“A great deal better and a considerable improvement that has made all the difference”).

### 8.3.1 Actigraphy and physiological measures

Participants will be asked to wear actigraphy watches for the duration of the study period (16 weeks). The actigraphy watch used will be the Actigraph LEAP (https://theactigraph.com/actigraph-leap), which is US FDA 510(k) cleared for the measurement of activity, sleep and mobility. Actigraph complies with the following regulations and standards (<https://theactigraph.com/compliance>):

ISO 13485:2016 Medical Devices - QMS

European Union Medical Device Regulation (EU MDR)

Health Canada Medical Devices Regulations (CMDR)

US FDA's Quality System Regulations (QSRs)

US HIPAA

Australia Therapeutic Goods (Medical Devices)

MDSAP ISO 13485:2016 certified

Data collected from the LEAP will include continuous measures of sleep time and efficiency, heart rate and heart rate variability, blood pressure, oxygen saturation/SpO2, body temperature, breathing/respiratory rate, activity level, gait and step count and energy expenditure, across the 16-week study period. The watches will be given to participants undergoing in-person baseline assessment sessions during these sessions. For participants undergoing baseline assessment sessions via videoconference, the watches will be mailed to them.

Data from the LEAP watches will be uploaded, stored and managed using ActiGraph’s secure, permissions-based CentrePoint cloud software program (<https://theactigraph.com/centrepoint>), which utilises Microsoft Azure and Amazon Web Services cloud platforming services and meets ISO 27001, NIST, HIPAA, FedRAMP, SOC 1, and SOC 2 standards and regulations. Participants will be instructed to download the Actigraph CentrePoint Connect phone application upon receiving the LEAP watch; data from the LEAP watch will be automatically uploaded to the CentrePoint program via bluetooth at regular intervals. Watches and data will be associated only with de-identified participant IDs. Only the study team will have access to the CentrePoint program and this data; data will additionally be downloaded as .csv files onto secure servers in study-specific folders at Phoenix Australia at regular intervals. Watches will be returned to the study team at the end of the study period, either in-person or using pre-paid envelopes, along with the return of used trial medication bottles. Once watches are returned, they will undergo a data wipe/reset.

The collection of actigraphy data will complement the PSQI by providing an objective measure of sleep quality. Previous research suggests that self-reported sleep quality may differ significantly from objective actigraphic measures of sleep quality (85). Research also indicates that PTSD is associated with physiological changes, including increased resting heart rate and blood pressure (86). The collection of physiological measures will assist the research team to determine whether any treatment effect of Silexan is associated with changes or improvements in physiological markers of PTSD.

#### 8.3.1.1 Risks associated with actigraphy use

The ActiGraph LEAP devices are US FDA 510(k) approved and there are no anticipated or foreseen risks associated with use. However, participants will be advised that they can remove the watch at any time if it is causing them discomfort or there is any other reason why they do not want to wear it. The watch is water resistant to a rating of IPX7. However, participants will be instructed to remove it before showering or swimming or doing any other activity that they believe may damage the watch.

## 8.4 GP Letter

By consenting to participating in this trial, a participant also consents to providing the researchers with contact details of their regular treating General Practitioner (GP), who will be contacted regarding their participation in the form of a letter. GP letters will be sent directly to the treating GP via REDCap or email; a copy of the letter will also be saved on the REDCap server. GP letters will follow a standard template.

## 8.5 Participant remuneration

Participants will be reimbursed for expenses associated with their participation in the study, including expenses associated with parking, transport, utilising a computer (for participants undergoing study visits via videoconference) and wearing and recharging the actigraphy watches. Reimbursement will be $50 AUD per visit (baseline, week 2, week 4, week 6, week 8, week 12, week 16). This reimbursement may take the form of a prepaid Visa or Mastercard gift card. For participants who complete their assessments remotely, gift cards may be provided via email or mail, as preferred by the participant. The provision of this compensation is outlined in the STOP trial PICF.

# 9. Participant withdrawal

Participants are free to withdraw from the study at any time upon their request. Withdrawing from the study will not affect their access to standard healthcare interventions or their relationship with any health service or affiliated health care professionals. When a participant withdraws from the study, the reasons for withdrawal (if provided) will be recorded by the investigator in the REDCap database and in the participant’s file.

Withdrawal from the trial will occur in the following circumstances: (1) non-adherence to the trial medication for seven consecutive days, (2) pregnancy or cessation of effective contraception, (3) withdrawal of consent, (4) emergence of serious adverse effects (in the view of the research team or participant) (5) emergence of new information about Silexan that places withdrawal in the participant’s best interest. No subsequent outcome data will be obtained from withdrawn participants. In addition to the reason for withdrawal, the stage at which this occurs will also be recorded on REDCap: Stage 1: following informed consent but prior to receiving the treatment or placebo medication; Stage 2: during the intervention period; Stage 3: after the intervention period but before the completion of the follow-up appointment.

The STOP trial PICF outlines that if a participant withdraws from the study, the data provided by the participant up until their withdrawal may still be used for the study. The participant is informed that they may let the study team know if they do not wish for their data to be used. However, the STOP trial PICF also states that once de-identified participant data is added to the NDA (see section 11.3), the study researchers cannot remove study data that was shared before they were notified that a participant changed their mind.

As outlined in section 11.2.2, an electronic document that links individuals with their unique code identifier, will be stored separately on the Phoenix Australia server. The study specific storage drive will have restricted access and only those who have been identified as the study team will be able to access it. Only the study team will be able to link the study ID and identifying information. Therefore, the study team will also have the ability to exclude withdrawn data from the final dataset.

The recruitment target (n=278) accounts for a 10% drop-out rate. Additional replacement participants will not be sought.

## 9.1 Determination of loss-to-follow-up

Participants who fail to return for study assessments will be contacted by the research team and requested to follow the trial protocol. The participant will receive up to two documented phone calls and up to two letters/emails on different days per study visit before they are considered lost to follow-up.

As each study visit time-point has a window of +/- 3 days, study assessments will first be scheduled for -3 days from the due date. Participants will then receive reminder phone calls or letters/emails (e.g) -1 day (phone call), +1 day (email/letter) and +3 days (phone call or email/letter as appropriate) from the due date. Slight variations in timing may occur, governed by participant schedule (e.g if a participant states they will be away for two days) and availability if attending a visit in-person.

# Adverse events and risks

## 10.1 Definitions

*Adverse Event (AE):* is the development of an undesirable medical condition or the deterioration of a pre-existing medical condition following or during exposure to a pharmaceutical product, whether or not considered causally related to the product. An undesirable medical condition can be symptoms (e.g., nausea, chest pain), signs (e.g., tachycardia, enlarged liver) or the abnormal results of an investigation (e.g., laboratory findings, electrocardiogram). In clinical studies, an AE can include an undesirable medical condition occurring at any time, including washout periods, even if no study treatment has been administered.

*Serious Adverse Event (SAE):* is an AE occurring during the treatment phase (i.e. baseline to week 12) of the trial that fulfils one or more of the following criteria:

- results in death
- is immediately life-threatening
- requires in-patient hospitalisation or prolongation of existing hospitalisation
- results in persistent or significant disability or incapacity
- is a congenital abnormality or birth defect
- is an important medical event that may jeopardise the patient or may require medical intervention to prevent one of the outcomes listed above.

Please note that the following events associated with hospitalisation will NOT be considered an SAE:

- Hospitalisation for evaluation or treatment of a pre-existing condition as long as the condition is the reason for the hospitalisation AND provided that the condition has not worsened in severity since commencement on study treatment.
- Elective or pre-planned treatment of a pre-existing condition that has not worsened during the course of receiving study treatment.

*Significant Safety Issue (SSI)*: is a safety issue that could adversely affect the safety of participants or materially impact on the continued ethical acceptability or conduct of the trial. The Therapeutic Goods Administration (TGA), University of Melbourne HREC and site investigators will be notified within 15 days of the sponsor being made aware of an SSI.

*Suspected Unexpected Serious Adverse Reaction (SUSAR)*: is an adverse reaction that is both serious and unexpected. SUSARs will be reported to the TGA within 7 days for fatal or life threatening Australian SUSARS and no later than 15 days for all other SUSARs.

*Unexpected Adverse Reaction*: is an adverse reaction the nature or severity of which is not consistent with the Reference Safety Information (RSI) (Product Information) for Silexan.

*Urgent Safety Measure*: is a measure required to be taken in order to eliminate an immediate hazard to a participant’s health or safety. This safety measure may be implemented before seeking approval from HRECs or institutions; notification will occur within 72 hours of becoming aware of the event.

### 10.1.1 Causality

The Site Investigator (or person delegated by the site investigator) will assess and monitor each adverse event and assess its relationship, if any, to the study treatment. Causality will be assessed using the following categories: none, remote, possible, probable and highly probable.

The relationship of the adverse event to the investigational product will be based on the following criteria:

- **None**: The adverse event is definitely not associated with the investigational product administered.
- **Remote**: The temporal association is such that the investigational product is not likely to have had an association with the adverse event.
- **Possible:** This causal relationship is assigned when the adverse event:
  - Follows a reasonable temporal sequence from the administration of the investigational product, but
  - Could have been produced by the participant’s clinical state or other modes of therapy administered to the participant.
- **Probable:** This causal relationship is assigned when the adverse event:
  - follows a reasonable temporal sequence from the administration of the investigational product;
- Abates upon discontinuation of the investigational product; and
- Cannot be reasonably explained by known characteristics of the participant’s clinical state.
- **Highly Probable**: This relationship is assigned when the adverse event:
  - follows a reasonable temporal sequence from the administration of the investigational product;
  - abates upon discontinuation of the investigational product; and
  - is confirmed by the re-appearance of the adverse event on repeat exposure.

Possible relationship of an adverse event to the study treatment, any other disease present at the time of the adverse event and any concomitant drug treatment, should be recorded on the adverse event form.

The degree of certainty with which the relationship of an adverse experience is linked to drug treatment will be determined by how well the experience can be understood in terms of:

- The known pharmacological properties of the drug.
- The course and nature of the adverse event.
- The current health status of the participant.
- Knowledge of similar adverse events for the study drug or other drugs of that class.
- The relationship between the time of administration or whether the adverse event can be reproduced on re-challenge of the study drug.

## 10.2 Eliciting adverse event information

Safety events will be recorded from the time the participant signs the informed consent form until the final follow up visit at week 16. Participants will be asked about the possibility of adverse events at each treatment visit (2, 4, 6, 8 and 12 weeks of treatment, as well as at the 4 weeks following the cessation of treatment [week 16]).

## 10.3 Safety reporting

### 10.3.1 Reporting of AEs

Data regarding AEs will be collected from the baseline assessment session until the end of the follow-up period at week 16. At each study visit, participants will be asked if they have had any health problems since the previous visit. All AEs will be recorded appropriately, whether or not they are considered related to the trial medication. This will include AEs spontaneously reported by the participant and/or observed by the staff as well as AEs reported in response to a direct question e.g. ‘Have you had any health problems since your last visit?’, ‘Have any other notable events occurred that you feel have affected your mental or physical wellbeing?’, ‘Have you had any trouble or felt any distress while completing the study questionnaires?’ If a participant has been provided with an ActiGraph LEAP device, they will be asked ‘Have you had any difficulty wearing the activity tracker?’. Participants will also be able to contact the research team via telephone or email in between study visits to report AEs.

Detailed records of all AEs will be documented in REDCap. The description of each AE on the REDCap form will include:

- a description of the event;
- the start and stop date;
- whether the AE fulfils the criteria for a SAE or not;
- an assessment of the causal relationship between the event and the trial medication;
- any treatment provided for the AE;
- the intensity of the AE;
- any action taken regarding the trial medication (if applicable); and
- whether the AE caused the participant to withdraw from the trial.

The week 16 follow-up visit occurs 4 weeks after completion of the intervention period and will be the last time point at which an AE is followed up. If an AE persists until week 16, the participant will be advised that they should see their own treating GP regarding the AE. Further follow-up of an AE can occur at the discretion of the treating physician or research team.

### 10.3.2 Reporting of SAEs

Reporting of SAEs to regulatory authorities will be done by the research team in compliance with

NHMRC guidelines and regulatory requirements. All SAEs will be reported to the appropriate

regulatory agencies within 24 hours of the research team becoming aware of them. SAEs will also be

reported to the University of Melbourne HREC. The trial coordinator and coordinating PI (PI Berk) or their nominated delegates will be responsible for reporting the SAEs to the appropriate authorities.

Adverse effects reported by participants will be assessed and reviewed regularly by the research team and an independent Data Safety Monitoring Board (DSMB) (section 13.2). As noted, there are no known serious adverse effects of Silexan. However, in the event that a SAE occurs, participants will be assisted to seek appropriate emergency medical help through the Australian public health system. The coordinating PI (PI Berk) will be responsible for determining whether unblinding is required on safety grounds. If unblinding is required, a report will also be sent to the Therapeutic Goods Administration.

For less serious AEs, participants will be advised to see their current treating GP.

## 10.4 Risks

### 10.4.1 Mental state

During the intake assessment and baseline assessment session, participants will be asked about previous traumatic experiences that they have had. At all study sessions, they will be asked about their PTSD symptoms and general functioning. There is a risk that participants will become emotionally distressed by these questions. In many cases, this distress may be mild and short-lived.

If a participant experiences distress during a study session, the researcher conducting the session will provide first-line support to them in accordance with the Phoenix Australia distress and risk management protocol. Strategies for managing distress outlined in this protocol include: (1) reassurance and normalising the experience of distress, (2) encouraging the participant to utilise their usual coping strategies, (3) exploring the social supports available to the participant (such as family members and friends), (4) teaching the participant to use basic breathing relaxation techniques, (5) teaching the participant to use basic sensory grounding techniques, (6) encouraging the participant to see their usual health practitioners, and (7) seeking support and advice regarding the situation from senior clinicians at Phoenix Australia. If a participant contacts the research team and reports distress outside of a study session, the risk management protocol will also be followed. Where relevant such as in circumstances of doubt, clinical need or risk, escalation to Dr Roebuck will be conducted. Dr Roebuck, as a consultant psychiatrist and PI, is the first point of call for these issues. The distress and risk management protocol also includes a clinical escalation risk pathway, and a list of senior clinicians and psychiatric registrars and consultants who can be contacted if Dr Roebuck is not available as first point of call.

If a participant experiences prolonged distress and requires further psychiatric assessment or treatment, they will be referred by the research team to the appropriate public mental health service.

The research team will also ask about current living arrangements and request the contact details for a next of kin as part of the intake process, and a current treating General Practitioner as part of the baseline assessment process; the intake assessment consent form (section 7.3.3) and trial consent form (section 7.4) outline that research team will utilise these contacts if there are concerns for the participant’s immediate safety.

In addition, participants will be provided with a 24/7 contact number for a study clinician, who they may call if experiencing severe distress.

### 10.4.2 Management of suicidality

All participants will be required to nominate a current treating GP on their enrolment in the study and will be advised that their GP may be contacted if the research team feel this is appropriate. If a participant reports suicidal ideation with a specific plan or intent (including selecting “I would like to kill myself” or “I would kill myself if I had the chance” on the BDI-II), the researcher will strongly advise the participant to see their current treating GP and will also attempt to contact the treating GP directly. Should the researcher have significant concerns for the immediate safety of the participant (e.g. severe suicidality with immediate risk or a recent, unreported suicide attempt), the researcher will seek advice from PI Roebuck and will take appropriate steps to ensure participant safety (including informing the treating GP and assisting the participant to access appropriate care, for example by referring the person to the emergency department). The researcher will follow the distress and risk management protocol, developed by Phoenix Australia, which includes a clinical escalation risk pathway, and a list of senior clinicians and psychiatric registrars and consultants who can be contacted if PI Roebuck is not available as first point of call. All participants who report suicidal ideation during the trial will be provided with a wallet insert containing national helplines. Additionally, all researchers will have undertaken the Applied Suicide Intervention Skills Training (ASIST) workshop in suicide first aid accredited by Living Works Australia if they are completing trial assessments. A follow up phone call will also be made to the participant from a clinician within 48 hours to follow up on the participant’s response (for score above 5), so that additional risk assessment and management can be undertaken.

### 10.4.3 Treatment-related direct effect

Silexan is available in Australia as an over-the-counter medication. In the United States, lavender oil preparations, including Silexan, have been granted ‘generally recognised as safe’ status by the Food and Drug Administration (FDA) and are exempt from FDA regulation (87) (see section 8.2).

To date, identified adverse side effects of Silexan are limited to mild gastrointestinal symptoms (37). A recent meta-analysis suggested participants taking Silexan are 6% more likely to experience these symptoms compared to placebo (40):

- Eructation (belching or burping)
- Breath odour
- Feeling sick (nausea)
- Dyspepsia/Indigestion (upper abdominal discomfort)

In addition, dermatitis (swelling and skin irritation) is a rare side effect of topically applied lavender oil preparations (88).

There are no known serious side effects of Silexan. However, it is possible that there are serious side effects that we do not know about.

### 10.4.4 Treatment-related indirect effect

Animal studies of Silexan have found no evidence of teratogenicity or adverse effects on fertility, pregnancy or lactation. *Lavandula angustifolia* is commonly taken by women during pregnancy (89). However, the effects of Silexan on the unborn child and on the newborn baby are not known. Because of this, it is important that trial participants are not pregnant or breast-feeding and do not become pregnant during the research project. Female participants with childbearing potential will be required to do a pregnancy test in the period between the initial call and the start of the intervention. Both male and female participants will be strongly encouraged to use effective contraception during and for a period of one month after completion of the research project.

Female participants who become pregnant during the trial will be withdrawn immediately and advised on further medical attention as required. Male participants will be advised to inform the trial clinician if they father a child while participating in the research project. The trial clinician will advise on medical attention for the partner of the male participant if necessary.

### 10.4.5 Legal risk

Participants are asked about illicit recreational drug use at all study visits. While this information will be kept confidential, there is a risk that legal authorities could compel the disclosure of this information and that it could be used against participants in legal proceedings. This risk is outlined in the Participant Information and Consent Form.

### 10.4.6 Management of pregnancy

In the event that a female participant becomes pregnant, treatment with the trial medication will immediately be terminated. Pregnancy itself is not regarded as a SAE unless there is a suspicion that the trial medication may have interfered with the effectiveness of a contraceptive medication. However, the outcome of all pregnancies (spontaneous miscarriage, elective termination, normal birth or congenital abnormality) will be followed up and documented. All reports of congenital abnormalities/birth defects are SAEs. Spontaneous miscarriages will also be reported and constitute SAEs. Elective terminations of pregnancy without complications will not be treated as AEs.

## 10.5 Benefits

There are no guaranteed direct benefits for participants from participating in the trial. However, if the trial finds that Silexan is an effective treatment for PTSD, this will benefit the overall population of people suffering from PTSD by providing them with an additional evidence-based treatment for their PTSD.

The conduct and results of this study may also advance the academic standing of researchers involved; however, this is not an objective of the study. The researchers strive to advance their knowledge and the knowledge of the PTSD research community on this topic and build additional expertise in the research group, particularly for early- to mid-career staff members.

# Data collection

## 11.1 Data capture methods

Data will be captured and stored in a REDCap database. REDCap is a secure web-based data management tool used for building and managing online surveys and databases. It complies with the requirements of the United States Health Insurance Portability and Accountability Act and is widely used for clinical research. It was developed by a multi-institutional consortium initiated at Vanderbilt University.

Raw participant data will be de-identified by associating each participant with a unique code identifier. Any participant who undergoes the screening phase will be automatically assigned a REDCap ID number that will be recorded in the intake assessment database. Participants who attend the baseline assessment and sign the informed consent form (are enrolled), will be given another unique code identifier through REDCap that will be referred to for the remainder of their participation in the trial. A database on the Phoenix Australia server will contain the information which links the individual with their unique code identifier.

Utilising REDCap, participants will provide data during the intake assessment, at the baseline assessment, and 2, 4, 6, 8, 12 and 16- weeks post-randomisation (+/- 3 days) (see Figure 1 and Table 2). Electronic data will be collected and stored online in a de-identified form using the REDCap system. Data will be entered directly into a de-identified electronic case report form on REDCap by participants, and either directly or transcribed from paper source documents by the research team, using computers and tablets. REDCap variables will include range checks for data values where appropriate. The computers will be secured by password access. On one instrument, the participant’s name and email will be entered to allow automated survey invites to be sent. These fields will be coded as ‘containing identifying information’, which prevents the data from being exported; this is a feature of the REDCap database.

Any relevant paper measures will be entered into the REDCap database by the study team (as noted on the delegation log) within a week of data collection. Only study team members will have access to the study drive and the REDCap database via their own login details. Regular data quality checks, such as automatic range checks, to identify data that appear inconsistent, incomplete, or inaccurate, will be performed.

The investigators will maintain adequate case histories of study participants, including accurate CRFs, and source documentation.

All paper source documents will be completed in a neat, legible manner to ensure accurate interpretation of data. Black or blue ink is required to ensure clarity or reproduced copies. When making changes or corrections, the original entry will be crossed out with a single line, initialled and dated.

### 11.1.1 Actigraph data collection

Data from the LEAP watches will be uploaded, stored and managed using ActiGraph’s secure, permissions-based CentrePoint cloud software program (<https://theactigraph.com/centrepoint>), which utilises Microsoft Azure and Amazon Web Services cloud platforming services and meets ISO 27001, NIST, HIPAA, FedRAMP, SOC 1, and SOC 2 standards and regulations. Participants will be instructed to download the Actigraph CentrePoint phone application upon receiving the LEAP watch; data from the LEAP watch will be automatically uploaded to the CentrePoint program via bluetooth at regular intervals. Watches and data will be associated only with de-identified participant IDs. Only the study team will have access to the CentrePoint program and this data; data will additionally be downloaded as .csv files onto secure servers in study-specific folders at Phoenix Australia at regular intervals.

## 11.2 Data storage

### 11.2.1 Electronic data storage (REDCap)

All data will be stored and managed in accordance with the guidelines set forth by the Australian Code for the Responsible Conduct of Research. Data will be stored online in a de-identified form using the REDCap database system, hosted by the University of Melbourne on a secure server with access rights only granted to the study team. Data stored on REDCap will be password-protected and only accessible to the research team and other authorised persons.

Study data may be inspected by the independent clinical trial monitor and authorised representatives, including the University of Melbourne HREC, Deakin University, the Therapeutic Goods Administration, and the United States Department of Defense, for the purpose of verifying the procedures and the data, and compliance of the trial with GCP guidelines (see also section 13.1). This may include access to identifiable information or protected health information; this access is outlined in the study PICF.

In accordance with Australian privacy law, participants will have the right to request access to their data. After data cleaning procedures are finished, the database on REDCap will be locked. REDCap has an inbuilt feature to adjust and restrict user access as needed. All data will be exported into the appropriate software to enable statistical analysis. The final dataset will be retained by PI Berk at the coordinating site. Contracted researchers (i.e trial coordinator, research assistants) will not have access to the dataset once the REDCap database has been locked, except for as needed at the discretion of PI Berk.

### 11.2.2 Other electronic data

Associated information, such as that which links individuals with their unique code identifier, will be stored separately on the Phoenix Australia server. The study specific storage drive has restricted access, only those who have been identified as they study team may access it. Only the study team will be able to link the study ID and identifying information.

Medical records, when in a digital format, will be stored in secure, password-protected folders on the Phoenix Australia server. Audio recordings of the intake assessment, if consented to, will similarly be stored on a secure, password-protected Phoenix Australia server, and these will be deleted once they have been fidelity-assessed.

Electronic data collected from the ActiGraph LEAP devices will be stored on the CentrePoint app; data will additionally be downloaded as .csv files onto secure servers in study-specific folders at Phoenix Australia at regular intervals. Access will be limited to the study team, as outlined in section 8.3.1 and section 11.1.1.

### 11.2.3 Paper source documents

All paper source documents associated with raw data will be stored in a folder under the participant’s unique code. The de-identified, paper copies of raw data and separately, any identifying information (such as PICFs and any hard copy medical records), will be stored in locked filing cabinets within a locked room at Phoenix Australia. Only the study team will have access to participant files.

The coordinating PI (PI Berk), trial coordinator or their delegate will arrange for the retention of all electronic and hard copy documentation relating to the trial for 15 years. After this time, electronic data files will be deleted and hard copy records will be shredded.

## 11.3 Data sharing plan

The National Institute of Mental Health Data Archive (NDA) will be used as an online data-sharing repository. Participant data will be linked in the shared data repository using an assigned NDA Global Unique Identifier (GUID). The GUID itself is not personally identifiable information or protected health information. All data required for generation of GUIDs will be collected (Appendix 16.23); the GUID is computed locally without any identifying information leaving the study site. The study PICF will include informed consent to NDA data-sharing.

# Data analysis

## 12.1 Analysis of quantitative data

All analyses will be performed by an independent biostatistician under PI Mohebbi’s supervision. This statistician will be blind to group allocation. The analysis will be conducted on an intention-to-treat basis. Analyses will assess the impact of the intervention i.e., between-group (intervention vs placebo) differential change from baseline to week 12 and post-baseline follow-ups in the primary and secondary outcome measures, over the study period. Analyses will use a likelihood-based mixed-effects model, repeated measures approach (MMRM) that accounts for within-participants autocorrelation due to repeated measures nature of the design and multiple centres as a random effect in a multilevel model. Patterns of missing data in the primary and secondary outcomes will be investigated and if there is evidence of non-random missingness, appropriate sensitivity analyses will be performed. Multiple imputations will be used to impute the missing data using all relevant demographics and other important chartists (i.e. comorbidities, substance use etc.) as auxiliary variables in the imputation process to mitigate the impact of non-random missingness. Between-group comparisons using the same approach as the main outcome intervention effect estimation will be used for the sensitivity analysis. In addition, important confounders such as sex, gender, the presence of a comorbid depressive or anxiety disorder, a history of childhood trauma, and site, will be examined in additional exploratory sub-group analyses.

## 12.2 Power calculations

The target sample size is 250 participants, or 125 per arm. For a two-tailed analysis with alpha set at 0.05, the study will have a power of 80% to detect a differential change (i.e. from baseline to week 12) in CAPS-5 score between the Silexan and placebo groups of 5 ± 20 points. The expected effect size of *d* = 0.25 is similar to the effect size of SSRIs in PTSD (*d* = 0.28) (17), noting that a large randomised controlled trial recently found that Silexan and the SSRI sertraline had similar efficacy in treating Major Depressive Disorder (90). Assuming a dropout rate of 10%, we will recruit 278 participants to achieve 250 completers.

## 12.3 Feasibility

We will recruit 8-9 participants per month over a 33-month recruitment period. We anticipate that the favourable side effect profile of Silexan and the adjunctive nature of the trial intervention will assist with recruitment. Our recruitment target appears achievable given the large number of patients with PTSD managed across the four study sites (see section 7.1).

# Study oversight

## 13.1 Clinical monitoring plan

The trial will be conducted in accordance with the International Council for Harmonisation of Technical Requirements for Pharmaceuticals for Human Use (ICH) E6(R2) Good Clinical Practice (GCP) guidelines. The trial coordinator will be responsible for ensuring that trial personnel at all sites have completed up-to-date accredited GCP training.

An independent researcher will be employed as a clinical trial monitor by the coordinating study site, the Institute for Mental and Physical Health and Clinical Translation (IMPACT) at Deakin University, or by Phoenix Australia as appropriate and delegated to by IMPACT. The monitor will verify the compliance of the trial with GCP guidelines at all sites. They will be able to access all study data electronically through the REDCap system.

The monitor will conduct the following monitoring visits:

1. Interim monitoring visits will be conducted throughout the study. In-person visits will occur at each site conducting assessments annually. Remote visits will occur between in-person visits. In remote visits, the trial monitor will contact trial personnel by telephone. At all visits, the trial monitor will verify that:

- participants’ safety, rights and well-being are being protected, including the appropriate use of HREC-approved and up-to-date Participant Information Statement and Consent Forms (PICFs);
- participants included in the trial are eligible and appropriate for the trial; and
- primary outcome study data in the REDCap database are accurate, complete and consistent with any source documentation.

In addition to the above, the Clinical Trial Manager will verify that:

- the research team are conducting the study in accordance with applicable requirements, including the trial protocol, Statement of Work, GCP guidelines and applicable regulatory requirements
- Data entry, completeness and accuracy of secondary outcome data, other outcome data, and investigational product management.

Monitoring roles for the trial monitor, and clinical trial manager, are outlined in a Study Monitoring Plan and checklist.

1. For-cause visits will be conducted as needed to address any unanticipated issues that arise requiring training or remediation or other situations in which a site requires assistance. For-cause visits may be requested by the coordinating Principal Investigator (PI), a study site or the approving HREC. These visits may be on-site or remote as appropriate to the specific issues that trigger the visit.
2. An in-person close-out visit will be conducted at each site after completion of data collection to ensure that all study data and other study documentation are complete and accurate and that all study records have been reconciled.

Monitoring visits at each study site will be conducted in accordance with the below schedule:

| Visit number | Type of visit | Time (months after first participant’s first dose of trial medication) |
| --- | --- | --- |
| 1 | On-site visit | 2 |
| 2 | Remote visit | 8 |
| 3 | On-site visit | 14 |
| 4 | Remote visit | 20 |
| 5 | On-site visit | 26 |
| 6 | Remote visit | 32 |
| 7 | Close-out visit | 38 |

Prior to a monitoring visit, the monitor will send the PI and site personnel:

- a visit confirmation email;
- an agenda;
- a list of site personnel to be available to meet with the monitor during the visit; and
- a list of documentation that will be reviewed.

The PI and site personnel will be expected to secure a workspace for the monitor and to be available during the visits to facilitate monitoring activities. Depending on the amount of study activities that have occurred since the last monitoring visit (including the number of participants who have been enrolled and the number of study sessions that have occurred), visits will take 1-2 days. At the conclusion of the monitoring visit, the monitor will meet with the PI and site personnel for a close-out discussion.

During monitoring visits, the monitor will verify that:

- all participants enrolled in the trial and monitored at that visit have a properly signed and dated PICF;
- all participants enrolled in the trial and monitored at that visit meet the eligibility criteria;
- site personnel have followed the trial protocol, particularly with regard to the primary outcome;
- documentation on REDCap is complete, accurate, consistent and adheres to the trial protocol, including in relation to the primary outcome, and reporting of adverse events and participant withdrawals;
- safety reports comply with the safety monitoring requirements outlined in the trial protocol and NHMRC guidance regarding safety monitoring in clinical trials;
- documentation and reporting of breaches adheres to the principles of ALCOA (attributable, legible, contemporaneous, original, accurate) and is complete and traceable; and
- if any corrective and preventative action plans have been developed, these are being actioned by relevant site personnel.

During monitoring visits, the clinical trial manager will verify that:

- all trial personnel are up-to-date with required training for the trial;
- site personnel have followed the trial protocol, particularly with regard to primary and secondary outcome measures and other measures.
- Investigational product management is up-to-date, and investigational product is stored, managed, provided and accounted for appropriately

The monitor will review the electronic case report forms (CRFs) for the primary outcome on REDCap to confirm their completeness and accuracy and that any corrections have been completed according to GCP requirements and have been signed appropriately. For the first three participants enrolled at each site and thereafter at least one-third of all participants, full (100%) source data verification (SDV) and review of CRF completeness and adherence to study protocol will be performed. For the remaining trial participants, the monitor will decide the level of SDV and process review required.

The monitor will alert the study staff to:

- any discrepancies in the trial documentations

The monitor will document monitoring visit findings and resulting action items in monitoring visit reports, which will be provided to the PI and site personnel within 10 business days of the visit.  The monitor will work with site personnel to resolve any outstanding action items. At a mutually agreed time no later than 4-6 weeks post-visit, the monitor and site personnel will discuss via telephone or email all resolved, in process and pending action items. At this time, the need for, and frequency of, subsequent meetings will be determined.

## 13.2 Data Safety Monitoring Board

The DSMB will be composed of a study-independent psychiatrist, pharmacist and statistician. The DSMB will review the accumulated data from the trial quarterly, including recruitment rates and efficacy and safety data. Details regarding the process for these reviews will be documented in a DSMB charter.

## 13.3 Community-based participatory research

The research team includes an Australian Army veteran with lived experience of PTSD, PI Jensen. PI Jensen served in the Australian Army Transportation Corps from 2007 to 2019 and was deployed to Iraq from 2014 to 2015. He developed PTSD as a result of his experiences in Iraq. He has trialled a range of PTSD treatments, including trauma-focussed psychotherapy, medications and neurostimulation treatments. He has a valuable perspective regarding the limitations of existing PTSD treatments and the need for new treatments. He has provided input into the design of the trial.

PI Jensen will chair a committee called the **Community Advisory Board (CAB).** The CAB will also include a second community member with lived experience of PTSD and two clinician representatives (PI Khanna and PI Roebuck). It will meet with the research team via teleconference on a quarterly basis to provide consultation to the trial and give input regarding the promotion of the trial. It will also assist with translation of the study’s findings.

# Results

We will publish the results of the trial in peer-reviewed scientific journals and present them at academic conferences. Authorship eligibility guidelines for primary outcome, secondary outcome and ancillary publications will be outlined in an authorship document, established in the first year of the trial. We will also take steps to ensure that the trial’s findings are translated into clinical practice.

Once the study has been completed by all participants, a notification letter will be sent to each participant revealing which group of the study (either the Silexan or placebo group) they were in. Additionally, once the data is analysed participants will be sent a letter including the summary of the results of the study. The summary of results will be based on group data from all the participants.

# References

1. Kessler RC, Berglund P, Demler O, Jin R, Merikangas KR, Walters EE. Lifetime prevalence and age-of-onset distributions of DSM-IV disorders in the National Comorbidity Survey Replication. Archives of general psychiatry. 2005;62(6):593-602.

2. Fulton JJ, Calhoun PS, Wagner HR, Schry AR, Hair LP, Feeling N, et al. The prevalence of posttraumatic stress disorder in Operation Enduring Freedom/Operation Iraqi Freedom (OEF/OIF) veterans: A meta-analysis. Journal of anxiety disorders. 2015;31:98-107.

3. Chapman C, Mills K, Slade T, McFarlane AC, Bryant R, Creamer M, et al. Remission from post-traumatic stress disorder in the general population. Psychological medicine. 2012;42(8):1695-703.

4. Jellestad L, Vital NA, Malamud J, Taeymans J, Mueller-Pfeiffer C. Functional impairment in posttraumatic stress disorder: A systematic review and meta-analysis. Journal of psychiatric research. 2021;136:14-22.

5. Ivanova JI, Birnbaum HG, Chen L, Duhig AM, Dayoub EJ, Kantor ES, et al. Cost of post-traumatic stress disorder vs major depressive disorder among patients covered by medicaid or private insurance. The American journal of managed care. 2011;17(8):e314-23.

6. Health VAOoM, Suicide Prevention. 2021 National Veteran Suicide Prevention Annual Report. 2021.

7. Phoenix Australia. Australian Guidelines for the Treatment of Acute Stress Disorder and Posttraumatic Stress Disorder. [Online]; 2020.

8. International Society for Traumatic Stress Studies Guidelines Committee. Posttraumatic stress disorder prevention and treatment guidelines methodology and recommendations. [Online]; 2018.

9. American Psychological Association. Clinical Practice Guideline for the Treatment of Posttraumatic Stress Disorder (PTSD) in Adults Guideline Development Panel for the Treatment of PTSD in Adults 2017.

10. Shea MT, Krupnick JL, Belsher BE, Schnurr PP. Non-trauma-focused psychotherapies for the treatment of PTSD: A descriptive review. Current Treatment Options in Psychiatry. 2020;7:242-57.

11. Finley EP, Garcia HA, Ketchum NS, McGeary DD, McGeary CA, Stirman SW, et al. Utilization of evidence-based psychotherapies in Veterans Affairs posttraumatic stress disorder outpatient clinics. Psychological services. 2015;12(1):73.

12. Rosen CS, Eftekhari A, Crowley JJ, Smith BN, Kuhn E, Trent L, et al. Maintenance and reach of exposure psychotherapy for posttraumatic stress disorder 18 months after training. Journal of traumatic stress. 2017;30(1):63-70.

13. Varker T, Jones KA, Arjmand H-A, Hinton M, Hiles SA, Freijah I, et al. Dropout from guideline-recommended psychological treatments for posttraumatic stress disorder: A systematic review and meta-analysis. Journal of Affective Disorders Reports. 2021;4:100093.

14. Meis LA, Noorbaloochi S, Hagel Campbell EM, Erbes CR, Polusny MA, Velasquez TL, et al. Sticking it out in trauma-focused treatment for PTSD: It takes a village. Journal of Consulting and Clinical Psychology. 2019;87(3):246.

15. Schottenbauer MA, Glass CR, Arnkoff DB, Tendick V, Gray SH. Nonresponse and dropout rates in outcome studies on PTSD: Review and methodological considerations. Psychiatry: Interpersonal and biological processes. 2008;71(2):134-68.

16. Steenkamp MM, Litz BT, Hoge CW, Marmar CR. Psychotherapy for military-related PTSD: A review of randomized clinical trials. Jama. 2015;314(5):489-500.

17. Hoskins MD, Bridges J, Sinnerton R, Nakamura A, Underwood JF, Slater A, et al. Pharmacological therapy for post-traumatic stress disorder: a systematic review and meta-analysis of monotherapy, augmentation and head-to-head approaches. European Journal of Psychotraumatology. 2021;12(1):1802920.

18. Huang Z-D, Zhao Y-F, Li S, Gu H-Y, Lin L-L, Yang Z-Y, et al. Comparative efficacy and acceptability of pharmaceutical management for adults with post-traumatic stress disorder: a systematic review and meta-analysis. Frontiers in pharmacology. 2020;11:559.

19. Edinoff AN, Akuly HA, Hanna TA, Ochoa CO, Patti SJ, Ghaffar YA, et al. Selective serotonin reuptake inhibitors and adverse effects: a narrative review. Neurology International. 2021;13(3):387-401.

20. Serretti A, Chiesa A. Treatment-emergent sexual dysfunction related to antidepressants: a meta-analysis. Journal of clinical psychopharmacology. 2009;29(3):259-66.

21. Haddad PM, Sharma SG. Adverse effects of atypical antipsychotics: differential risk and clinical implications. CNS drugs. 2007;21:911-36.

22. Müller WE, Sillani G, Schuwald A, Friedland K. Pharmacological basis of the anxiolytic and antidepressant properties of Silexan®, an essential oil from the flowers of lavender. Neurochemistry International. 2021;143:104899.

23. Da Porto C, Decorti D, Kikic I. Flavour compounds of Lavandula angustifolia L. to use in food manufacturing: Comparison of three different extraction methods. Food Chemistry. 2009;112(4):1072-8.

24. Schuwald AM, Nöldner M, Wilmes T, Klugbauer N, Leuner K, Müller WE. Lavender oil-potent anxiolytic properties via modulating voltage dependent calcium channels. PloS one. 2013;8(4):e59998.

25. Blazon M, LaCarubba B, Bunda A, Czepiel N, Mallat S, Londrigan L, et al. N-type calcium channels control GABAergic transmission in brain areas related to fear and anxiety. OBM neurobiology. 2021;5(1).

26. Marks WN, Zabder NK, Snutch TP, Howland JG. T-type calcium channels regulate the acquisition and recall of conditioned fear in male, Wistar rats. Behavioural Brain Research. 2020;393:112747.

27. Baldinger P, Höflich AS, Mitterhauser M, Hahn A, Rami-Mark C, Spies M, et al. Effects of Silexan on the serotonin-1A receptor and microstructure of the human brain: a randomized, placebo-controlled, double-blind, cross-over study with molecular and structural neuroimaging. International Journal of Neuropsychopharmacology. 2015;18(4):pyu063.

28. Ballesteros J, Callado LF. Effectiveness of pindolol plus serotonin uptake inhibitors in depression: a meta-analysis of early and late outcomes from randomised controlled trials. Journal of Affective Disorders. 2004;79(1-3):137-47.

29. Friedland K, Silani G, Schuwald A, Stockburger C, Koch E, Nöldner M, et al. Neurotrophic properties of silexan, an essential oil from the flowers of lavender-preclinical evidence for antidepressant-like properties. Pharmacopsychiatry. 2021;54(01):37-46.

30. Nair A, Vaidya V. Cyclic AMP response element binding protein and brain-derived neurotrophic factor: molecules that modulate our mood? Journal of biosciences. 2006;31:423-34.

31. Müller W, Schuwald A, Nöldner M, Kasper S, Friedland K. Pharmakologische Grundlagen der therapeutischen Anwendung von Silexan (Lasea®). Psychopharmakotherapie. 2015;22(1):3-14.

32. Dimpfel W, Wedekind W, Dienel A. Cerebral bioavailability of silexan—a quantitative EEG study in healthy volunteers. Open Journal of Psychiatry. 2015;5(03):285.

33. Bickers D, Calow P, Greim H, Hanifin J, Rogers A, Saurat J, et al. A toxicologic and dermatologic assessment of linalool and related esters when used as fragrance ingredients. Food and chemical toxicology. 2003;41(7):919-42.

34. Kasper S, Gastpar M, Müller WE, Volz H-P, Möller H-J, Dienel A, et al. Efficacy and safety of silexan, a new, orally administered lavender oil preparation, in subthreshold anxiety disorder-evidence from clinical trials. Wiener medizinische Wochenschrift (1946). 2010;160(21-22):547-56.

35. Kumar V. Characterization of anxiolytic and neuropharmacological activities of Silexan. Wiener Medizinische Wochenschrift (1946). 2013;163(3-4):89-94.

36. Chioca LR, Antunes VD, Ferro MM, Losso EM, Andreatini R. Anosmia does not impair the anxiolytic-like effect of lavender essential oil inhalation in mice. Life sciences. 2013;92(20-21):971-5.

37. Yap WS, Dolzhenko AV, Jalal Z, Hadi MA, Khan TM. Efficacy and safety of lavender essential oil (Silexan) capsules among patients suffering from anxiety disorders: A network meta-analysis. Scientific reports. 2019;9(1):18042.

38. Woelk H, Schläfke S. A multi-center, double-blind, randomised study of the Lavender oil preparation Silexan in comparison to Lorazepam for generalized anxiety disorder. Phytomedicine. 2010;17(2):94-9.

39. Kasper S, Gastpar M, Müller WE, Volz H-P, Möller H-J, Schläfke S, et al. Lavender oil preparation Silexan is effective in generalized anxiety disorder–a randomized, double-blind comparison to placebo and paroxetine. International Journal of Neuropsychopharmacology. 2014;17(6):859-69.

40. Möller H-J, Volz H-P, Dienel A, Schläfke S, Kasper S. Efficacy of Silexan in subthreshold anxiety: meta-analysis of randomised, placebo-controlled trials. European archives of psychiatry and clinical neuroscience. 2019;269:183-93.

41. Bandelow B, Allgulander C, Baldwin DS, Costa DLdC, Denys D, Dilbaz N, et al. World Federation of Societies of Biological Psychiatry (WFSBP) guidelines for treatment of anxiety, obsessive-compulsive and posttraumatic stress disorders–Version 3. Part I: Anxiety disorders. The World Journal of Biological Psychiatry. 2023;24(2):79-117.

42. Bartova L, Dold M, Volz H-P, Seifritz E, Möller H-J, Kasper S. Beneficial effects of Silexan on co-occurring depressive symptoms in patients with subthreshold anxiety and anxiety disorders: randomized, placebo-controlled trials revisited. European Archives of Psychiatry and Clinical Neuroscience. 2023;273(1):51-63.

43. von Känel R, Kasper S, Bondolfi G, Holsboer‐Trachsler E, Hättenschwiler J, Hatzinger M, et al. Therapeutic effects of Silexan on somatic symptoms and physical health in patients with anxiety disorders: A meta‐analysis. Brain and behavior. 2021;11(4):e01997.

44. Code of Federal Regulations Title 21, Volume 3, Part 182, 21CFR182.20. <https://www.accessdata.fda.gov/scripts/cdrh/cfdocs/cfcfr/cfrsearch.cfm?fr=182.20>.

45. Doroshyenko O, Rokitta D, Zadoyan G, Klement S, Schläfke S, Dienel A, et al. Drug cocktail interaction study on the effect of the orally administered lavender oil preparation silexan on cytochrome P450 enzymes in healthy volunteers. Drug Metabolism and Disposition. 2013;41(5):987-93.

46. Heger-Mahn D, Pabst G, Dienel A, Schläfke S, Klipping C. No interacting influence of lavender oil preparation silexan on oral contraception using an ethinyl estradiol/levonorgestrel combination. Drugs in R&D. 2014;14:265-72.

47. Seifritz E, Möller H-J, Volz H-P, Müller WE, Hopyan T, Wacker A, et al. No abuse potential of Silexan in healthy recreational drug users: a randomized controlled trial. International journal of neuropsychopharmacology. 2021;24(3):171-80.

48. Gastpar M, Müller W, Volz H, Möller H, Schläfke S, Dienel A, et al. Silexan does not cause withdrawal symptoms even when abruptly discontinued. International journal of psychiatry in clinical practice. 2017;21(3):177-80.

49. Möller H-J, Volz H-P, Seifritz E, Müller H, Kenntner-Mabiala R, Kaussner Y, et al. Silexan does not affect driving performance after single and multiple dose applications: Results from a double-blind, placebo and reference-controlled study in healthy volunteers. Journal of psychiatric research. 2021;136:543-51.

50. Zoellner LA, Ojalehto HJ, Rosencrans P, Walker RW, Garcia NM, Sheikh IS, et al. Anxiety and fear in PTSD. In: Tull M, Kimbrel N, editors. Emotion in posttraumatic stress disorder: Etiology, assessment, neurobiology, and treatment

Academic Press; 2020.

51. Uehleke B, Schaper S, Dienel A, Schlaefke S, Stange R. Phase II trial on the effects of Silexan in patients with neurasthenia, post-traumatic stress disorder or somatization disorder. Phytomedicine. 2012;19(8-9):665-71.

52. Levi O, Ben Yehuda A, Pine DS, Bar-Haim Y. A sobering look at treatment effectiveness of military-related posttraumatic stress disorder. Clinical Psychological Science. 2022;10(4):690-9.

53. Weathers FW, Bovin MJ, Lee DJ, Sloan DM, Schnurr PP, Kaloupek DG, et al. The Clinician-Administered PTSD Scale for DSM–5 (CAPS-5): Development and initial psychometric evaluation in military veterans. Psychological assessment. 2018;30(3):383.

54. Weathers F, Blake D, Schnurr P, Kaloupek D, Marx B, Keane T. The life events checklist for DSM-5 (LEC-5). 2013.

55. Gray MJ, Litz BT, Hsu JL, Lombardo TW. Psychometric properties of the life events checklist. Assessment. 2004;11(4):330-41.

56. Weathers FW, Litz BT, Keane TM, Palmieri PA, Marx BP, Schnurr PP. The ptsd checklist for dsm-5 (pcl-5). Scale available from the National Center for PTSD at www ptsd va gov. 2013;10(4):206.

57. Blevins CA, Weathers FW, Davis MT, Witte TK, Domino JL. The posttraumatic stress disorder checklist for DSM‐5 (PCL‐5): Development and initial psychometric evaluation. Journal of traumatic stress. 2015;28(6):489-98.

58. Zanarini MC, Vujanovic AA, Parachini EA, Boulanger JL, Frankenburg FR, Hennen J. McLean Screening Instrument for Borderline Personality Disorder. Journal of Personality Disorders. 2003.

59. Sheehan DV, Lecrubier Y, Sheehan KH, Amorim P, Janavs J, Weiller E, et al. The Mini-International Neuropsychiatric Interview (MINI): the development and validation of a structured diagnostic psychiatric interview for DSM-IV and ICD-10. Journal of clinical psychiatry. 1998;59(20):22-33.

60. Sheehan D. The mini-international neuropsychiatric interview, version 7.0 for DSM-5 (MINI 7.0). Jacksonville, FL: Medical Outcomes Systems. 2014.

61. Hunt JC, Chesney SA, Jorgensen TD, Schumann NR, deRoon-Cassini TA. Exploring the gold-standard: Evidence for a two-factor model of the Clinician Administered PTSD Scale for the DSM–5. Psychological trauma: Theory, research, practice, and policy. 2018;10(5):551.

62. Hamilton M. Diagnosis and rating of anxiety. Br J Psychiatry. 1969;3(special issue):76-9.

63. Hamilton M. The assessment of anxiety states by rating. British journal of medical psychology. 1959.

64. Busner J, Targum SD. The clinical global impressions scale: applying a research tool in clinical practice. Psychiatry (edgmont). 2007;4(7):28.

65. Guy W. Clinical global impressions scale. Psychiatry. 1976.

66. Beck AT, Steer RA, Carbin MG. Psychometric properties of the Beck Depression Inventory: Twenty-five years of evaluation. Clinical psychology review. 1988;8(1):77-100.

67. Mahoney MR, Farmer C, Sinclair S, Sung S, Dehaut K, Chung JY. Utilization of the DSM-5 self-rated level 1 cross-cutting symptom measure-adult to screen healthy volunteers for research studies. Psychiatry research. 2020;286:112822.

68. Üstün TB, Chatterji S, Kostanjsek N, Rehm J, Kennedy C, Epping-Jordan J, et al. Developing the World Health Organization disability assessment schedule 2.0. Bulletin of the World Health Organization. 2010;88:815-23.

69. Mokdad AH. The behavioral risk factors surveillance system: past, present, and future. Annual review of public health. 2009;30:43-54.

70. Ford DC, Merrick MT, Parks SE, Breiding MJ, Gilbert LK, Edwards VJ, et al. Examination of the factorial structure of adverse childhood experiences and recommendations for three subscale scores. Psychology of violence. 2014;4(4):432.

71. Buysse DJ, Reynolds III CF, Monk TH, Berman SR, Kupfer DJ. The Pittsburgh Sleep Quality Index: a new instrument for psychiatric practice and research. Psychiatry research. 1989;28(2):193-213.

72. Germain A, Hall M, Krakow B, Shear MK, Buysse DJ. A brief sleep scale for posttraumatic stress disorder: Pittsburgh Sleep Quality Index Addendum for PTSD. Journal of anxiety disorders. 2005;19(2):233-44.

73. Han C, Pae C-U, Patkar AA, Masand PS, Kim KW, Joe S-H, et al. Psychometric properties of the Patient Health Questionnaire–15 (PHQ–15) for measuring the somatic symptoms of psychiatric outpatients. Psychosomatics. 2009;50(6):580-5.

74. Vogt D, Smith BN, King LA, King DW, Knight J, Vasterling JJ. Deployment risk and resilience inventory‐2 (DRRI‐2): An updated tool for assessing psychosocial risk and resilience factors among service members and veterans. Journal of traumatic stress. 2013;26(6):710-7.

75. Richardson JR, Peacock SJ, Hawthorne G, Iezzi A, Elsworth G, Day NA. Construction of the descriptive system for the assessment of quality of life AQoL-6D utility instrument. Health and quality of life outcomes. 2012;10(1):1-9.

76. Richardson JD, N, Hawthorne G, Peacock S, Iezzi A. AQoL-6D <http://www.aqol.com.au/aqolquestionnaires/56.html> [

77. Sherbourne CD, Stewart AL. The MOS social support survey. Social science & medicine. 1991;32(6):705-14.

78. Kroenke K, Spitzer RL, Williams JB. The PHQ‐9: validity of a brief depression severity measure. Journal of general internal medicine. 2001;16(9):606-13.

79. McDowell I. Measuring health: a guide to rating scales and questionnaires: Oxford university press; 2006.

80. Fazio AF. A concurrent validational study of the NCHS General Well-Being Schedule. 1977.

81. Spitzer RL, Kroenke K, Williams JB, Löwe B. A brief measure for assessing generalized anxiety disorder: the GAD-7. Archives of internal medicine. 2006;166(10):1092-7.

82. Saunders JB, Aasland OG, Babor TF, De la Fuente JR, Grant M. Development of the alcohol use disorders identification test (AUDIT): WHO collaborative project on early detection of persons with harmful alcohol consumption‐II. Addiction. 1993;88(6):791-804.

83. Aertgeerts B, Buntinx F, Ansoms S, Fevery J. Screening properties of questionnaires and laboratory tests for the detection of alcohol abuse or dependence in a general practice population. British journal of general practice. 2001;51(464):206-17.

84. Steinbauer JR, Cantor SB, Holzer III CE, Volk RJ. Ethnic and sex bias in primary care screening tests for alcohol use disorders. Annals of Internal Medicine. 1998;129(5):353-62.

85. Slightam C, Petrowski K, Jamison AL, Keller M, Bertram F, Kim S, et al. Assessing sleep quality using self‐report and actigraphy in PTSD. Journal of sleep research. 2018;27(3):e12632.

86. Buckley TC, Holohan D, Greif JL, Bedard M, Suvak M. Twenty-four-hour ambulatory assessment of heart rate and blood pressure in chronic PTSD and non-PTSD veterans. Journal of Traumatic Stress. 2004;17:163-71.

87. Code of Federal Regulations

88. Gangemi S, Minciullo PL, Miroddi M, Chinou I, Calapai G, Schmidt RJ. Contact dermatitis as an adverse reaction to some topically used European herbal medicinal products–Part 2: Echinacea purpurea–Lavandula angustifolia. Contact dermatitis. 2015;72(4):193-205.

89. Gantner G, Spiess D, Randecker E, Quack Lötscher KC, Simões-Wüst AP. Use of herbal medicines for the treatment of mild mental disorders and/or symptoms during pregnancy: A cross-sectional survey. Frontiers in pharmacology. 2021;12:729724.

90. Kasper S, Volz H-P, Möller H-J, Schläfke S, Klement S, Anghelescu I-G, et al. Lavender oil preparation Silexan is effective in mild-to-moderate major depression: a randomized, placebo-and reference-controlled trial. European archives of psychiatry and clinical neuroscience. 2024:1-11.

# Appendices

## 16.1 Therapeutic Goods Administration – ARTG Entry – Seremind

N.B: see <https://www.tga.gov.au/resources/artg/259012>


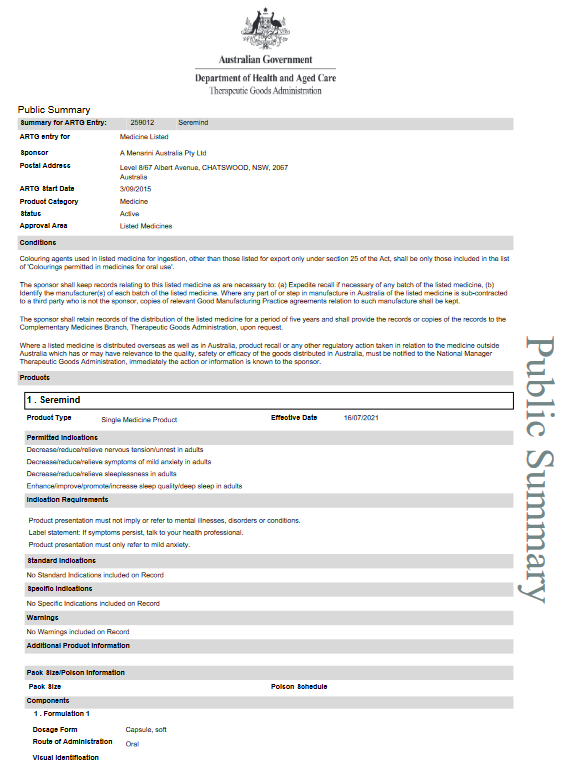


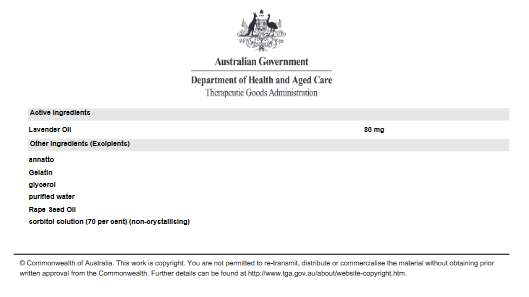


## 16.2 Food and Drug Administration Approval of Lavender


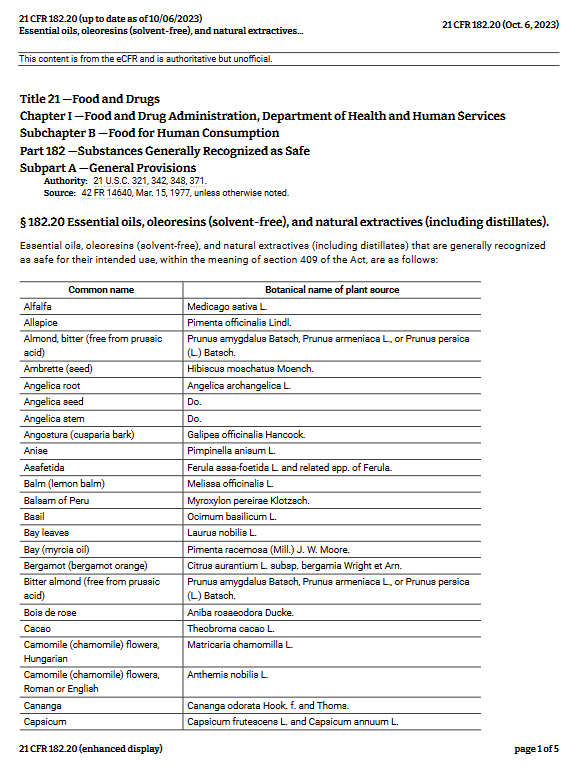

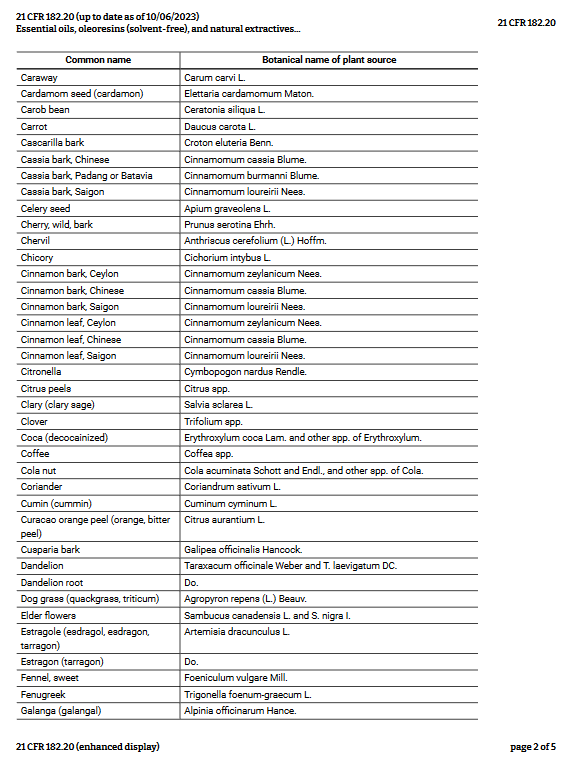


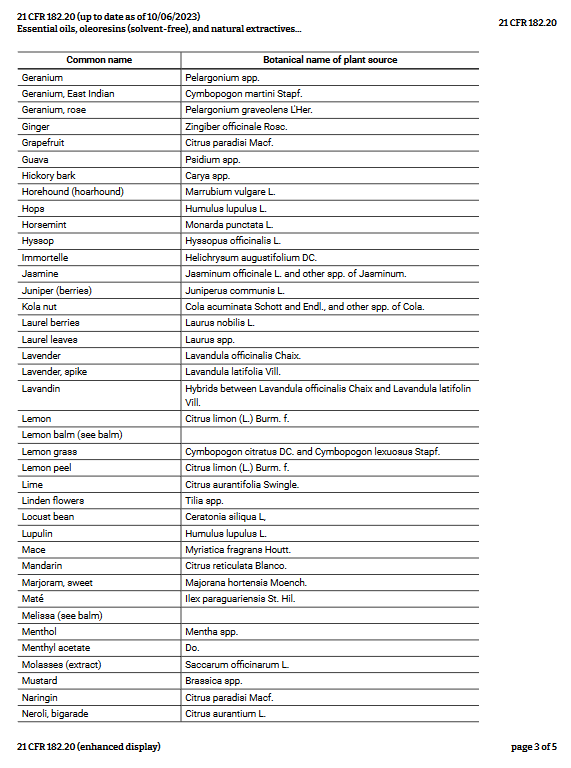


## 16.3 Demographics

NB: Below is an example of the nature and style of questions that will be asked via a REDCap questionnaire in the current study

1. What is your date of birth?
2. What is your biological sex?

Male

Female

Other

Please specify

Prefer not to answer

1. What best describes your gender identity?

Man

Woman

Non-binary

Transgender

None of these describe me, and I want to specify

Please specify

Prefer not to answer

1. What is your height in cm?
2. What is your weight in kg?
3. What is your dominant hand, or the hand you write with?
   1. Left-handed
   2. Right-handed
4. What is your marital/partner status?

Married

Divorced

Widowed

Separated

Never married

A member of an unmarried couple

Prefer not to answer

1. What is your highest completed degree of education?

Primary school graduate

High school graduate

Bachelor’s degree

Occupational, technical or vocational college

Master’s degree

Professional school degree

Doctoral degree

1. Are you:

Aboriginal

Torres Strait Islander

Neither

1. What is your employment status?

Full-time

Part-time

Temporarily on sick or maternity leave (including WorkCover leave)Unemployed

Retired

Receiving disability pension or payments Not in labour force (e.g student, keeping house, etc)

1. What is your best estimate of the total income of all family members from all sources, before taxes, in the last calendar year? (including benefits, pensions, family payments and superannuation for all household members)
   1. Note: please provide your best estimate, please enter ‘999999’ if you would prefer not to answer.
2. Have you ever smoked a cigarette or vape, even one or two puffs?

Yes - cigarette

Do you now smoke cigarettes…

Every day

Some days

Not at all

Prefer not to answer

Yes – vape

Do you now vape…

Every day

Some days

Not at all

Prefer not to answer

No

Don’t know

Prefer not to answer

**Next, we would like to know about any current pharmaceutical and non-pharmaceutical treatments or medications you are currently taking, including over-the-counter medication, vitamins or herbal remedies, acupuncture or other alternative treatments or elective surgery.**

**Medications**

*Both prescription and over-the-counter*

| **Drug Name** | **Reason for taking**  (e.g. anxiety) | **Dose**  (e.g. 50mg) | **Frequency**  (e.g. every day) | **Route**  (e.g. orally) |
| --- | --- | --- | --- | --- |
|  |  |  |  |  |
|  |  |  |  |  |
|  |  |  |  |  |
|  |  |  |  |  |
|  |  |  |  |  |

**Supplements**

| **Supplement** | **Dose**  (e.g. 50mg) | **Frequency**  (e.g. every day) | **Route**  (e.g. orally) |
| --- | --- | --- | --- |
|  |  |  |  |
|  |  |  |  |
|  |  |  |  |
|  |  |  |  |
|  |  |  |  |

**Non-Medication Therapy**

*Therapy examples: physiotherapy, occupational therapy, psychotherapy or counselling, speech therapy, acupuncture.*

| **Therapy** | **Frequency** (e.g. every day) | **Start Date** |
| --- | --- | --- |
|  |  |  |
|  |  |  |
|  |  |  |
|  |  |  |
|  |  |  |

**Recreational Drug Use**

*Note: This, and all other information, will be stored in a deidentified form and will be kept confidential, except in circumstances where its disclosure is legally compelled (e.g. under a court subpoena)*

| **Type** | **Frequency** (e.g. every day) | **Start Date** |
| --- | --- | --- |
|  |  |  |
|  |  |  |
|  |  |  |
|  |  |  |
|  |  |  |

**Surgical History**

Performed or planned

| **Procedure** | **Year** |
| --- | --- |
|  |  |
|  |  |
|  |  |
|  |  |
|  |  |

**Military Service Demographics**

1. Have you ever served in the military?

No [skip items 2 – 9 and continue to next questionnaire]

Yes

2. In which branch of the service did you serve? (Mark all that apply)

Royal Australian Navy

Australian Army

Royal Australian Air Force

Other

Please specify

3. How long did you serve in active duty?

Years [ ]

Months [ ]

4. How long did you serve in Reserve?

Years [ ]

Months [ ]

1. What was your highest rank at separation?

Enlisted

Non-Commissioned Officer

Officer

6. Where were you stationed?

Africa

Asia/South Pacific

Caribbean

Eastern Europe

Mexico

Middle East

Northern/Central Europe

Southern Europe/Mediterranean Basin

South/Central America

USA/Canada

Other

Please specify

8. How many times were you deployed?

9. How many combat or war zone tours have you served?

## 16.4 McLean Screening Instrument for BPD


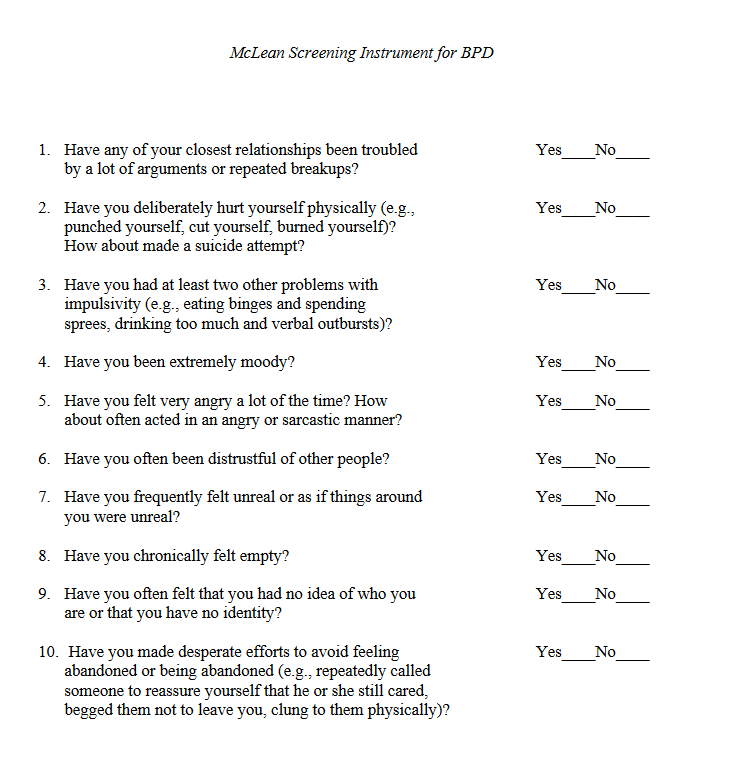


## 16.5 Life Events Checklist for DSM-5 (LEC-5) with Criterion A

N.B. See Appendix 16.18 for PCL-5 component


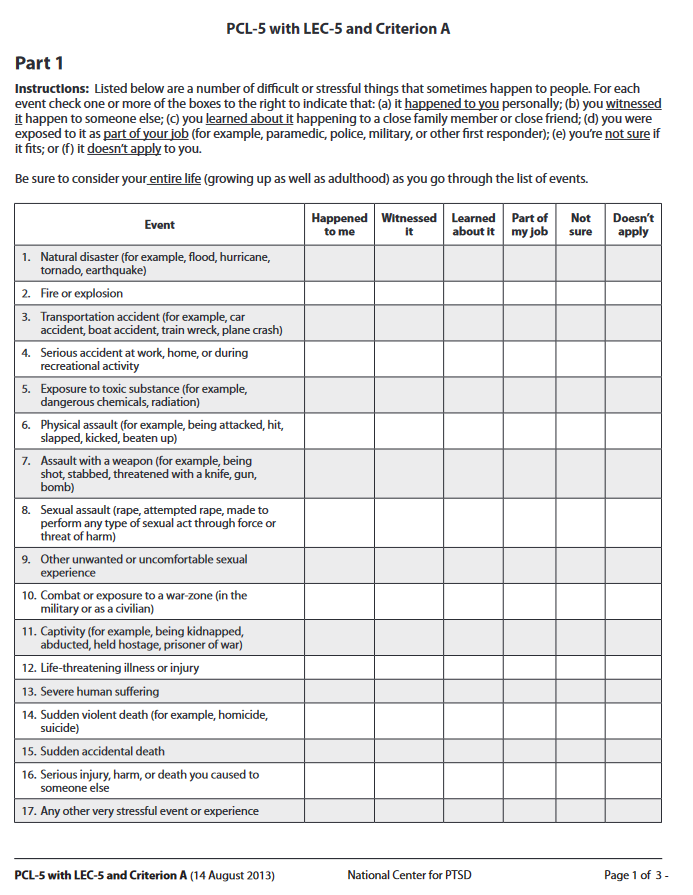


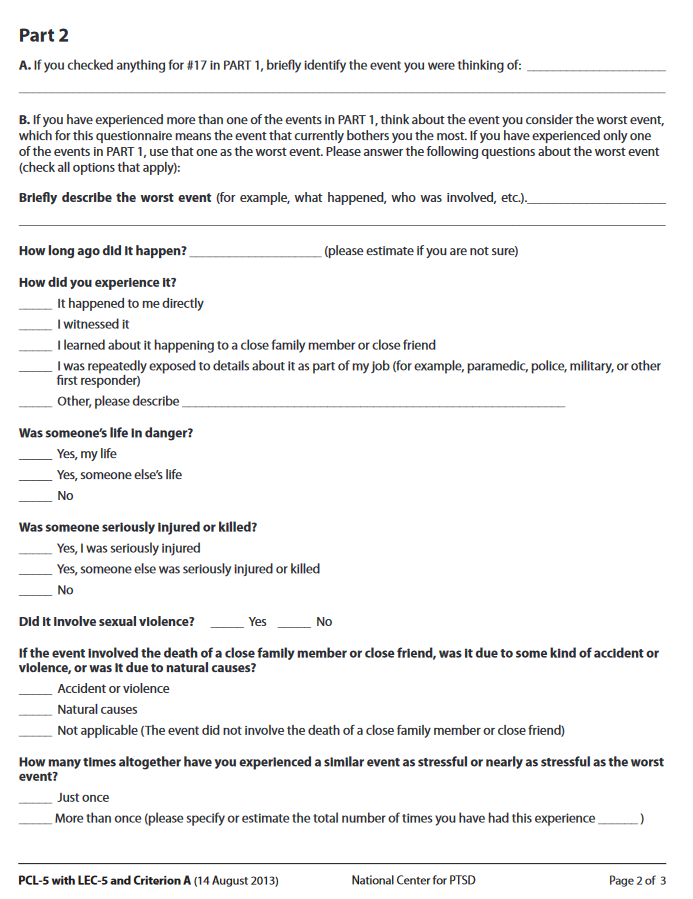


## 16.6 Beck Depression Inventory-II (BDI-II)

N.B: Below is an example of the type of questions that are asked as part of the BDI-II assessment.


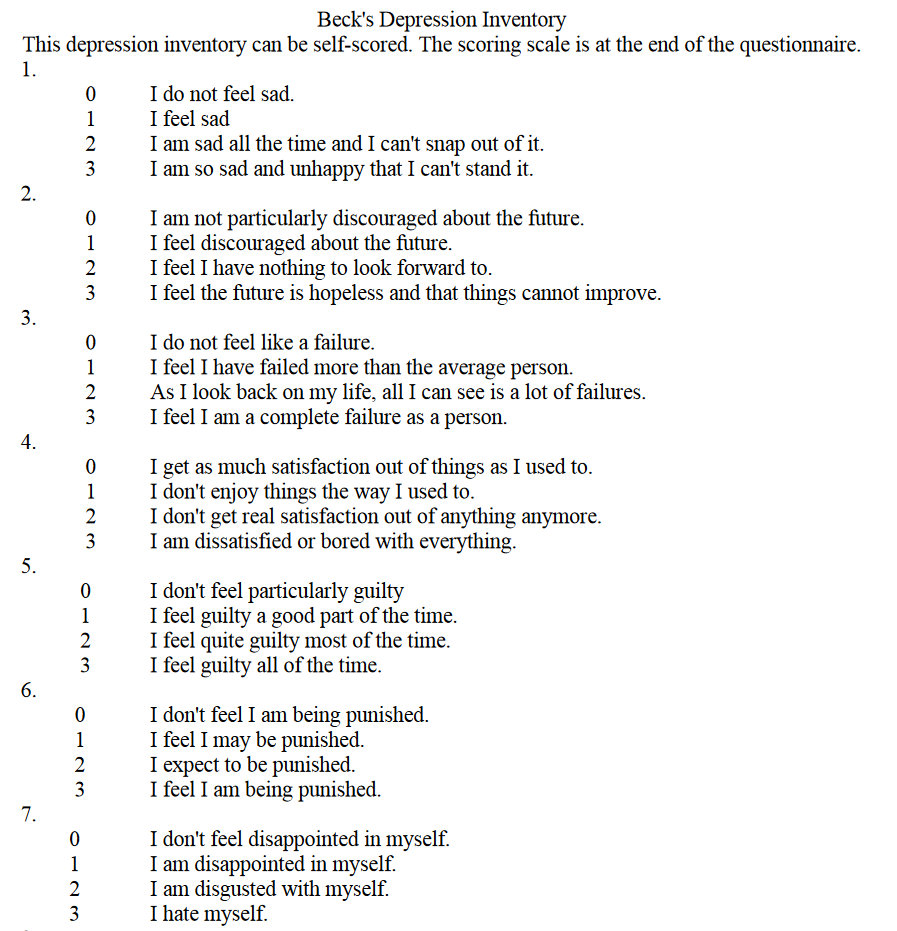


## Hamilton Anxiety Rating Scale (HAM-A)


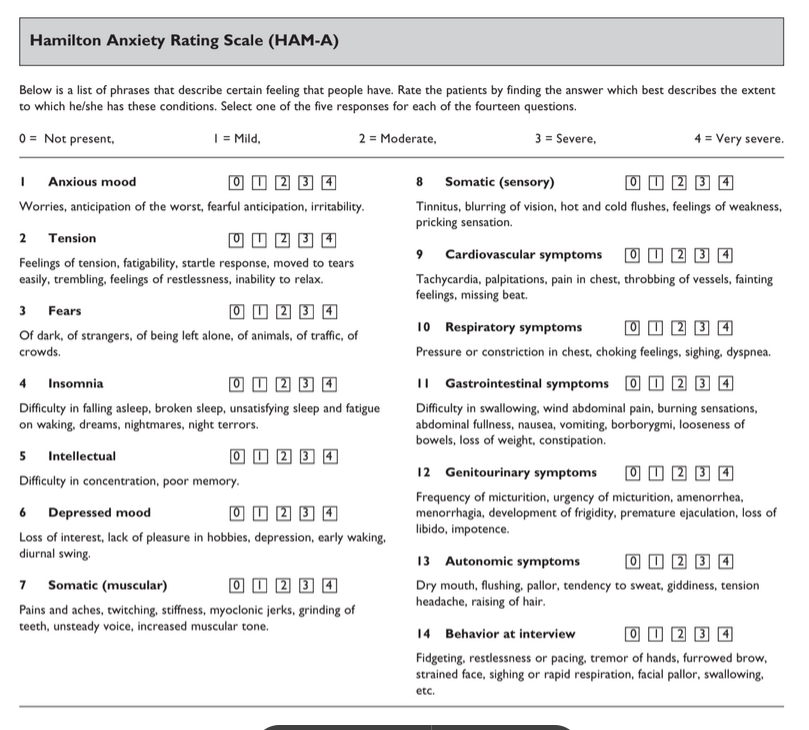


## 16.8 DSM-5-TR Self-Rated Level 1 Cross-Cutting Symptom Measure—Adult (CCSM)


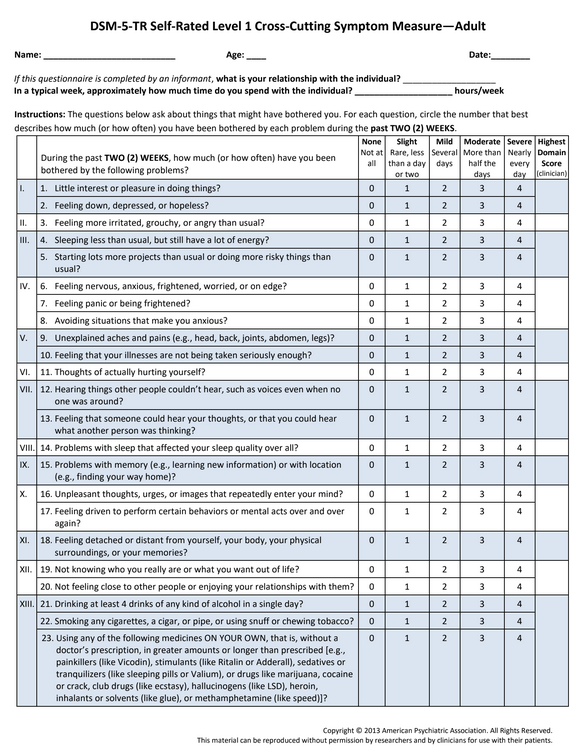


## 16.9 Clinical Global Impression Scale/ Patient Global Impression of Change


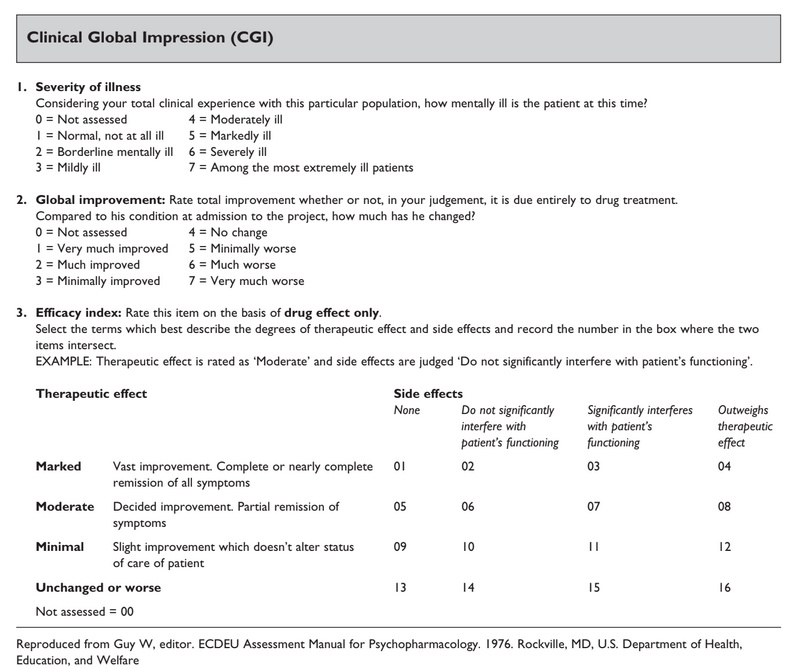


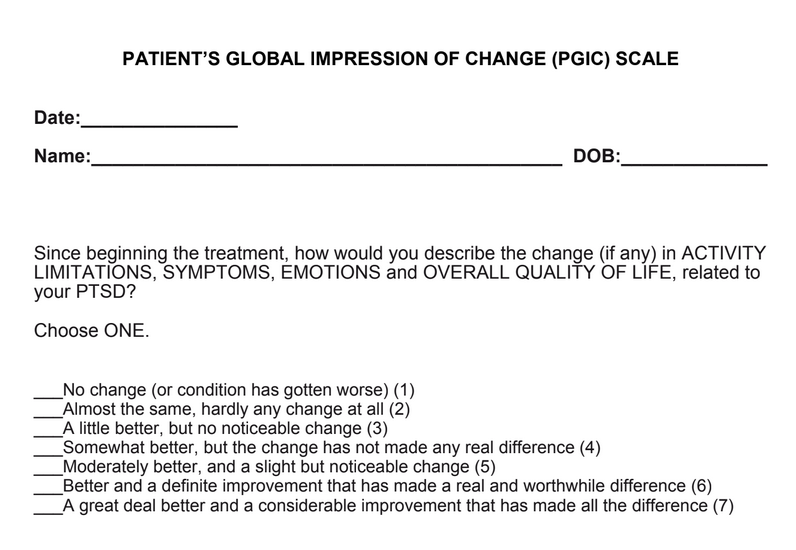


## 16.10 World Health Organization Disability Assessment Schedule 2.0


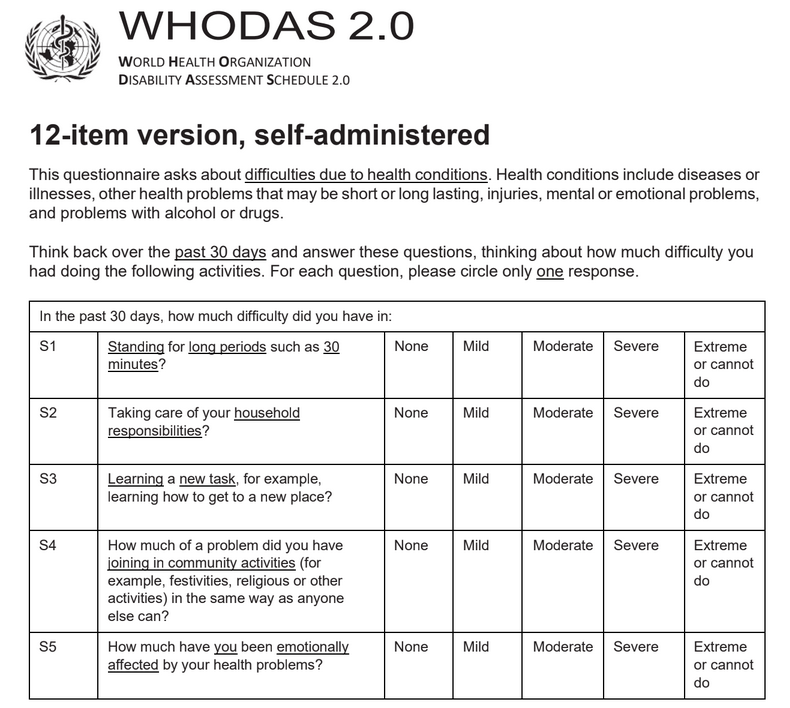


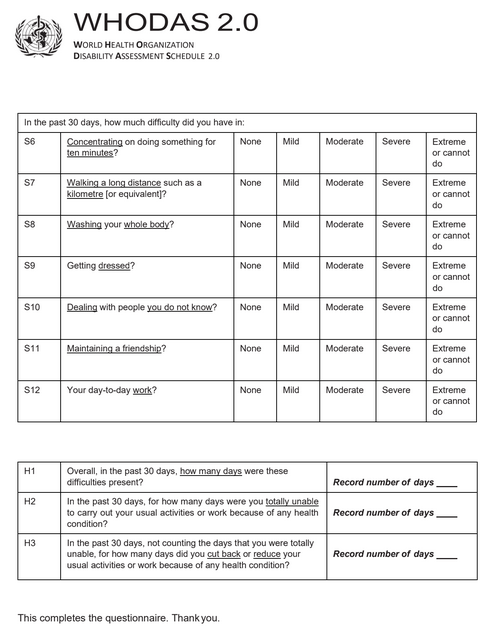


## 16.11 Pittsburgh Sleep Quality Index

N.B: In addition, the 10 additional questions from the PSQI-Addendum for PTSD (Germain et al., *Journal of Anxiety Disorders.* 2005) will also be included.


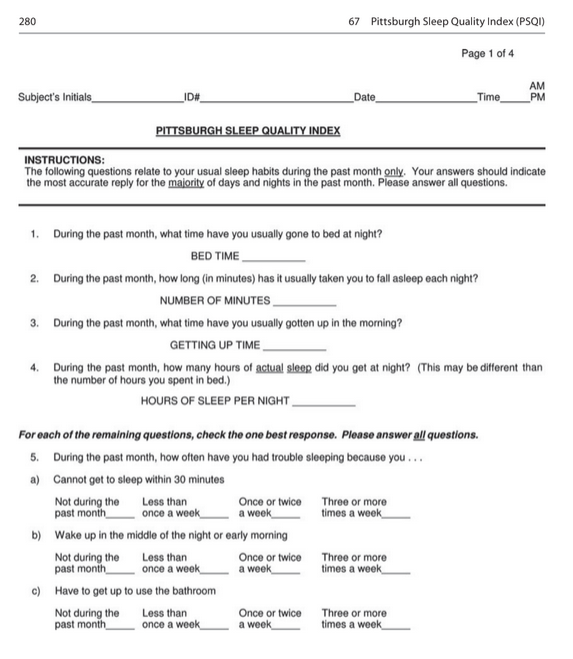


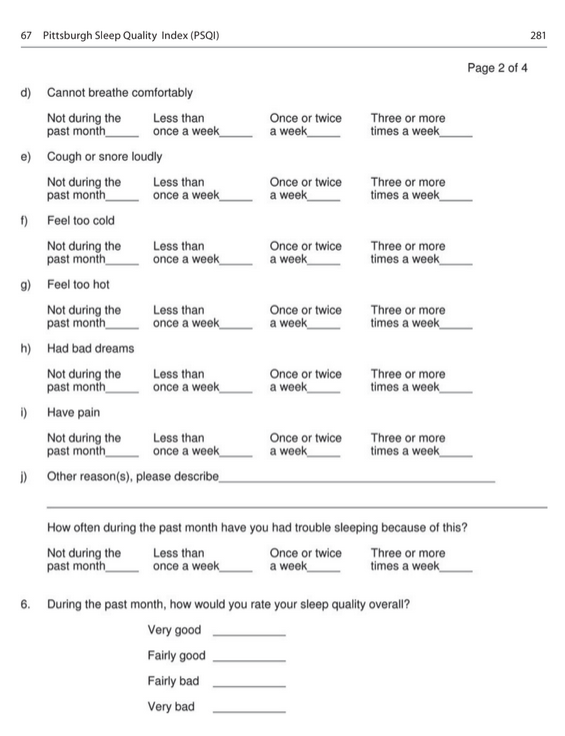


## 16.12 Patient Health Questionnaire-15


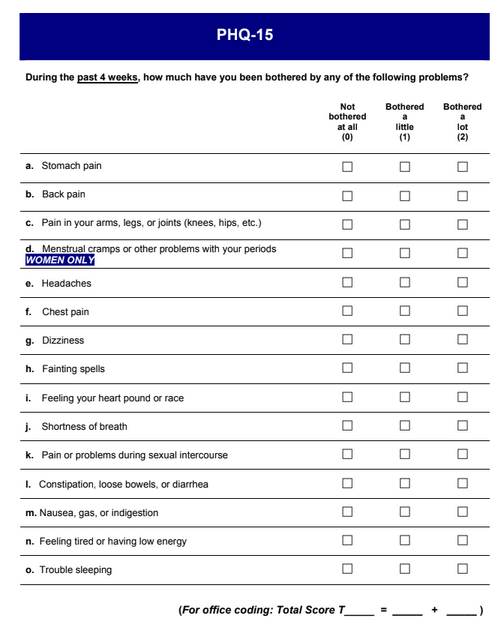


## 16.13 Deployment Risk & Resilience Inventory-2

N.B: Below is an example of the type of questions that are asked as part of the DRRI-2 assessment.


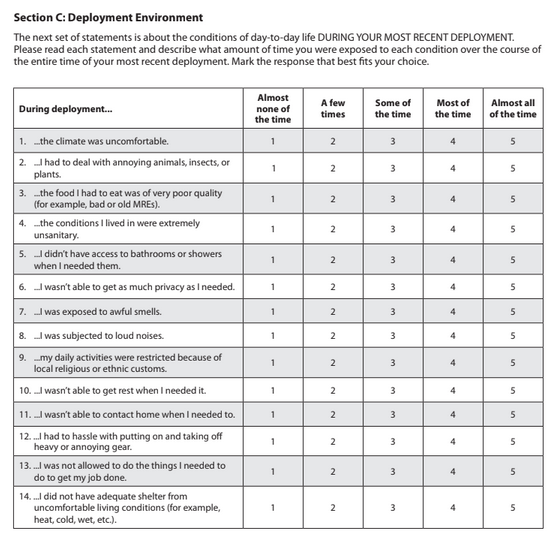


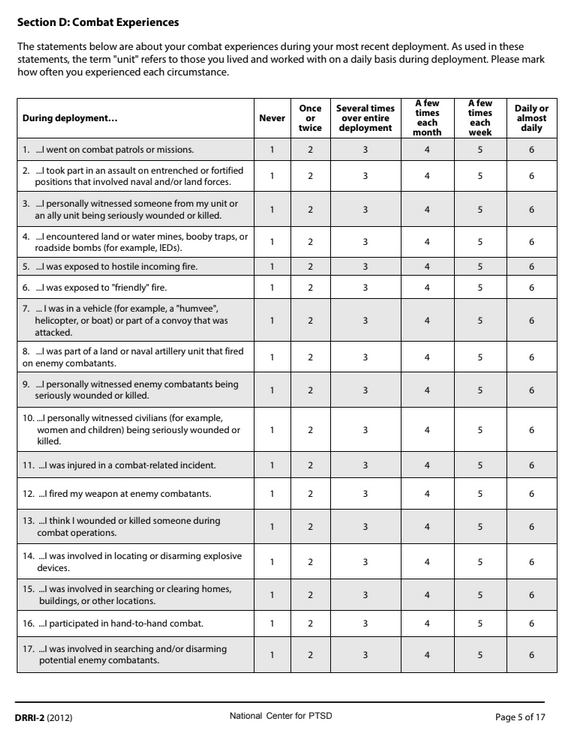


## 16.14 Assessment of Quality of Life-6D scale

N.B: Below is an example of the type of questions that are asked as part of the AQoL-6D assessment.


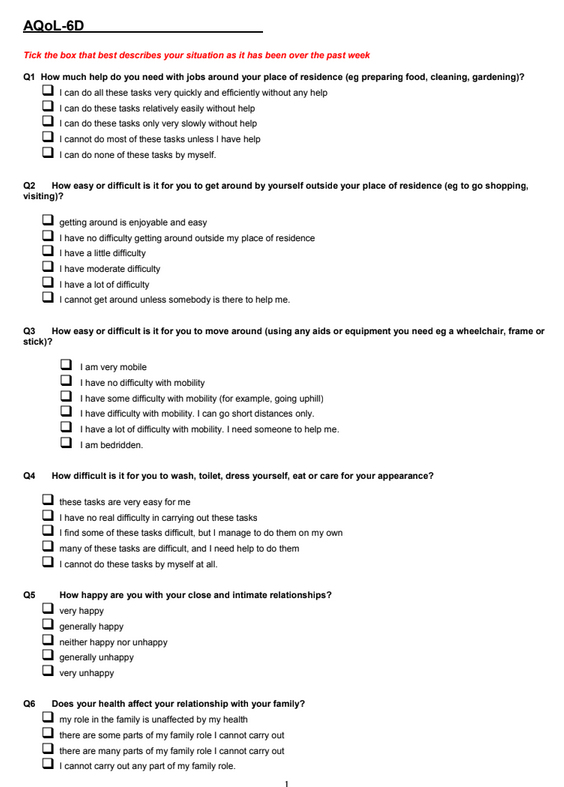


## 16.15 Behavioural Risk Factor Surveillance Survey Adverse Childhood Experience Module


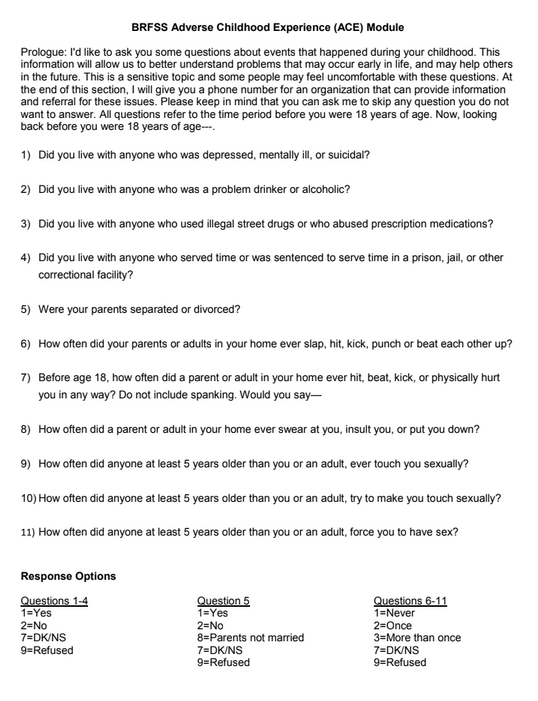


## 16.16 Social Support Survey


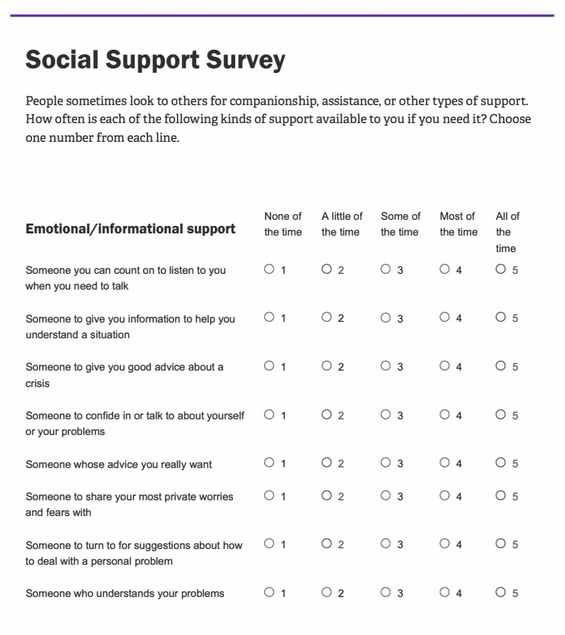


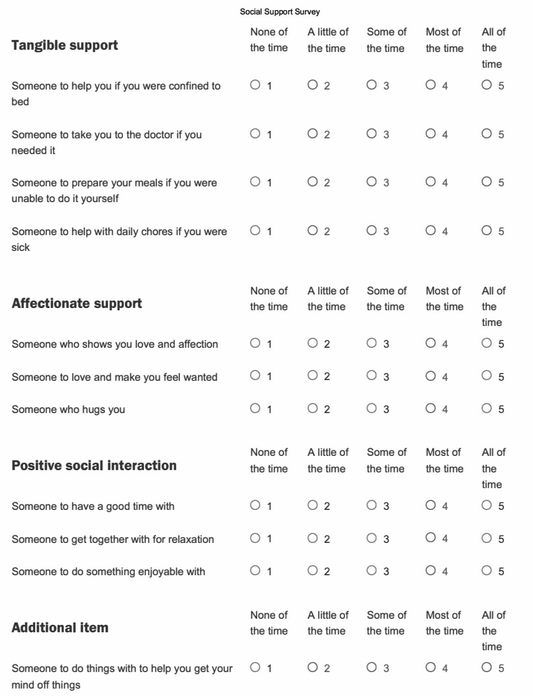


## 16.17 Patient Health Questionnaire – 9


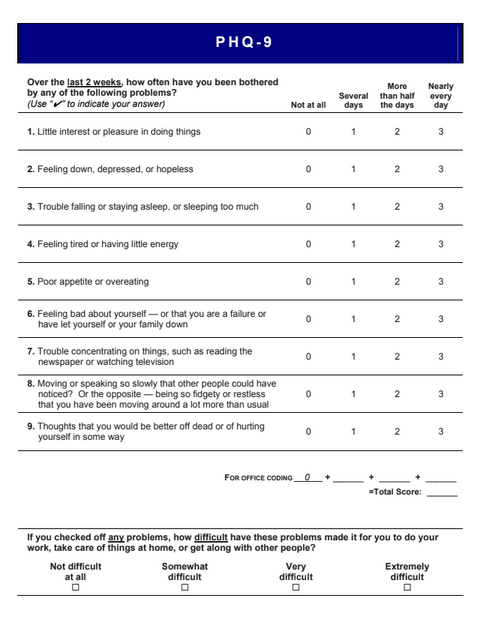


## 16.18 PTSD Checklist for DSM-5 (PCL-5)


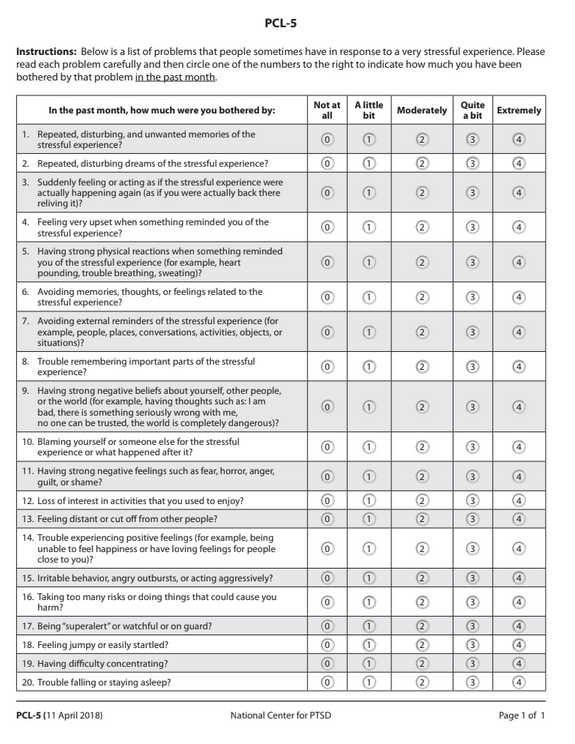


## 16.19 General Well-Being Schedule


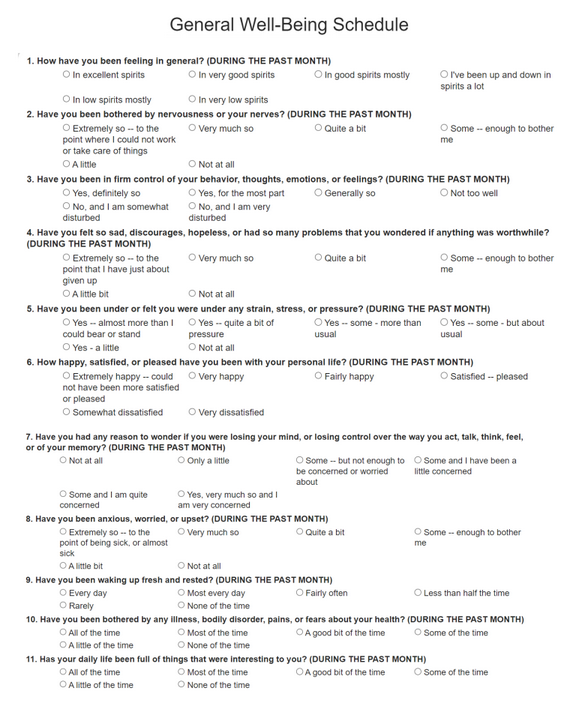


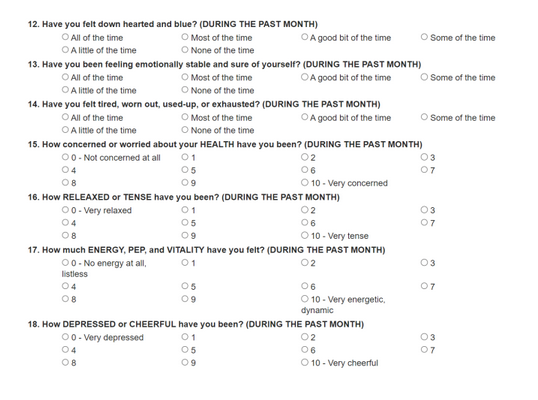


## 16.20 Generalized Anxiety Disorder – 7


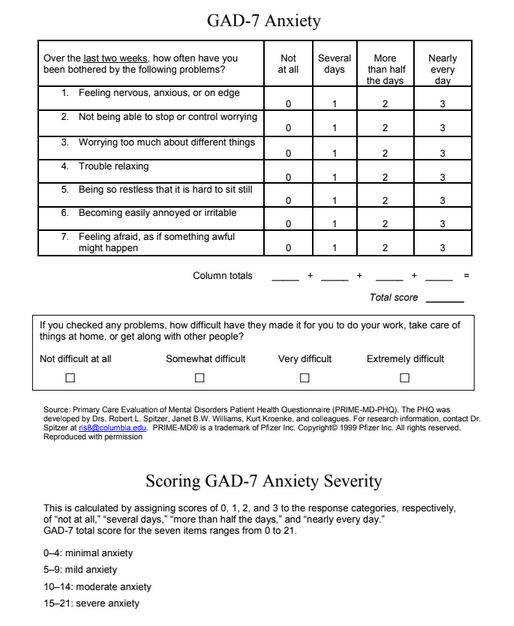


## 16.21 Alcohol Use Disorders Identification Test


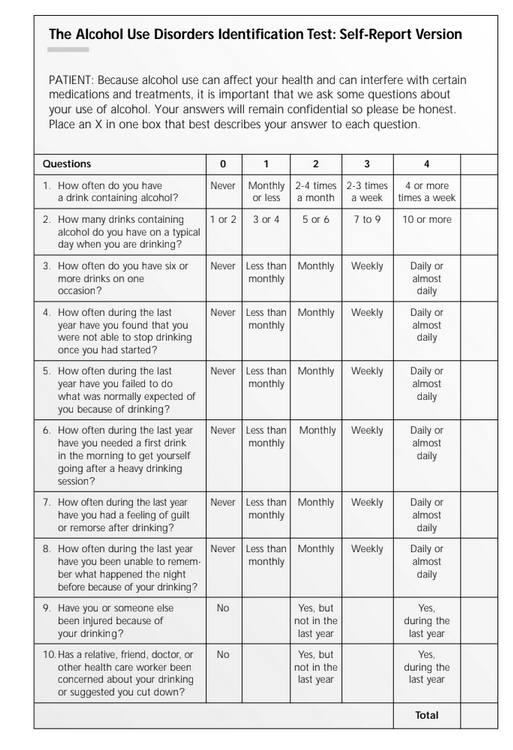


## 16.22 Clinician-Administered PTSD Scale (CAPS-5)

N.B: Below is an example of the type of questions that are asked as part of the CAPS-5 assessment.


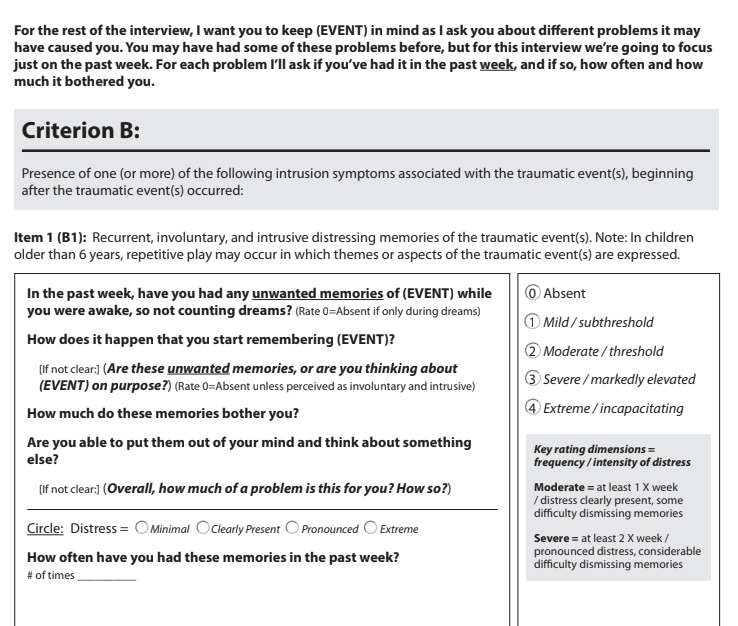


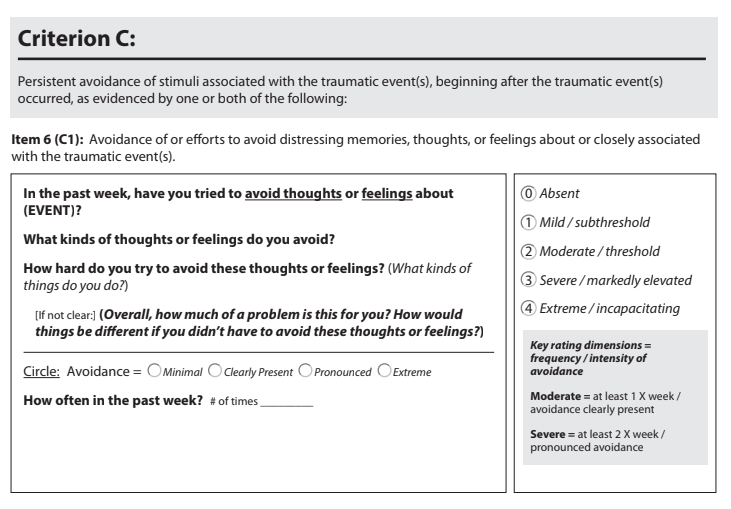


## 16.23 Personally Identifiable Information for the generation of NDA Global Unique Identifier (GUID)

N.B This data will be collected and stored in a separate NDA REDCap database, not within with the STOP trial study database. Participant REDCap records within the STOP trial study database will only contain the generated GUID, which will be used to pull de-identified data from the STOP trial study database to the NDA database. The following information is required to generate a GUID:

- First Name
- Middle Name
- Last Name
- Sex
- Date of Birth
- City/Municipality of Birth
