# Supplementary material for: The Silexan in the Treatment Of Posttraumatic stress disorder (STOP) trial: protocol for a 12-week randomised controlled trial of adjunctive Silexan for PTSD
Source: BMC Complement Med Ther. 2026 Feb 24;26:122. doi: 10.1186/s12906-026-05312-7 (PMC13041431; doi:10.1186/s12906-026-05312-7)
Supplement: Supplementary file 2 — Supplementary Material 2: STOP Trial Statistical Analysis Plan, version 2. [file 12906_2026_5312_MOESM2_ESM.docx]

Statistical Analysis Plan (SAP)

Prepared on behalf of Silexan in the treatment of Post-Traumatic Stress Disorder (STOP) trial

Author: Dr. Ravi Iyer

ORCID: 0000-0001-7699-0846

Date: 29/January/2026

Study Identifier

NCT06412757

# Table of Contents

[1. Table of Contents 2](#_Toc220681825)

[2. Abbreviations 5](#_Toc220681826)

[3. Administrative Information 6](#_Toc220681827)

[3.1. Title 6](#_Toc220681828)

[3.2. Trial Registration 6](#_Toc220681829)

[3.3. SAP version 6](#_Toc220681830)

[3.4. Protocol version 6](#_Toc220681831)

[3.5. SAP revisions 6](#_Toc220681832)

[3.6. Roles and responsibility e.g. key personnel; Chief investigator, Biostatistician 6](#_Toc220681833)

[3.7. Signatures of those involved 7](#_Toc220681834)

[4. Introduction 8](#_Toc220681835)

[4.1. Background and rationale 8](#_Toc220681836)

[4.2. Primary Aim 9](#_Toc220681837)

[5. Study Methods 10](#_Toc220681838)

[5.1. 9. Trial design 10](#_Toc220681839)

[5.2. Randomization 10](#_Toc220681840)

[5.3. Sample size 10](#_Toc220681841)

[5.4. Hypothesis testing framework 11](#_Toc220681842)

[5.5. Timing of final analysis 11](#_Toc220681843)

[5.6. Timing of outcome assessments 11](#_Toc220681844)

[6. Statistical Principles 14](#_Toc220681845)

[6.1. Confidence intervals and P values 14](#_Toc220681846)

[6.2. Protocol deviations 14](#_Toc220681847)

[6.3. Analysis populations 14](#_Toc220681848)

[6.3.1. Intention-to-treat population 14](#_Toc220681849)

[6.3.2. Per-protocol population 14](#_Toc220681850)

[6.3.3. Safety population 14](#_Toc220681851)

[7. Trial Population 16](#_Toc220681852)

[7.1. Eligibility 16](#_Toc220681853)

[7.1.1. Inclusion criteria 16](#_Toc220681854)

[7.1.2. Exclusion criteria 16](#_Toc220681855)

[7.2. CONSORT flow-chart 18](#_Toc220681856)

[7.3. Withdrawal/follow-up 18](#_Toc220681857)

[7.4. Baseline patient characteristics 18](#_Toc220681858)

[8. Analysis Outcome definitions 20](#_Toc220681859)

[8.1. Outcome definitions 21](#_Toc220681860)

[8.2. Outcome transformations 27](#_Toc220681861)

[8.3. Analysis methods 27](#_Toc220681862)

[8.3.1. Sensitivity analysis 29](#_Toc220681863)

[8.3.2. Actigraphy analysis 30](#_Toc220681864)

[8.3.3. Activity 31](#_Toc220681865)

[8.3.4. Heart Rate 31](#_Toc220681866)

[8.3.5. Sleep 32](#_Toc220681867)

[8.3.6. Proportion in remission 32](#_Toc220681868)

[8.4. Subgroup analyses 33](#_Toc220681869)

[8.5. Missing data 33](#_Toc220681870)

[8.6. Analysis of attrition 34](#_Toc220681871)

[8.7. Statistical software 34](#_Toc220681872)

[9. References 35](#_Toc220681873)

[10. Appendix 1: Proposed tables and figures 37](#_Toc220681874)

# Abbreviations

| **Acronym** | **Description** |
| --- | --- |
| ANOVA | Analysis of Variance |
| AQoL-6D | Assessment of Quality of Life-6D |
| AUDIT | Alcohol Use Disorders Identification Test |
| BDI-II | Beck Depression Inventory - II |
| BPD | Bipolar Personality Disorder |
| BRFSS-ACE | Behavioural Risk Factor Surveillance Survey Adverse Childhood Experience Module |
| CAPS-5 | Clinician-Administered PTSD Scale for DSM-5 |
| CCSM | DSM-5 Level 1 Cross-Cutting Symptom Measure |
| CI | Confidence Interval |
| GAD-7 | Generalised Anxiety Disorder-7 |
| GWBS | General Well Being Schedule |
| HAM-A | Hamilton Anxiety Rating Scale |
| ITT | Intention-To-Treat |
| MAR | Missing At Random |
| MCAR | Missing Completely At Random |
| MICE | Multiple Imputation using Chained Equations |
| MNAR | Missing Not At Random |
| PHQ-9 | Patient Health Questionnaire-9 |
| PHQ-15 | Patient Health Questionnaire-15 |
| PSQI | Pittsburgh Sleep Quality Index |
| PSQI-A | Pittsburgh Sleep Quality Index Addendum |
| PTSD | Post-Traumatic Stress Disorder |
| RCT | Randomised Controlled Trial |
| *sd* | Standard Deviation |
| SNRI | Selective Noradrenaline Reuptake Inhibitors |
| SSRI | Selective Serotonin Reuptake Inhibitors |
| SSS | Social Support Survey |
| WHODAS 2.0 | World Health Organization (WHO) Disability Assessment Schedule |

# Administrative Information

## Title

Silexan in the treatment of Post-Traumatic Stress Disorder (STOP) trial

## Trial Registration

ClinicalTrials.gov: NCT06412757

## SAP version

| *SAP Version* | *Date of authorship* |
| --- | --- |
| 1. | 28/05/2025 |
| 2. | 29/01/2026 |
| 3. |  |

## Protocol version

Version 9.0 28/11/2025

## SAP revisions

| *SAP Revision Date* | *Details of justification* |
| --- | --- |
| 29 JAN 2026 | Revision to align with Protocol version 9 |
|  |  |
|  |  |

## Roles and responsibility e.g. key personnel; Chief investigator, Biostatistician

| *Name* | *Affiliation* | *Contributor Role* |
| --- | --- | --- |
| 1. | Professor Michael Berk | Coordinating Investigator |
| 2. | Dr. Greg Roebuck | Principal Investigator |
| 3. | Dr. Georgia Parkin | Clinical Trial Co-ordinator |
| 4. | Associate Professor Mohammadreza Mohebbi | Senior Statistician |

## Signatures of those involved

| *Representative* | 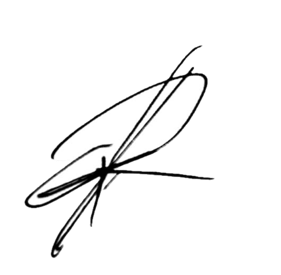*Signature* |
| --- | --- |
| Person writing the SAP | Dr Ravi Iyer |
| Senior statistician responsible | 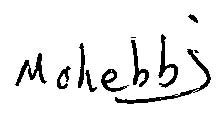Associate Professor Mohammadreza Mohebbi |
| Chief Investigator/clinical lead | Professor Michael Berk  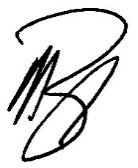 |

# Introduction

## Background and rationale

Post-Traumatic Stress Disorder (PTSD) is a debilitating psychiatric disorder characterised by re-experiencing symptoms, avoidance of trauma-related stimuli, deterioration in mood and cognition and alterations in reactivity and arousal. Serving military members and veterans are at higher risk of developing PTSD. PTSD is associated with high levels of disability across all areas of functioning and higher healthcare costs per patient than depression. PTSD is a significant factor contributing to veteran’s high rates of disability and elevated suicide risk.

Evidence-based clinical practice guidelines agree that the most effective treatment for PTSD is trauma-focussed psychotherapy. However, trauma-focused psychotherapy is associated with high rates of drop-out and lack of meaningful clinical improvement. Most veterans go on to retain their PTSD diagnosis after treatment.

A range of pharmacological interventions have been established including Selective Serotonin Reuptake Inhibitors (SSRIs), Selective Noradrenaline Reuptake Inhibitors (SNRIs) and atypical antipsychotics. Although these interventions have demonstrated some reduction in PTSD symptomatology, meta-analyses have revealed small effect sizes.

The low tolerability and high non-response rates of trauma-focussed psychotherapy and the limited efficacy and significant adverse effects associated with current PTSD pharmacotherapy underline the urgent need for new pharmacological treatments for PTSD that are effective and well tolerated.

Silexan is a standardized lavender oil preparation produced by steam distillation of *Lavandula* *angustifolia* flowers. Silexan has dose-dependent anxiolytic effects in both animal and human trials. A moderate effect size (Cohen’s *d*=0.5) has been demonstrated when Silexan 160mg is compared to inactive placebo in lessening symptoms of anxiety. A recent independent network meta-analysis by Yap and colleagues pooled data from five double-blind RCTs involving a total of 1,320 participants with GAD and subthreshold anxiety disorders. It found that Silexan 160 mg was superior to Silexan 80 mg, paroxetine 20 mg, lorazepam 0.5 mg and placebo in reducing anxiety symptoms.

The pathophysiology of PTSD is believed to involve maladaptive changes in the functioning of neural circuits responsible for fear and anxiety responses. It appears to involve fear conditioning in response to severe trauma, leading to an association between trauma-related stimuli and anxiety responses. It also appears to involve impairments in fear extinction, the process by which conditioned fear is unlearned. PTSD sufferers display acute anxiety in response to trauma-related cues. They also experience chronic anxiety symptoms, including hypervigilance, irritability, impaired concentration, poor sleep and exaggerated startle response. The foreground presence of anxiety in the pathophysiology and symptomatology of PTSD suggests that Silexan may be useful in the treatment of PTSD.

## Primary Aim

The primary aim of this randomised controlled trial will be to investigate the effectiveness of adjunctive Silexan, compared with placebo, over 12-weeks in improving PTSD symptoms in adults with PTSD.

# Study Methods

## 9. Trial design

STOP is a phase-3, 12-week, parallel-arm, double-blind, randomised, placebo-controlled trial. The study will employ a 2 (Experimental treatment: Silexan, placebo) x 3 (Assessment point: baseline, week 12, and week 16 [4 weeks post-treatment]) design.

Participants will be randomly allocated to either Silexan 160mg daily in addition to usual prescribed medications or an identical appearing inactive placebo in a 1:1 assignment ratio.

Silexan is available in orally administered immediate-release soft capsules. The main constituents are the monoterpenoids linalool (36.8%) and linalyl acetate (34.2%) additional to significant concentrations of eucalyptol, camphor, borneol, lavandulyl acetate, terpineol and caryophyllene. Silexan is manufactured in accordance with the lavender oil monograph in the European Pharmacopoeia, ensuring consistency in its composition.

Placebo will be manufactured to be identical in appearance to the Silexan capsules. In addition, the lavender odour of the Silexan capsules when opened will be matched by adding 0.08 mg of lavender oil to each placebo capsule (for a total of 0.16 mg daily or 0.1% of the dose for the active intervention group).

## Randomization

Permuted block-randomisation will be used to allocate participants to treatment arm. Block sizes will be selected at random from sizes *n*={2, 4 & 6}.

## Sample size

Power analysis by simulation was used to calculate the target sample size. Using the ANOVA_design function provided by the r-package ‘Superpower’(1), the STOP trial was simulated 10,000 times for each sample size within a range of *n*={50 to 150} participants per treatment arm in increments of 5 participants. In this simulation, type-I error = 0.05, a moderate effect size (Cohen’s *d*) = 0.25 and correlation between assessment timepoints = 0.8 resulted in power = 80.84% with 65 participants per treatment arm. This initial sample size was further inflated by an anticipated attrition rate = 10% to realise a final target sample size of 278 participants overall (139 per treatment arm).

## Hypothesis testing framework

All hypothesis tests will evaluate using a superiority framework with two-tailed assessment of significance, *p*<0.05.

## Timing of final analysis

All outcomes will be analysed following database lock and trial completion. The STOP trial will be completed once all recruited participants have completed the final week-16 follow-up assessment.

## Timing of outcome assessments

The primary outcome measure (i.e., CAPS-5) will be administered at baseline and week 12.

The secondary outcome measures (i.e., HAM-A, BDI-II, GWBS, WHODAS and AQoL-6D) will be administered at baseline and weeks 12 and 16.

The secondary outcomes PSQI & PSQI-A, PHQ-15, AUDIT, PHQ-9, GAD-7 will be administered at baseline and again at weeks 4, 8, 12 and 16.

The secondary outcomes PCL-5 and PGIC will be administered at weeks 2, 4, 6, 8 and 12. The PCL-5 will additionally be administered at intake and at week 16.

The BRFSS-ACE, CCSM, SSS will be administered at baseline only. The DRRI-2 will also be administered at baseline to individuals who have served in the military.

Actigraphy (activity, heart rate, sleep and temperature) will be continuously assessed from baseline to week-16.

A schedule of assessments is summarised in Table 2.

Table 2 Summary of assessments and corresponding timepoints of administration

|  | | Time point (Week, +/- 3 days) | | | | | | | |
| --- | --- | --- | --- | --- | --- | --- | --- | --- | --- |
| Time point (Week): | | T -1  Eligibility Screen (Intake Assessment) | T0  Baseline Assessment | Intervention | | | | | Follow-up/Off-Treatment |
|  |  |  |  | T2 | T4 | T6 | T8 | T12 | T16  Follow-up |
| Clinician/Researcher Administered / Participant Self-reported Measures | | | | | | | | | |
| **Electronic Consent to Screen** | | X |  |  |  |  |  |  |  |
| Inclusion/  Exclusion criteria | | X |  |  |  |  |  |  |  |
| MINI-7 |  | X |  |  |  |  |  |  |  |
| MSI-BPD | | X |  |  |  |  |  |  |  |
| LEC-5 | | X |  |  |  |  |  |  |  |
| Criterion A | | X |  |  |  |  |  |  |  |
| PCL-5 | | X |  |  |  |  |  |  |  |
| **Written Informed Consent** | |  | X |  |  |  |  |  |  |
| CAPS-5 | |  | X |  |  |  |  | X | X |
| HAM-A | |  | X |  |  |  |  | X | X |
| BDI-II | |  | X |  |  |  |  | X | X |
| BRFSS ACE | |  | X |  |  |  |  |  |  |
| CCSM | |  | X |  |  |  |  |  |  |
| Pregnancy test* | |  | X |  |  |  |  |  |  |
| DRRI-2** | |  | X |  |  |  |  |  |  |
| SSS | |  | X |  |  |  |  |  |  |
| GWBS | |  | X |  |  |  |  | X | X |
| WHODAS | |  | X |  |  |  |  | X | X |
| AQoL-6D | |  | X |  |  |  |  | X | X |
| PSQI & PSQI-A | |  | X |  | X |  | X | X | X |
| PHQ-15 | |  | X |  | X |  | X | X | X |
| AUDIT | |  | X |  | X |  | X | X | X |
| PHQ-9 | |  | X |  | X |  | X | X | X |
| GAD-7 | |  | X |  | X |  | X | X | X |
| PCL-5 | |  |  | X | X | X | X | X | X |
| PGIC | |  |  | X | X | X | X | X |  |
| Actigraphy and physiological measures | |  |  | X | X | X | X | X | X |
| Query of Adverse Events*** | |  |  | X | X | X | X | X | X |
| CGI | |  | X |  |  |  |  | X | X |
| Allocation | |  | X |  |  |  |  |  |  |
| Dispensing of trial drug | |  | X |  |  |  |  |  |  |

# Statistical Principles

## Confidence intervals and P values

Unless stated otherwise, all statistical testing will be performed to a significance level of p<.05, with 95% Confidence Intervals (CI) reported where appropriate. All superiority hypotheses will be evaluated using a two-tailed hypothesis tests.

## Protocol deviations

Protocol deviations shall be defined as any treatment or assessment session unsuccessfully completed due to the any of the following:

adverse event,

discontinuation of treatment,

pregnancy,

cessation of effective contraception,

signs of allergic reaction

All protocol deviations will be reported by study ID, category, and date of deviation.

## Analysis populations

There will be three analysis populations, including Intention-to-treat (ITT), per-protocol and safety.

### Intention-to-treat population

All randomised participants will constitute the ITT population. Participants in this population will be analysed according to the treatment arm to which they were assigned, regardless of which treatment was received. All efficacy analyses will be based upon the ITT population.

### Per-protocol population

All participants as randomised who have completed both baseline and primary endpoint assessments and maintain attendance to 50% or greater (4 or more sessions, inclusive of baseline and primary endpoint assessment) within the trial as prescribed will constitute the per-protocol population.

### Safety population

All randomised participants who receive at least one treatment dose will constitute the safety population. Participants in this population will be analysed according to the treatment arm to which they were assigned, regardless of which treatment was received. All safety analyses will be based upon this safety population.

# Trial Population

## Eligibility

### Inclusion criteria

1. Age 18 years or over.

2. Fluent in English.

3. Meet DSM-5 criteria for PTSD, irrespective of occupation

(e.g first responder, police force, ex-military, civilian), determined using the MINI-7.

4. Have a score on the PCL-5 equal to or over 33.

### Exclusion criteria

1. Are currently serving in the Australian Defence Force

2. Lifetime history of a psychotic or bipolar disorder, or dissociative identity disorder.

3. Moderate or severe alcohol or other substance use disorder within 3 months of screening.

4. Active suicidal or homicidal ideation.

5. Borderline Personality Disorder (BPD).

6. Acute or unstable medical illness or other significant medical condition that would make participation in the trial unsafe or inappropriate.

7. Pregnancy, lactation or unwillingness to use an acceptable method of contraception (required for both males and females who are of reproductive potential and sexually active with partners of the opposite sex) through the duration of participants’ involvement in the study, up to and including week 16. Participants will also be advised not to donate egg or sperm during the study period.

8. Commencement of a trauma-focussed psychotherapy (including Prolonged Exposure, Cognitive Processing Therapy and Eye Movement Desensitisation and Reprocessing) within 3 months of screening.

9. Commencement or change in dose of psychoactive medications within 4 weeks of screening.

10. Participants will be asked not to initiate psychotherapy or change the dose of psychoactive medications during the study except in clinically urgent circumstances; if this becomes necessary, a decision will be made on a case-by-case basis with regard to retaining the participant or terminating participation.

11. Severe acquired brain injury.

12. Individual is not eligible for public mental health services due to their visa status in Australia or for any other reason.

13. Any other condition that in the opinion of the research team is likely to make completion of the trial requirements infeasible.

14. Inability to understand or speak English to the extent necessary to consent and complete the trial (researcher or clinician-determined)

## CONSORT flow-chart

Information to be included in the Consolidated Standards of Reporting Trials (CONSORT) Flow diagram will be as illustrated in Figure 1**.**

Figure 1: CONSORT Flowchart


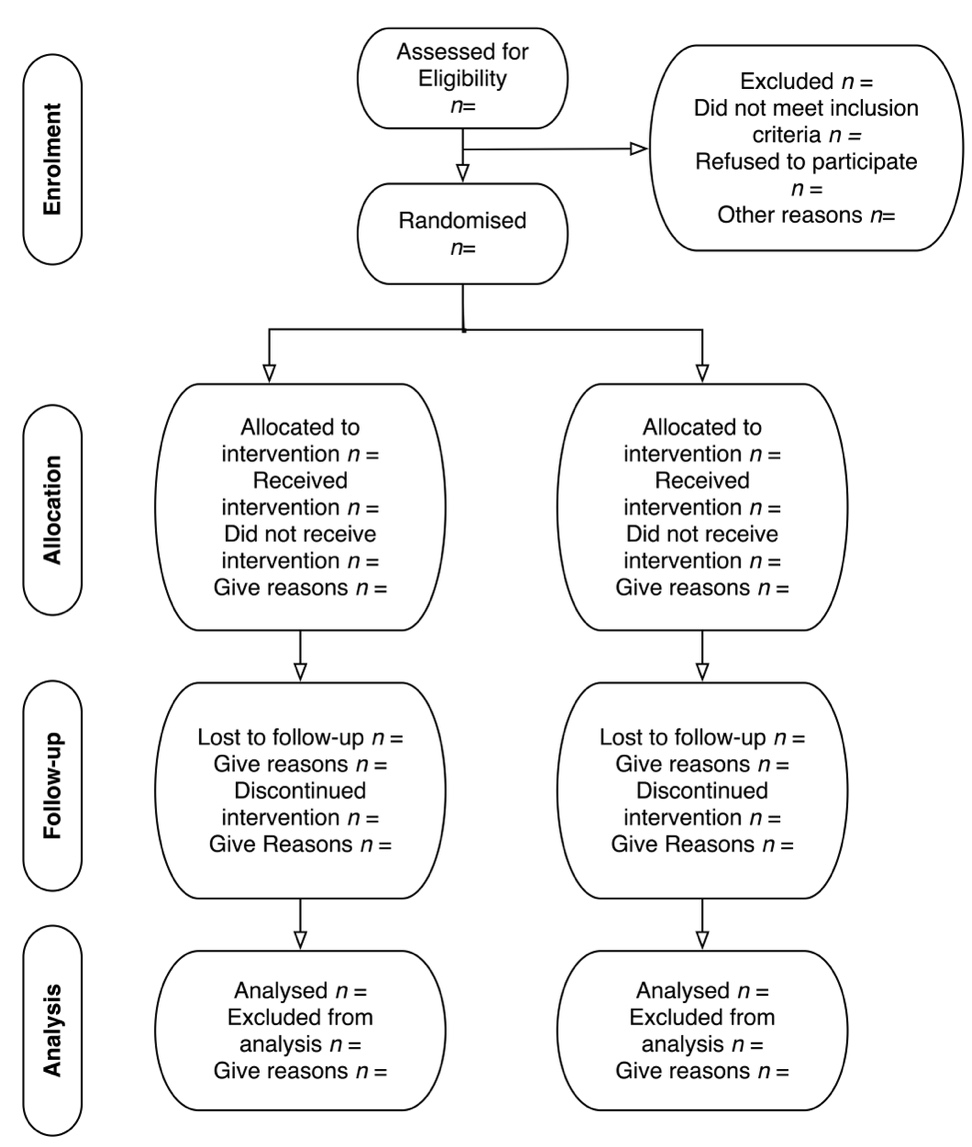


## Withdrawal/follow-up

The numbers of participants who completed all study treatment courses and participants who discontinued for any reason and at any time point will be reported. Reasons for discontinuation will be reported per treatment group and compared between treatment groups using a Fischer exact test. The number of days on study will be reported for all treated participants. Study treatment compliance for the ITT population will be summarised by treatment group and overall.

## Baseline patient characteristics

Participant characteristics will be summarised by treatment arm in terms of demographic variables, including biological sex at birth, gender identification, age, first nations status, highest level of education, employment status, marital status and CAPS-5 score at baseline.

Clinical characteristics at baseline will be presented for all participants, as randomised. The number of participants meeting all eligibility criteria and thus enrolled in the study, as well as the numbers of participant in each of the ITT, per-protocol and safety populations will be reported.

Demographic data, baseline participant characteristics, clinical characteristics, and actigraphy measurements will be summarised using descriptive statistics for the ITT, per protocol and safety populations, by treatment arm and overall.

Demographic and baseline clinical measures demonstrating significant differences between treatment arms will be added to the primary, secondary, and exploratory analysis models as covariates.

# Analysis Outcome definitions

**23. Primary Estimand**

The primary Estimand will be defined as:

*For a participant who meets DSM-5 criteria for Post-Traumatic Stress Disorder (PTSD) according to the Mini-International Neuropsychiatric Interview-7 (MINI-7) and has a PTSD Checklist for DSM-5 score ≥33 and meeting all other inclusion and exclusion criteria; what would be the expected effect of treatment with Silexan 160mg compared to placebo on PTSD symptom severity, as denoted by change in Clinician-Administered PTSD Scale for DSM-5 (CAPS-5) total symptom severity scores 12-weeks post-baseline, regardless of treatment discontinuation, missed treatments, protocol violations, adverse events and change in background medications.*

The primary estimand will be defined by the following five attributes:

Treatment condition of interest: Two treatment arms will be compared. Oral Silexan 160mg compared with matched in appearance inactive placebo.

Target Population: Adults aged 18 years and over who meet DSM-5 criteria for Post-Traumatic Stress Disorder (PTSD) according to the Mini-International Neuropsychiatric Interview-7 (MINI-7) and have a PTSD Checklist for DSM-5 score ≥33.

Endpoint: Change in marginal mean score on the CAPS-5 between baseline and week-12.

Population-level summary measure: Standardised mean difference (Cohen’s *d* effect size) between treatment conditions.

Intercurrent events and corresponding management All intercurrent events will be managed using a treatment policy strategy approach as defined in ICH E9 (R1)(3).

**24. Hypotheses**

The following two-sided superiority hypotheses will be evaluated:

- Silexan 160mg will significantly reduce PTSD symptoms on the CAPS-5 from baseline to week-12 in comparison to inactive placebo.

The null hypothesis is that there is no significant difference between treatment arms on the primary endpoint, 12-weeks post-baseline, as measured by the CAPS-5. The alternative hypothesis is that there will be a significant difference at two-sided *p*<.05 between Silexan 160mg and inactive placebo at the primary endpoint week-12 post-baseline, as measured by the CAPS-5:

$$H_{00}:\mu_{1}\leq\mu_{0};H_{01}:\mu_{1}>\mu_{0}$$

The same hypothesis testing approach will apply to all secondary outcome measures at week-12 post-baseline that include:

HAM-A, GAD-7, BDI-II, PHQ-9, PHQ-15, PSQI & PSQI-A, AUDIT, WHODAS, GWBS, AQoL-6D, PCL-5, all continuous actigraphy measures

The same hypothesis testing approach will apply to all secondary outcome measures at week-16 post-baseline that include:

CAPS-5, HAM-A, GAD-7, BDI-II, PHQ-9, PHQ-15, PSQI, PSQI-A AUDIT, WHODAS, GWBS and AQoL-6D, PCL-5

For the repeated measures hypotheses, the following hypotheses will apply:

$$H_{10}:\mu_{1, t}=\mu_{0, t}; H_{10}:\mu_{1, t}\neq\mu_{0, t}, where t=assessment timepoints$$

This hypothesis testing framework will apply to the following outcome measures at weeks 4, 8 and 12, including:

PHQ-9, PHQ-15, AUDIT, GAD-7, PSQI, PSQI-A, and all actigraphy measures.

The repeated measures testing framework will apply to the following outcome measures at weeks 2, 4, 6, 8 and 12, including:

PCL-5 and PGIC.

## Outcome definitions

The following table lists all primary and secondary outcome measures and details the methods used to calculate aggregate scores.

| ***Primary/Secondary variable*** | ***Proposed date for outcome measurement*** | ***Details of outcome measures*** |
| --- | --- | --- |
| Clinician-Administered PTSD Scale for DSM-5 (CAPS-5) total symptom severity score | Baseline, week 12 | 30-item clinician-rated measure of severity of PTSD symptoms according to the DSM-V classification criteria. Items are rated using a 5-point scale from 0=’absent’ to 4=’extreme/incapacitating’. The total symptom severity score is obtained by summing the individual severity scores for the first 20 items (4). |
| Hamilton Anxiety Rating Scale (HAM-A) | Baseline, weeks 12 & 16 | 14-item clinician-rated measure of psychological and somatic anxiety. Each item is rated using a Likert-style scale from 0=” not present” to 4=”very severe”. Total score is the summation of all individual item scores and ranges from 0-56. Total scores <17 = mild anxiety; 18-24 = mild to moderate anxiety; 25-30 = moderate to severe anxiety; and >30 = severe anxiety (5). |
| Clinical Global Impression Scale (CGI) | Baseline, weeks 12 & 16 | Clinician-rated measure of patient’s global functioning before and after initiation of study treatment. The CGI is comprised of three single item scores: severity of illness that is rated on a seven-point visual analogue scale from 1=’normal, not at all’ to 7 = ‘among the most extremely ill patients’ and global improvement that is rated on a seven-point visual analogue scale from 1=’very much improved’ to 7=’very much worse’. The third item is an Efficacy index that considers both therapeutic effectiveness and adverse reactions and is rated using four-point therapeutic effect by four-point side effect grid from 01 =’marked improvement and no side-effects’ to 16=’unchanged or worse and side effects outweigh the therapeutic effects’ (6). |
| Beck Depression Inventory – II (BDI-II) | Baseline, weeks 12 & 16 | 21-item self-report measure of depression severity. Each item is rated using a four-point scale from 0 to 3, which align with statements about mood, such as 0= ‘I do not feel sad’ to 3= ‘I am so sad or unhappy that I can’t stand it’. The total score is the summation of individual item scores, and ranges from 0-63 (7). |
| DSM-5 Level-1 Cross-cutting symptom measure (CCSM) | Baseline only | 23-item self or clinician rated measure that assesses 13 mental health domains that are important across psychiatric diagnoses. It is intended to assist clinicians identify additional areas of inquiry that may impact treatment and prognosis. Each item is rated on a 5-point scale ranging from 0=’none or not at all’ to 4=’severe or nearly every day’.  Items 1 & 2 contribute to the Depression domain; item 3 contributes to the Anger domain; items 4 & 5 contribute to the Mania Domain; Items 6, 7 & 8 contribute to the Anxiety domain; items 9 & 10 contribute to the Somatic Symptoms domain; item 11 contributes to the Suicidal Symptoms domain; items 12 & 13 contributes to the Psychosis domain; item 14 contributes to the Sleep Problems domain; item 15 contributes to the Memory domain; items 16 & 17 Repetitive Thoughts and Behaviour; item 18 contributes to the Dissociation domain; items 19 & 20 contributes to the Personality Function domain; and items 21, 22 and 23 contribute to the Substance Use domain.  Reportable measures include the frequency of highest rated item by domain; mean and sd of the summed scores within each domain (8). |
| World Health Organisation Disability Assessment Schedule (WHODAS 2.0) | Baseline, weeks 12 & 16 | 12-item self-rated measure of disability in adults aged 18 years and over. Each item is rated on a 5-point scale ranging from 0=’none’ to 4=’extreme or cannot do’. Total scores are derived from the summation of individual item scores and range from 0-48 with higher scores indicating higher levels of functional disability (9). |
| Behavioural Risk Factor Surveillance System (BRFSS) | Baseline only | 11-item self-report questionnaire that evaluates the presence of a history of childhood emotional/physical abuse, household disfunction and sexual abuse. Items 1-5 are rated on a dichotomous scale with 1=yes, 2=no. Question 5 also includes the option of ‘parents not married’. Items 6-11 are rated on a three-point scale with 1=’never’, 2=’once’ and 3=’more than once’. 7=’don’t know/not known’ and 9 = ‘refused / prefer not to answer’ being possible answers for all items. Items 1-5 = household dysfunction; items 6-8 = emotional/physical abuse; and items 9-11 = sexual abuse. Domain scores are the summation of individual item scores. These categories may be dichotomised to convey the presence or absence of exposure (10). |
| Pittsburgh Sleep Quality Index (PSQI) | Baseline and weeks 4, 8, 12 & 16 | 7-item self-report scale measuring the presence of acute sleep disturbance. Items are rated on a 4-point scale from 0 = ‘not during the past month’ to 3= ‘three or more times a week’. Component scores are derived from the coding and summation of individual item scores. Each component score has a range of 0 - 3, with higher scores indicating higher levels of acute sleep dysfunction (11). |
| Pittsburgh Sleep Quality Index Addendum for PTSD (PSQI-A) | Baseline and weeks 4, 8, 12 & 16 | 10 questions additional to the PSQI that relate to the frequency of anxiety and anger accompanying disruptive nocturnal behaviours and the timing of these events during the night. These items are included for clinical and informative purposes only (12). |
| PTSD checklist for DSM-V (PCL-5) | Intake, weeks 2, 4, 6, 8, 12 & 16 | 20-item self-report scale measuring the level of bother caused by a range of common symptoms associated with PTSD. Symptoms are scored on a 5-point Likert scale ranging from 0-4 (0 = Not at all, 1 = A little bit, 2 = Moderately, 3 = Quite a bit, 4 = Extremely). Items are queried relating to the past month at intake, and past two weeks at week 2, 4, 6, 8, 12 and 16 of the intervention. Items are summed to provide a total severity score (range 0-80) (13). |
| Patient Global Impression of Change (PGI-C) | Weeks 2, 4, 6, 8, 12 | Single-time participant-report measure of subjective change in symptom severity. The item is rated on a 7-point scale from 1=’No change (or condition has gotten worse)’ to 7=’A great deal better and a considerable improvement that has made all the difference’ (14). |
| Patient Health Questionnaire – 15 (PHQ-15) | Baseline and weeks 4, 8, 12 & 16 | 15-item participant rated measure of depressive symptom severity. Individual items are rated using a 4-point scale from 0=’not bothered at all’ to 2=’bothered a lot’. Total scores are derived from the summation of individual item scores and range between 0-30, with higher scores indicating higher levels of depressive symptom severity (15). |
| Deployment Risk and Resilience Inventory – 2 (DRRI-2) | Baseline only | Five modules of the DRRI-2 will be utilised in this study. All items are participant-rated. Section C: deployment environment has 14-items that are rated on a 5-point scale from 1=’almost none of the time’ to 5=’almost all of the time’. Section D: Combat experience and Section E: Postbattle Experiences have 17-items and 13-items respectively that are rated on a 6-point scale from 1=’never’ to 6=’daily or almost daily’. Section F: Exposure to Nuclear, Biological, or Chemical Agents has 13-items that are rated as either ‘Yes, I was exposed’, ‘No, I was not exposed’, or ‘I’m not sure if I was exposed’. Section G: Deployment Concerns has 12-items that are rated on a 5-point scale from 1=’Strongly disagree’ to 5=’Strongly Agree’. Section C, D, E and G total scores are derived from the summation of individual item scores, with higher scores suggesting higher levels of environment threat and combat experience respectively. Section F items are scored dichotomously (16). The DRRI-2 is only completed by participants who have previously served in the military. |
| Assessment of Quality of Life – 6D (AQoL-6D) | Baseline and weeks 12 & 16 | 20-item participant-rated measure of quality of life. Items are rated on a 4- to 6--point scales which align with statements of ability. For example, 1=’ I can do all these tasks very quickly and efficiently without any help’ to 5=’I can do none of these tasks by myself.’ Used as a psychometric measure, the total score is derived from the summation of individual item scores, and ranges from 20-99, with higher total scores indicating lower levels of quality of life (17). |
| Social Support Survey (SSS) | Baseline only | 19-item participant rated measure of level of social support within 4 domains. Items are rated on a 5-point scale from 1=’none of the time’ to 5=’all of the time’. The four domains are emotional/informational support (items 1-8); tangible support (items 9-12); affectionate support (items 13-15); and positive social interaction (items 16-18); plus an additional question (item 19). Individual domain total scores are derived from the mean of all individual domain related item scores. An overall index is derived from the mean of all 19 items. This overall index can be normalised to a range of 0-100 using the formula:  $100\times\frac{(observed score-minimum possible score)}{(maximum possible score-minimum possible score)}$ (18) |
| Patient Health Questionnaire (PHQ-9) | Baseline and weeks 4, 8, 12 & 16 | Nine-item participant rated measure of depressive symptom severity. Individual items are rated using a 4-point scale from 0=’not at all’ to 3=’nearly every day’. Total scores are derived from the summation of individual item scores and range between 0-27, with higher scores indicating higher levels of depressive symptom severity (19). |
| General Well Being Schedule (GWBS) | Baseline and weeks 12 & 16 | 18-item self-report measure of subjective psychological well-being and distress. Items 1-14 are rated using a 6-point scale, while items 16-19 are rated using a 11 item-point scale. Higher ratings indicate greater symptom severity. Items 2, 4, 5, 8, 10, 12, 14, 17, 18 are reverse coded. Total score is the summation of individual item scores, post-reverse scoring (20). |
| Generalised Anxiety Disorder -7 | Baseline and weeks 4, 8, 12 & 16 | 7-Item participant-rated measure of anxiety symptom severity. Items are rated on a 4-point Likert-Style scale, where 0 = “Not at all” and 3=”Nearly every day”. Total scores are calculated from the summation of individual item scores, and range between 0-21, with higher scores indicating higher anxiety symptom severity (21). |
| Alcohol Use Disorders Identification Test (AUDIT) | Baseline and weeks 4, 8, 12 & 16 | 10-item self-report measure of alcohol use severity. Items 1-8 are rated on a 5-point scale ranging from 0-4, while items 9 & 10 are rated on a 3–point scale with options 0, 2 or 4. Higher scores indicate higher levels of alcohol use disorder severity. Total AUDIT scores are the summation of individual item scores. Total score of 0-7=low-risk or abstinent drinking; 8-14=hazardous or harmful alcohol use that may pose risks to health and well-being; ≥15 indicates moderate-severe alcohol use disorder (22). |
| McLean screening instrument for Borderline Personality Disorder (MSI-BPD) | Intake assessment | 10-item self-report measure of symptoms of Borderline Personality Disorder. Items are rated on a dichotomous scale with 1=’yes’ and 0=’no’. Total scores are derived from the summation of individual item scores. Total scores ≥7 indicate the accepted clinical cutoff. |
| Actigraphy – Activity | Per minute; daily summary | Total time (min) that the participant is wearing, not wearing, and sleeping while wearing the watch; no. of minutes spent in sedentary, light, moderate and vigorous activity; vector magnitude of activity; no. of steps taken. |
| Actigraphy – Heart Rate | Assessments per millisecond, second, minute | Daily, daytime, nighttime heart rate (mean, median, *sd*, min, max, 25^th^, 75^th^, 95^th^ percentiles; daily, daytime, nighttime total time (min) normal cardiac rhythm, bradycardia, tachycardia; mean power in low (0.04-0.15hz) and high (0.15-0.4hz) frequency range; proportion that differ by >20ms, >50ms Heart Rate Variability; Root Mean Squared of successive differences Heart Rate Variability values per millisecond; Average breathing rate daily, daytime, nighttime. |
| Actigraphy – Sleep | Daily assessment | Total sleep time (min); Total light/deep sleep time; Total REM/non-REM sleep; Total time from sleep onset to wake onset; sleep fragmentation (higher values indicate greater fragmentation); normalised fragmentation. |
| Actigraphy - Temperature | assessments per minute | Mean body temperature per minute. |

Specific measurement and units (eg, glucose control, hbA1c [mmol/mol or %])

| *Outcome measure* | *Details of measurement and units* |
| --- | --- |
| Total sleep time | Continuous assessment; Minutes |
| Sleep efficiency | Global assessment; range 0-100% efficiency |
| Heart rate | Continuous assessment; BPM |
| Blood pressure | Continuous assessment; mmHg |
| Oxygen saturation | Continuous assessment; (SpO2)^2^ |
| Body temperature | Continuous assessment; degrees Celsius |
| Respiratory rate | Continuous assessment; number of inhalations per minute |
| Gait and step count | Continuous assessment; integer metric |

## Outcome transformations

Any calculation or transformation (e.g. reverse coding) used to derive the outcome

(eg, change from baseline, QoL score, time to event, logarithm, etc)

| *Outcome measure, item* | *Details of transformation* |
| --- | --- |
| General well-being measure (items 2, 4, 5, 8, 10, 12, 14, 17, 18) | Reverse coded |

## Analysis methods

The primary hypothesis will be evaluated using a 2-level Linear Mixed Model for Repeated Measures (MMRM) with fixed effects for treatment arm, assessment timepoint, and treatment arm by assessment timepoint interaction (level-1) and random intercept for individual participants (level-2). The analysis model will be adjusted for CAPS-5 scores obtained during the baseline assessment. Appropriate contrasts will be applied at the primary endpoint (i.e., week 12) to evaluate all relevant hypotheses.

The following general model will be used, indicating the participant response (e.g., CAPS-5), denoted by *y_ij_,* where *i*={1, … , *N*} repeated measurements and *j*={1, … , *J*} participants. In the following equation there is a random intercept applied to each participant, and fixed effects for treatment arm, time, relevant covariates, and treatment arm by assessment timepoint interaction.

$$y_{ij}=X_{ij}\beta+W_{j}\gamma+{\left( T_{j}\times A_{ij} \right)\delta+b}_{j}+\varepsilon_{ij}$$

Where:

*i*=1, …, *N*_j_ Repeated measurements per participant

*j*=1, … , 278 participants in the target sample size

*y*=an *N* x 1 vector of outcome measurements

*X*=*N* x *p* matrix of within-subject covariates

*W*=*J* x *q* matrix of between-subject covariates

*Z*=*N* x *J* design matrix of random effects

*T_j_*=the treatment arm (e.g., 0=control, 1=treatment)

*A_ij_*=Assessment timepoint (e.g., 2-weeks, … , 12-weeks)

$\left( T_{j}\times A_{ij} \right)\delta$*=the interaction effect between treatment and assessment timepoint*

*β*=*p* x 1 vector of fixed effects for within-subject predictors

γ=*q* x 1 vector of fixed effects for the between-subject predictors.

*b_j_~N*(0, τ^2^) random intercept per participant

ε_ij_~*N*(0, σ^2^) residual variance

An unstructured correlation matrix will be assumed in the primary analysis model (23). In the event of failure to converge, alternative covariance structures will be used including the heterogeneous Toeplitz and then compound symmetry correlation structures. Kenward-Rogers adjustment will be used to approximate denominator degrees of freedom and adjust standard errors, assuming homogeneity of variance that informs the use of a compound symmetry correlation structure, does not hold (23).

Departures from normality in the residuals of the primary model will be assessed using visual inspection (e.g., Q-Q plots) rather than formal statistical tests, as mixed models are generally robust to mild deviations from normality. Transformation of the outcome (e.g., Box-Cox) will only be considered if substantial non-normality or heteroscedasticity is observed in residuals and will be reported as a sensitivity analysis.

The primary analysis will compare treatment groups on CAPS-5 scores over time using a linear mixed model for repeated measures (MMRM), adjusting for CAPS-5 scores obtained at baseline. Change from baseline will also be summarized and analysed in a secondary model, and results will be interpreted in terms of clinically meaningful change. Model estimation will use restricted maximum likelihood.

The following tests/plots will be produced to ensure all assumptions underpinning the primary analysis model are not violated.

| Assumption | Test/plot |
| --- | --- |
| Linearity | Residuals vs. fitted plot, scatterplots |
| Normality of residuals | Histogram, Q-Q plot, Shapiro-Wilk test |
| Homoscedasticity | Residuals vs. Fitted plot, Leven’s test |
| Independence of residuals | ACF plot, Durbin-Watson test |
| Random effects | Q-Q plot of random effects, ICC, LRT |
| Multicollinearity | Variance inflation factor, correlation matrix |

The primary analysis model will evaluate the following measures:

CAPS-5: baseline to primary endpoint (week-12)

HAM-A: baseline to primary endpoint (week-12) and RCT follow-up (week-16)

BDI-II: baseline to primary endpoint (week-12) and RCT follow-up (week-16)

WHODAS: baseline to primary endpoint (week-12) and RCT follow-up (week-16)

GWBS: baseline to primary endpoint (week-12) and RCT follow-up (week-16)

AQoL-6D: baseline to primary endpoint (week-12) and RCT follow-up (week-16)

GAD-7: baseline to weeks 4, 8, primary endpoint (week-12) and RCT follow-up (week-16)

PHQ-9: baseline to weeks 4, 8, primary endpoint (week-12) and RCT follow-up (week-16)

PHQ-15: baseline to weeks 4, 8, primary endpoint (week-12) and RCT follow-up (week-16)

AUDIT: baseline to weeks 4, 8, primary endpoint (week-12) and RCT follow-up (week-16)

PSQI & PSQI-A: baseline to weeks 4, 8, primary endpoint (week-12) and RCT follow-up (week-16)

PCL-5: intake to weeks 2, 4, 6, 8, primary endpoint (week-12) and RCT follow-up (week-16)

PGIC: Weeks 2, 4, 6, 8, and primary endpoint (week-12)

### Sensitivity analysis

A sensitivity analysis will be conducted using a Generalized Estimating Equations (GEE) approach to assess the robustness of the primary analysis results derived from the MMRM. The GEE model will evaluate the treatment effect on CAPS-5 total score from baseline to week 12, using a population-averaged (marginal) framework. The model will include the following: CAPS-5 total score (Primary outcome measure), fixed effects for treatment group, assessment timepoint, baseline CAPS-5 score, and treatment × timepoint interaction; a unstructured working correlation structure, to account for correlations among repeated measures within individuals; Huber-White sandwich (robust) standard errors to protect against misspecification of the correlation structure; and an Identity link function. Analyses will be performed on observed data, assuming missing completely at random (MCAR) for valid inference.

The primary hypothesis will be tested using model-based contrasts at Week-12, matching the contrast from the primary MMRM analysis. This GEE analysis will serve to confirm whether the treatment effect observed in the MMRM model is robust to alternative modelling assumptions, particularly in relation to the covariance structure and residual distribution. If the GEE and MMRM models yield substantively different conclusions, further exploration of missing data mechanisms and potential model misspecification will be considered.

Additional sensitivity analyses using both the MMRM and GEE models will include the following comparisons:

ITT and per-protocol populations compared

ITT, with and without multiple imputation compared

### Actigraphy analysis

Continuous actigraphy data will be obtained for the full 16-week trial duration via the Actigraph LEAP wearable watches. Prior to analysis, several preprocessing steps will be undertaken including data trimming to remove actigraphy data outside the trial period.

Functional data analysis will be used to model all continuous activity, heart rate, sleep and temperature data. While controlling for baseline actigraphy level, sex, age and treatment arm and modelling individual participant variation using a random intercept, the following model will be used:

$$Y_{i}\left( t \right)=\sum_{k=1}^{K} c_{0k}B_{k}\left( t \right)+\sum_{k=1}^{K} c_{1k}B_{k}\left( t \right){Baseline}_{i}+\sum_{k=1}^{K} c_{2k}B_{k}\left( t \right){Age}_{i}+\sum_{k=1}^{K} c_{3k}B_{k}\left( t \right){Sex}_{i}+\sum_{k=1}^{K} c_{4k}B_{k}\left( t \right){TreatmentArm}_{i}+f_{i}(t)+\epsilon_{i}(t)$$

Where:

$Y_{i}\left( t \right)$=Activity level, heart rate, sleep, temperature

$B_{k}\left( t \right)$=basis function; either B-spline or Fourier basis

*K*=number of basis functions

$f_{i}(t)$=participant-specific random-intercept

$\epsilon_{i}(t)$=residual error

In this functional data analysis model, missing data is automatically interpolated.

A supplementary analysis using a Generalised Additive Mixed Model (GAMM) will be used to analyse all continuous actigraphy data. In this model, Partial intra-day missing data will be imputed using average day imputation. This method will use the mean of data that was collected. It is assumed that the underlying mechanism of missingness in this case will be Not Missing at Random (NMAR) and that periods of non-wear may correspond with increased symptom severity.

The following model will be used:

$$y_{ijt}=\beta_{0}+f\left( t \right)+\beta_{1}A_{jt}+\beta_{2}T_{i}+\beta_{3}{(A}_{jt}T_{i})+u_{i}+\epsilon_{ijt}$$

Where:

y_ijt_=actigraphy measure (e.g., number of steps taken) by participant i at minute t during each assessment period (e.g,. baseline – week 2).

*f*(*t*)=spline-based function for time of day

*A_ij_*=Assessment timepoint

*T_i_=treatment arm*

*A_ij_ x T_i_*=interaction effect between treatment arm and assessment timepoint

*u_i_*~*N(*0, σ^2^)=random intercept for each participant

ε_ijt_=autocorrelated residuals with an AR(1) structure

A functional data analysis approach will be used to evaluate the following outcome measures:

### Activity

Difference between treatment arms on total number of recorded minutes each day over the 12-week assessment period

Difference between treatment arms on total number of wear time minutes each day over the 12-week assessment period

Difference between treatment arms on total number of non-wear time minutes each day over the 12-week assessment period

Difference between treatment arms on total number of sleep time minutes each day over the 12-week assessment period

Difference between treatment arms on total number of sedentary activity minutes each day over the 12-week assessment period

Difference between treatment arms on total number of light activity minutes each day over the 12-week assessment period

Difference between treatment arms on total number of moderate activity minutes each day over the 12-week assessment period

Difference between treatment arms on total number of vigorous activity minutes each day over the 12-week assessment period

Difference between treatment arms on total number of steps each day over the 12-week assessment period

### Heart Rate

Difference between treatment arms on mean, median and *sd*, 25^th^, 75^th^, 95^th^ percentile during daytime, nighttime, from baseline to the primary endpoint (week-12)

Difference between treatment arms on total time in minutes heart rate is classified as normal cardiac rhythm, atrial fibrillation and tachycardic, daytime and nighttime, from baseline to the primary endpoint (week-12)

Difference between treatment arms on average root mean squared of the successive differences in heart rate variability, from baseline to the primary endpoint (week-12).

Difference between treatment arms on mean power in low frequency band (0.04-0.15hz), high frequency band (0.15-0.4hz), from baseline to the primary endpoint (week-12),

Difference between treatment arms on mean, median, *sd*, proportion >20ms, proportion >50ms of successive NN heart rate intervals (length of normal heart rate) each day, daytime, nighttime, from baseline to the primary endpoint (week-12).

Difference between treatment arms on mean breathing rate, from baseline to the primary endpoint (week-12).

### Sleep

Difference between treatment arms on total sleep time in minutes, from baseline to the primary endpoint (week-12).

Difference between treatment arms on total light sleep time in minutes, from baseline to the primary endpoint (week-12).

Difference between treatment arms on total deep sleep time in minutes, from baseline to the primary endpoint (week-12).

Difference between treatment arms on total REM sleep time in minutes, from baseline to the primary endpoint (week-12)

Difference between treatment arms on total non-REM sleep time in minutes, from baseline to the primary endpoint (week-12)

Difference between treatment arms on total time between sleep onset and wake onset in minutes, from baseline to the primary endpoint (week-12)

Difference between treatment arms on total wake bouts between sleep onset and wake onset, from baseline to the primary endpoint (week-12)

Difference between treatment arms on mean sleep bouts in minutes, from baseline to the primary endpoint (week-12)

Difference between treatment arms on sleep fragmentation, from baseline to the primary endpoint (week-12)

Difference between treatment arms on mean temperature, from baseline to the primary endpoint (week-12)

### Proportion in remission

A further supplementary analysis will be performed to evaluate the proportion of participants in each treatment arm that lose their PTSD diagnosis (i.e., achieve remission), as assessed using the CAPS-5, from baseline to week-12. This aim will be evaluated using a generalised linear model with log link as follows:

$$\Pr\left( Y_{i}=1 \right)=p_{i},\log\left( p_{i} \right)=\beta_{0}+\beta_{1}{Trt}_{i}+\beta_{2}{Diag}_{i}$$

Remission at Week 12 will be defined as no longer meeting DSM-5 PTSD diagnostic criteria on the CAPS-5 interview at the Week 12 visit. The primary analysis will be conducted in the intention-to-treat population using a generalized linear model with log link to estimate the risk ratio of remission for Silexan versus placebo, adjusting for baseline CAPS-5 total score. The treatment effect will be presented as a risk ratio with 95% confidence interval and two-sided p-value at the 5% significance level. Participants with missing Week 12 diagnostic status will be classified as not in remission in the primary analysis; sensitivity analyses will include logistic regression and analyses under multiple imputation with additional tipping-point assessments. For each group, the *n*/*N*(%) in remission at week-12, the Risk Ratio (RR) with 95% confidence intervals and the risk difference and number needed to treat will be reported.

## Subgroup analyses

The following subgroup analyses will be performed using the primary and supplementary analysis models.

- Do rates of response differ between participants who meet diagnostic criteria for PTSD according to the CAPS-5 and those who do not?
- Do rates of response differ between participants who were not taking any prescribed psychoactive medications prior to commencement of the study (i.e. participants who received Silexan as monotherapy) and participants who were taking other psychoactive medications (i.e. participants who received Silexan as an adjunct to their other medications)?
- Do rates of response differ between single PTSD diagnosis versus the presence of multiple comorbid diagnoses alongside PTSD?
- Do rates of response differ for participants with a history of childhood trauma versus no history of childhood trauma?
- Do rates of response differ between participants scoring above (≥) and below (<) the median MSI-BPD score?
- Do rates of response differ between participants who exhibit low/mild (<15) vs. moderate/severe anxiety (≥15) as measured using the HAM-A?
- Do rates of response differ between participants who were receiving psychotherapy at baseline and those who were not?

## Missing data

Analyses using the primary analysis model will be conducted using tipping-point analysis according to the delta-imputation method(24). In the first instance, missed assessment timepoint data, at any timepoint post-baseline assessment, will be addressed using Multiple Imputation using Chained Equations (MICE), under and assumption of Missing at Random (MAR). Following application of MICE to missing data, each imputed value will then be sequentially worsened clinically by adding a pre-specified amount (i.e., *δ*=1 CAPS-5 point). Additional delta-increments will then be added until the analysis model returns a non-significant result at the primary endpoint (i.e., denoting tipping point reached). The sum of all delta-increments prior to the tipping point will indicate the robustness of the primary analysis model results to assumptions of MAR, with higher values indicating greater robustness.

Missing data will be reported by treatment arm, measure and assessment timepoint.

## Analysis of attrition

Binary logistic regression will be used at each assessment time point to determine whether any demographic or baseline measures predict attrition (0=presence; 1=absence of data at each assessment timepoint). These variables be used to model the pattern of missingness under an assumption of Not Missing at Random (NMAR)

## Statistical software

A suitable statistical software will be used to carry out all analyses, including r, STATA or SAS.

# References

1. Caldwell A, Lakens D. Superpower: Simulation-Based Power Analysis for Factorial Designs. CRAN: Contributed Packages. 2020.

2. Edwards JM, Walters SJ, Julious SA. A retrospective analysis of conditional power assumptions in clinical trials with continuous or binary endpoints. Trials. 2023 Mar 22;24(1):215.

3. Committee for Medicinal Products for Human Use. ICH E9 (R1) addendum on estimands and sensitivity analysis in clinical trials to the guideline on statistical principles for clinical trials. Geneva; 2020. Report No.: EMA/CHMP/ICH/436221/2017.

4. National Centre for PTSD. Clinician-Administered PTSD Scale for DSM-5 (CAPS-5) [Internet]. 2018 [cited 2025 Mar 3]. Available from: https://www.mendeley.com/reference-manager/library/all-references/bda1ef11-69b3-3ffd-87b4-5b1df61b8714

5. Hamilton M. The assessment of anxiety states by rating. British Journal of Medical Psychology. 1959 Mar 12;32(1):50–5.

6. Busner J, Targum SD. The clinical global impressions scale: applying a research tool in clinical practice. Psychiatry (Edgmont). 2007 Jul;4(7):28–37.

7. Osman A, Kopper BA, Barrios F, Gutierrez PM, Bagge CL. Reliability and Validity of the Beck Depression Inventory--II With Adolescent Psychiatric Inpatients. Psychol Assess. 2004 Jun;16(2):120–32.

8. American Psychiatric Association. DSM-5 Self-Rated Level 1 Cross-Cutting Symptom Measure—Adult [Internet]. 2013 [cited 2025 Mar 3]. Available from: https://www.psychiatry.org/File%20Library/Psychiatrists/Practice/DSM/APA_DSM5_Level-1-Measure-Adult.pdf

9. World Health Organisation. WHO Disability Assessment Schedule 2.0 (WHODAS 2.0) [Internet]. 2025 [cited 2025 Mar 3]. Available from: https://www.who.int/standards/classifications/international-classification-of-functioning-disability-and-health/who-disability-assessment-schedule

10. Centre for Disease Control. Behavioural Risk Factor Surveillance System [Internet]. 2024 [cited 2025 Mar 3]. Available from: https://www.cdc.gov/brfss/data_documentation/index.htm

11. Shahid A, Wilkinson K, Marcu S, Shapiro CM. Pittsburgh Sleep Quality Index (PSQI). In: STOP, THAT and One Hundred Other Sleep Scales. New York, NY: Springer New York; 2011. p. 279–83.

12. Buysse DJ, Reynolds CF, Monk TH, Berman SR, Kupfer DJ. The Pittsburgh sleep quality index: A new instrument for psychiatric practice and research. Psychiatry Res. 1989 May;28(2):193–213.

13. U.S. Department of Veterans Affairs. PTSD: National Center for PTSD. 2024 Nov 20 [cited 2025 Mar 3]; Available from: https://www.ptsd.va.gov/professional/assessment/adult-sr/ptsd-checklist.asp

14. Ferguson L, Scheman J. Patient global impression of change scores within the context of a chronic pain rehabilitation program. J Pain. 2009 Apr;10(4):S73.

15. Kroenke K, Spitzer RL, Williams JBW. The PHQ-15: Validity of a New Measure for Evaluating the Severity of Somatic Symptoms. Psychosom Med. 2002 Mar;64(2):258–66.

16. Vogt D, Smith BN, King LA, King DW, Knight J, Vasterling JJ. Deployment Risk and Resilience Inventory‐2 (DRRI‐2): An Updated Tool for Assessing Psychosocial Risk and Resilience Factors Among Service Members and Veterans. J Trauma Stress. 2013 Dec 25;26(6):710–7.

17. Allen J, Inder KJ, Lewin TJ, Attia JR, Kelly BJ. Construct validity of the Assessment of Quality of Life - 6D (AQoL-6D) in community samples. Health Qual Life Outcomes. 2013;11(1):61.

18. RAND. Social Support Survey. 2025 [cited 2025 Mar 3]; Available from: https://www.rand.org/health-care/surveys_tools/mos/social-support.html

19. Kroenke K, Spitzer RL, Williams JB. The PHQ-9: validity of a brief depression severity measure. J Gen Intern Med. 2001 Sep;16(9):606–13.

20. Fazio AF. A concurrent validational study of the NCHS General Well-Being Schedule. Vital Health Stat 2. 1977 Sep;(73):1–53.

21. Spitzer RL, Kroenke K, Williams JBW, Löwe B. A Brief Measure for Assessing Generalized Anxiety Disorder. Arch Intern Med. 2006 May 22;166(10):1092.

22. Saunders JB, Aasland OG, Babor TF, de la Fuente JR, Grant M. Development of the Alcohol Use Disorders Identification Test (AUDIT): WHO Collaborative Project on Early Detection of Persons with Harmful Alcohol Consumption‐II. Addiction. 1993 Jun 24;88(6):791–804.

23. Gosho M, Maruo K. Effect of heteroscedasticity between treatment groups on mixed‐effects models for repeated measures. Pharm Stat. 2018 Sep 6;17(5):578–92.

24. Leacy FP, Floyd S, Yates TA, White IR. Analyses of Sensitivity to the Missing-at-Random Assumption Using Multiple Imputation With Delta Adjustment: Application to a Tuberculosis/HIV Prevalence Survey With Incomplete HIV-Status Data. Am J Epidemiol. 2017 Jan 10;

# Appendix 1: Proposed tables and figures

Baseline participant characteristics

Demographic characteristics by treatment arm, ITT population

Demographic characteristics by treatment arm, per protocol population

Demographic characteristics by treatment arm, safety population

Clinical characteristics at baseline by treatment arm, ITT population

Clinical characteristics at baseline by treatment arm, per protocol population

Clinical characteristics at baseline by treatment arm, safety population

Concomitant medications at baseline, ITT population

Concomitant medications at baseline, per protocol population

Concomitant medications at baseline, safety population

Efficacy

Summary of CAPS-5 total score by visit, ITT population

Summary of CAPS-5 total score by visit, sensitivity with MICE, ITT population

Summary of CAPS-5 total score by visit, sensitivity with ANCOVA, ITT population

Summary of HAM-A total score by visit, ITT population

Summary of GAD-7 total score by visit, ITT population

Summary of BDI-II total score by visit, ITT population

Summary of PHQ-9 total score by visit, ITT population

Summary of PHQ-15 total score by visit, ITT population

Summary of PSQI total score by visit, ITT population

Summary of PSQI-A total score by visit, ITT population

Summary of AUDIT total score by visit, ITT population

Summary of WHODAS total score by visit, ITT population

Summary of GWBS total score by visit, ITT population

Summary of AWQoL-6D total score by visit, ITT population

Summary of PCL-5 total score by visit, ITT population

Summary of PGIC total score by visit, ITT population

Summary actigraphy scores, ITT population

Levels of attrition by assessment timepoint and by treatment arm, ITT population

Levels of attrition by assessment timepoint and by treatment arm, per protocol population

Safety

Treatment emergent serious adverse events, reported using MedDRA hierarchy, ITT population

Figures

Change from baseline in CAPS-5 total scores by visit and treatment arm, ITT population

Daily change from baseline in mean actigraphy scores, ITT population
